# The *Apostasia* genome and the evolution of orchids

**DOI:** 10.1038/nature23897

**Published:** 2017-09-13

**Authors:** Guo-Qiang Zhang, Ke-Wei Liu, Zhen Li, Rolf Lohaus, Yu-Yun Hsiao, Shan-Ce Niu, Jie-Yu Wang, Yao-Cheng Lin, Qing Xu, Li-Jun Chen, Kouki Yoshida, Sumire Fujiwara, Zhi-Wen Wang, Yong-Qiang Zhang, Nobutaka Mitsuda, Meina Wang, Guo-Hui Liu, Lorenzo Pecoraro, Hui-Xia Huang, Xin-Ju Xiao, Min Lin, Xin-Yi Wu, Wan-Lin Wu, You-Yi Chen, Song-Bin Chang, Shingo Sakamoto, Masaru Ohme-Takagi, Masafumi Yagi, Si-Jin Zeng, Ching-Yu Shen, Chuan-Ming Yeh, Yi-Bo Luo, Wen-Chieh Tsai, Yves Van de Peer, Zhong-Jian Liu

**Affiliations:** 1 Shenzhen Key Laboratory for Orchid Conservation and Utilization, The National Orchid Conservation Center of China and The Orchid Conservation and Research Center of Shenzhen, Shenzhen, 518114 China; 2 Department of Plant Biotechnology and Bioinformatics, Ghent University, Gent, 9052 Belgium; 3 VIB Center for Plant Systems Biology, Gent, 9052 Belgium; 4 Orchid Research and Development Center, National Cheng Kung University, Tainan, 701 Taiwan; 5 Department of Life Sciences, National Cheng Kung University, Tainan, 701 Taiwan; 6 State Key Laboratory of Systematic and Evolutionary Botany, Institute of Botany, Chinese Academy of Sciences, Beijing, 100093 China; 7 College of Forestry, South China Agricultural University, Guangzhou, 510640 China; 8 Technology Center, Taisei Corporation, Nase-cho 344-1, Totsuka-ku, Yokohama, 245-0051 Kanagawa Japan; 9 Bioproduction Research Institute, National Institute of Advanced Industrial Science and Technology (AIST), Central 6, Higashi 1-1-1, Tsukuba, 305-8562 Ibaraki Japan; 10 PubBio-Tech Services Corporation, Wuhan, 430070 China; 11 Graduate School of Science and Engineering, Saitama University, 255 Shimo-Okubo, Sakura-ku, 338-8570 Saitama Japan; 12 NARO Institute of Floricultural Science (NIFS), 2-1 Fujimoto, Tsukuba, 305-8519 Ibaraki Japan; 13 Institute of Tropical Plant Sciences, National Cheng Kung University, Tainan, 701 Taiwan; 14 Department of Genetics, Genomics Research Institute, Pretoria, 0028 South Africa; 15 College of Landscape Architecture, Fujian Agriculture and Forestry University, Fuzhou, 350002 China; 16 The Center for Biotechnology and BioMedicine, Graduate School at Shenzhen, Tsinghua University, Shenzhen, 518055 China; 17 Present Address: Biotechnology Center in Southern Taiwan, Agricultural Biotechnology Research Center, Academia Sinica, 741 Tainan, Taiwan

**Keywords:** Genome, Speciation

## Abstract

Constituting approximately 10% of flowering plant species, orchids (Orchidaceae) display unique flower morphologies, possess an extraordinary diversity in lifestyle, and have successfully colonized almost every habitat on Earth^1,2,3^. Here we report the draft genome sequence of *Apostasia shenzhenica*^4^, a representative of one of two genera that form a sister lineage to the rest of the Orchidaceae, providing a reference for inferring the genome content and structure of the most recent common ancestor of all extant orchids and improving our understanding of their origins and evolution. In addition, we present transcriptome data for representatives of Vanilloideae, Cypripedioideae and Orchidoideae, and novel third-generation genome data for two species of Epidendroideae, covering all five orchid subfamilies. *A. shenzhenica* shows clear evidence of a whole-genome duplication, which is shared by all orchids and occurred shortly before their divergence. Comparisons between *A. shenzhenica* and other orchids and angiosperms also permitted the reconstruction of an ancestral orchid gene toolkit. We identify new gene families, gene family expansions and contractions, and changes within MADS-box gene classes, which control a diverse suite of developmental processes, during orchid evolution. This study sheds new light on the genetic mechanisms underpinning key orchid innovations, including the development of the labellum and gynostemium, pollinia, and seeds without endosperm, as well as the evolution of epiphytism; reveals relationships between the Orchidaceae subfamilies; and helps clarify the evolutionary history of orchids within the angiosperms.

**Supplementary information:**

The online version of this article (doi:10.1038/nature23897) contains supplementary material, which is available to authorized users.

## Abstract (web summary)

WebComparing the whole genome sequence of *Apostasia shenzhenica* with transcriptome and genome data from five orchid subfamilies permits the reconstruction of an ancestral gene toolkit, providing insight into orchid origins, evolution and diversification.

**Supplementary information:**

The online version of this article (doi:10.1038/nature23897) contains supplementary material, which is available to authorized users.

## Orchid origins

Around 10 per cent of flowering plant species are orchids, with a broad diversity in both morphology and lifestyle. *Apostasia* is one of the earliest-diverging genera of Orchidaceae. To study the evolution and diversity of Orchidaceae, Zhong-Jian Liu, Yves Van de Peer and colleagues sequenced the genome of *Apostasia shenzhenica*, a self-pollinating species found in southeast China. The authors also report improved genomes for two species of Epidendroideae, *Phalaenopsis equestris* and *Dendrobium catenatum*, as well as transcriptome analysis of representatives of subfamilies of Orchidaceae. Their analyses provide insights into orchid origins, genome evolution, adaptation and diversification.

**Supplementary information:**

The online version of this article (doi:10.1038/nature23897) contains supplementary material, which is available to authorized users.

## Main

**Extended Data Figure 1:**
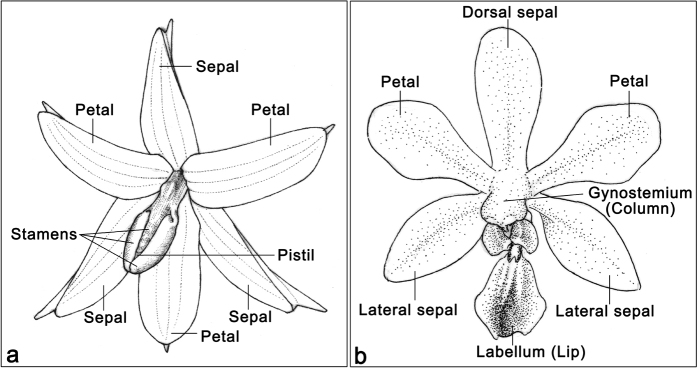
The morphology of orchid flowers. **a**, Illustration of an *Apostasia* flower. **b**, Illustration of a *Phalaenopsis* flower.

The Apostasioideae are a small subfamily of orchids that includes only two genera (*Apostasia* and *Neuwiedia*^2,5^), consisting of terrestrial species confined to the humid areas of southeast Asia, Japan, and northern Australia^6^. Although Apostasioideae share some synapomorphies with other orchids (for example, small seeds with a reduced embryo and a myco-heterotrophic protocorm stage), they possess several unique traits, the most conspicuous of which is their floral morphology^7^. *Apostasia* has a non-resupinate, solanum-type flower with anthers closely encircling the stigma (including postgenital fusion), a long ovary, and an actinomorphic perianth with an undifferentiated labellum. Three stamens (two of which are fertile) are basally fused to the style, forming a relatively simple gynostemium, and the anthers contain powdery pollen (grains not unified into pollinia). These characteristics (Extended Data Fig. 1a) differ from those of other Orchidaceae subfamilies, which have three sepals, three petals (of which one has specialized to form the labellum), and stamens and pistil fused into a more complex gynostemium (Extended Data Fig. 1b), but are similar to those of some species of Hypoxidaceae (a sister family to Orchidaceae, in the order Asparagales).

We sequenced the *A. shenzhenica* genome using a combination of different approaches; the total length of the final assembly was 349 Mb (see Methods and Supplementary Tables 1–4). We confidently annotated 21,841 protein-coding genes, of which 20,202 (92.50%) were supported by transcriptome data (Supplementary Fig. 1 and Supplementary Table 5). Using single-copy orthologues, we performed a BUSCO^8^ assessment that indicated that the completeness of the genome was 93.62%, suggesting that the *A. shenzhenica* genome assembly is of high quality (Supplementary Table 6). For comparative analyses, we also improved the quality of the previously published genome assemblies of the orchids *Phalaenopsis equestris*^9^ and *Dendrobium catenatum*^10^ (see Methods and Supplementary Tables 6 and 7).

**Figure 1:**
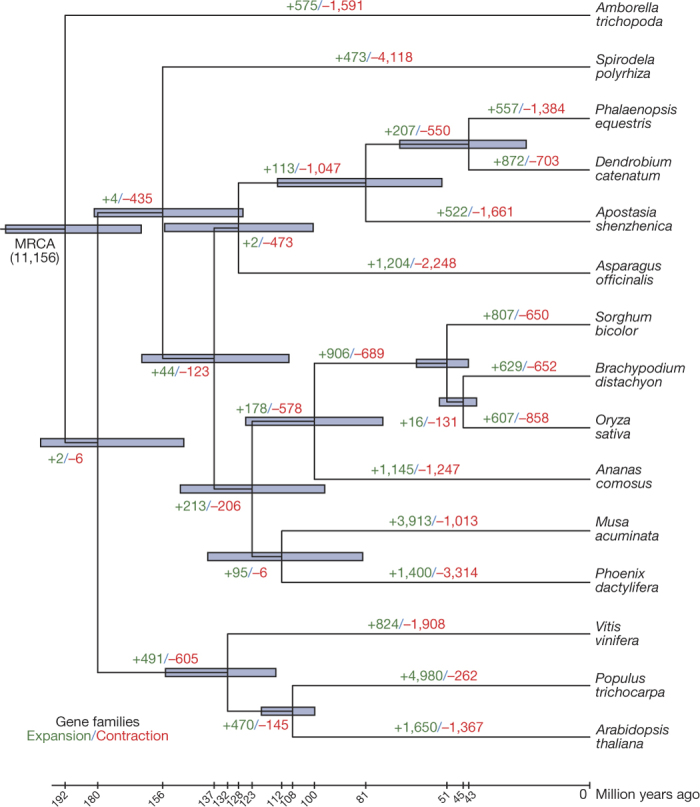
Phylogenetic tree showing divergence times and the evolution of gene family sizes. The phylogenetic tree shows the topology and divergence times for 15 plant species. As expected, as a member of the Apostasioideae, *A. shenzhenica* is sister to all other orchids. In general, the estimated orchid divergence times are in good agreement with recent broad scale orchid phylogenies^2,3^. Divergence times are indicated by light blue bars at the internodes; the range of these bars indicates the 95% confidence interval of the divergence time. Numbers at branches indicate the expansion and contraction of gene families (see Methods and Extended Data Fig. 2). MRCA, most recent common ancestor. The number in parentheses is the number of gene families in the MRCA as estimated by CAFÉ^11^. PowerPoint slide

**Extended Data Figure 2:**
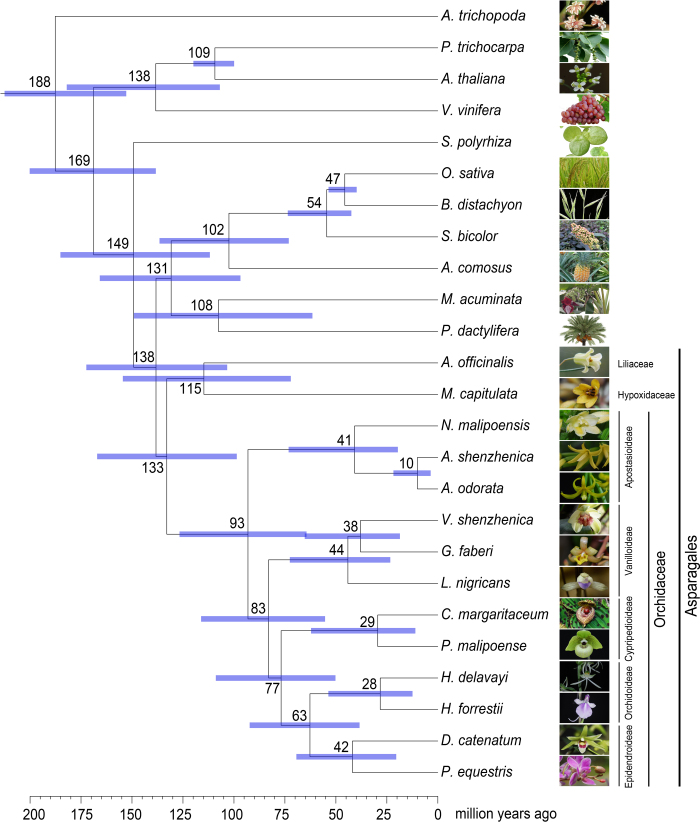
Phylogenetic tree showing the topology and divergence times for 15 genomes (*A. trichopoda*, *P. trichocarpa*, *A. thaliana*, *V. vinifera*, *Spirodela polyrhiza*, *O. sativa*, *Brachypodium distachyon*, *Sorghum bicolor*, *A. comosus*, *Musa acuminata*, *Phoenix dactylifera*, *A. officinalis*, *A. shenzhenica*, *P. equestris* and *D. catenatum*) and 10 transcriptomes (*Apostasia odorata*, *Cypripedium margaritaceum*, *Galeola faberi*, *Habenaria delavayi*, *Hemipilia forrestii*, *Lecanorchis nigricans*, *M. capitulata*, *Neuwiedia malipoensis*, *Paphiopedilum malipoense*, *Vanilla shenzhenica*). The unigenes of the transcriptomes of the 10 ‘transcriptome’ species were aligned to the 439 single-copy gene families of the 15 ‘genome’ species. One hundred and thirty-two single-copy gene families for the 25 species could be identified, and were used to construct a phylogenetic tree based on the PhyML software^61^ with the GTR+Γ model, while divergence times (indicated by light blue bars at the internodes) were predicted by MCMCTREE^62^. The range of the bars indicates the 95% confidence interval of the divergence times.

**Extended Data Figure 3:**
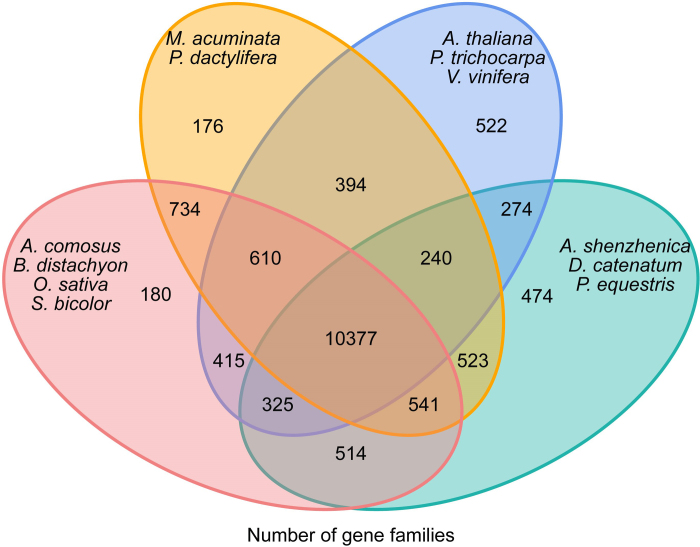
Venn diagram showing unique and shared gene families among members of Orchidaceae, dicots, and Poaceae, and *M. acuminata* and *P. dactylifera*. Numbers represent the number of gene families. Comparison of the 4 groups revealed 474 gene families unique to Orchidaceae and which exist in all 3 Orchidaceae species. If we consider lineage-specific gene families for each group (that is, gene families present in one or a few but not all species in a group), then there are 4,958 unique gene families for Orchidaceae, 7,503 for Poales, 4,494 for the dicots, and 1,560 for the group of *M. acuminata* and *P. dactylifera*.

We constructed a high-confidence phylogenetic tree and estimated the divergence times of 15 plant species using genes extracted from a total of 439 single-copy families (Fig. 1 and Extended Data Fig. 2). We undertook a computational analysis of gene family sizes (CAFÉ 2.2^11^) to study gene family expansion and contraction during the evolution of orchids and related species (Fig. 1 and Supplementary Note 1.1). By comparing 12 plant species, we found 474 gene families (Extended Data Fig. 3) that appeared unique to orchids (Supplementary Note 1.2). Gene Ontology and Kyoto Encyclopedia of Genes and Genomes (KEGG) enrichment analysis found these gene families to be specifically enriched in the terms ‘*O*-methyltransferase activity’, ‘cysteine-type peptidase activity’, ‘flavone and flavonol biosynthesis’ and ‘stilbenoid, diarylheptanoid and gingerol biosynthesis’ (Supplementary Note 1.2).

**Figure 2:**
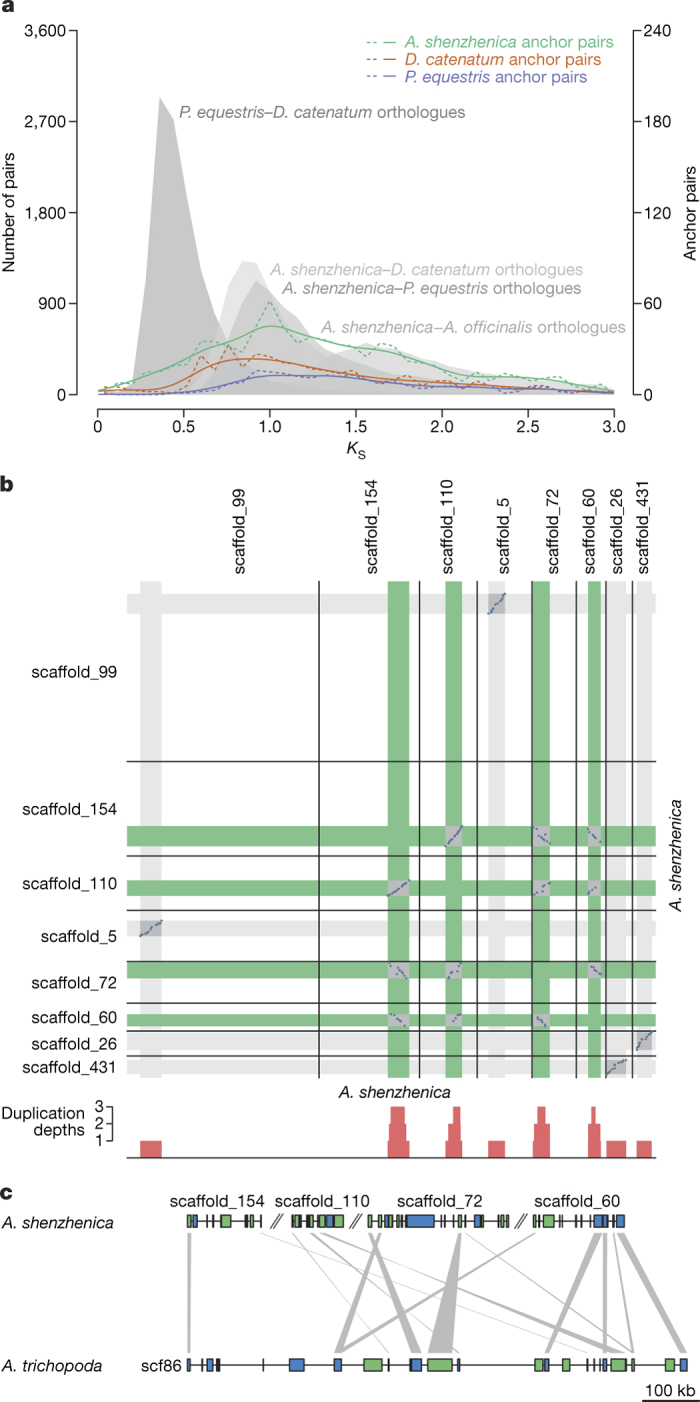
*K*_S_ and co-linearity analysis of the *A. shenzhenica* WGD. **a**, Distribution of *K*_S_ for the one-to-one *P. equestris*–*D. catenatum*, *A. shenzhenica*–*D. catenatum*, *A. shenzhenica*–*P. equestris* and *A. shenzhenica*–*A. officinalis* orthologues (filled grey curves and left-hand *y*-axis). Distribution of *K*_S_ for duplicated anchors found in co-linear regions of *A. shenzhenica* (green lines), *D. catenatum* (red lines) and *P. equestris* (blue lines). The filled grey curves and dashed coloured lines are actual data points from the distributions; the solid coloured lines are kernel density estimates (KDE) of the anchor-pair (duplicated genes found in co-linear regions) data scaled to match the corresponding dashed lines. All anchor-pair data are scaled up ×15 (right-hand *y*-axis) compared to the orthologue data. **b**, Syntenic dot plot of the self-comparison of *A. shenzhenica*. Only co-linear segments with at least 15 anchor pairs are shown. The sections on each scaffold with co-linear segments are shown in grey. The red bars below the dot plot illustrate the duplication depths (the number of connected co-linear segments overlapping at each position; see Methods). The co-linear regions in green indicate the four co-linear segments that have a common orthologous co-linear segment in *A. trichopoda* as shown in (**c**). **c**, Co-linear alignment of *A. shenzhenica* and *A. trichopoda*. The colours of genes in the alignment indicate gene orientation, with blue for forward strands and green for reverse strands. The grey links connect orthologues between *A. shenzhenica* and *A. trichopoda*. Scf86, scaffold00086 of the *A. trichopoda* genome (v1.0). PowerPoint slide

**Extended Data Figure 4:**
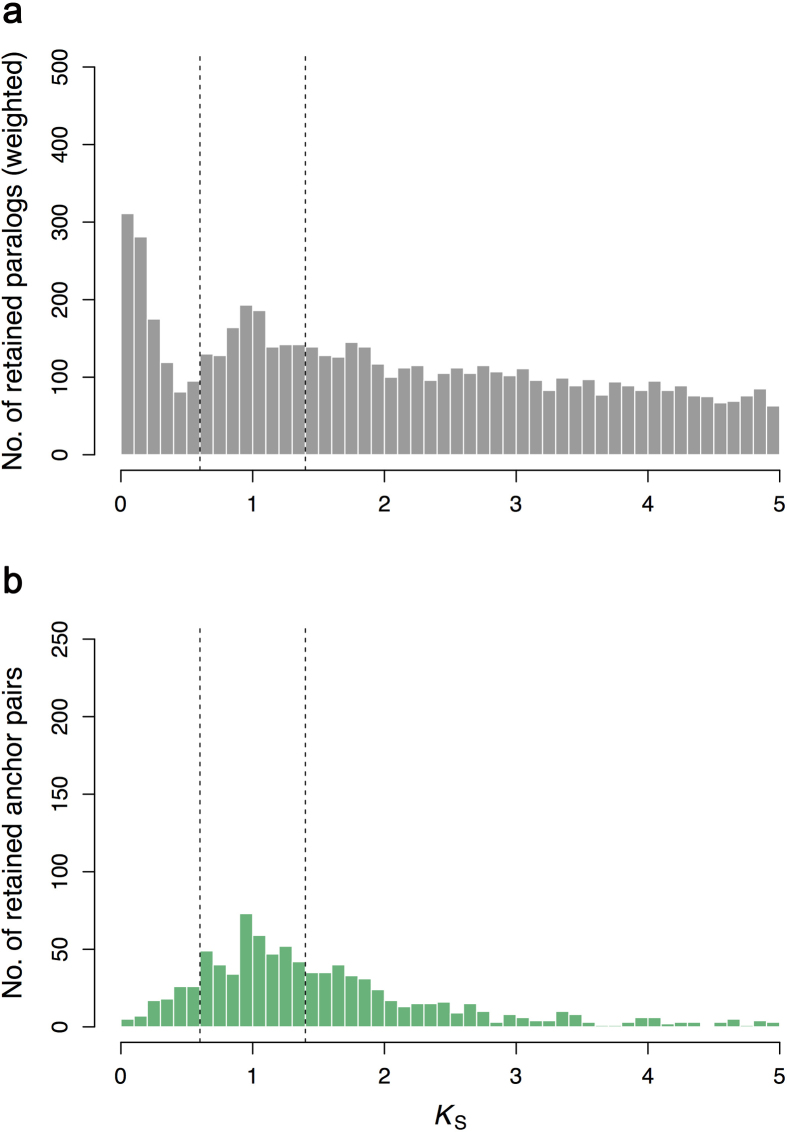
*A. shenzhenica K*_S_-based age distributions. **a**, Distribution of *K*_S_ for the whole *A. shenzhenica* paranome. **b**, Distribution of *K*_S_ for duplicated anchors found in co-linear regions as identified by i-ADHoRe. A WGD event is identified in both distributions with its peak centred on a *K*_S_ value of 1. The dashed lines indicate the *K*_S_ boundaries used to extract duplicate pairs for absolute phylogenomic dating of the WGD event (see Methods and Extended Data Fig. 5).

**Extended Data Figure 5:**
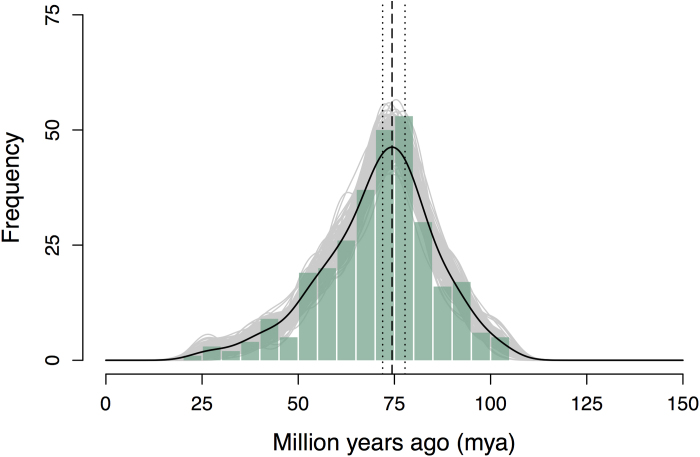
Absolute age of the *A. shenzhenica* WGD event. Absolute age distribution obtained by phylogenomic dating of *A. shenzhenica* paralogues. The solid black line represents the KDE of the dated paralogues, and the vertical dashed black line represents its peak at 74 Ma, which was used as the consensus WGD age estimate. The grey lines represent density estimates from 2,500 bootstrap replicates and the vertical black dotted lines represent the corresponding 90% confidence interval for the WGD age estimate, 72–78 Ma (see Methods). The histogram shows the raw distribution of dated paralogues.

Distributions of synonymous substitutions per synonymous site (*K*_S_) (see Supplementary Note 2.1) for paralogous *A. shenzhenica* genes showed a clear peak at *K*_S_ ≈ 1 (Extended Data Fig. 4). Similar peaks at *K*_S_ values of 0.7 to 1.1 were identified in 11 other orchids, covering all 5 orchid subfamilies (Supplementary Fig. 2). These peaks might reflect multiple independent whole-genome duplication (WGD) events across orchid sublineages or, more parsimoniously, a single WGD event shared by all (extant) orchids. Comparisons of orchid paralogue *K*_S_ distributions with *K*_S_ distributions of orthologues between orchid species, and between orchids and *Asparagus officinalis* (asparagus, Asparagaceae, a sister family to Orchidaceae in the order Asparagales) (Fig. 2a and Supplementary Note 2.1) indicated that the WGD signatures are not shared with non-orchid Asparagales. Absolute phylogenomic dating^12^ (Extended Data Fig. 5 and Supplementary Note 2.1) revealed that the WGDs and the earliest diversification of extant orchid lineages occurred relatively close together in time, supporting the possibility of a single WGD event in the most recent common ancestor of extant orchids.

**Figure 3:**
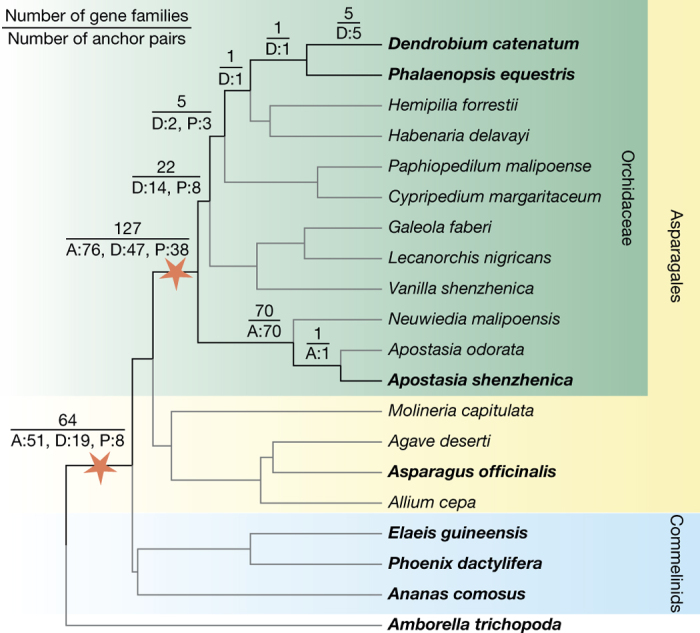
Phylogenomic analysis of orchid WGD events. The numbers on the branches of the species tree indicate the number of gene families with one or more anchor pairs from at least one of the three orchids with genomes that coalesced on the respective branch (top), as well as the individual contributions of anchor pairs from the three orchids (bottom; A, *A. shenzhenica*; D, *D. catenatum*; P, *P. equestris*). The two WGD events identified are depicted by stars. Species with published genomes are in bold. All the duplication events have bootstrap values over 80% (see Methods; for results for bootstrap values over 50% see Supplementary Fig. 15). PowerPoint slide

**Extended Data Figure 6:**
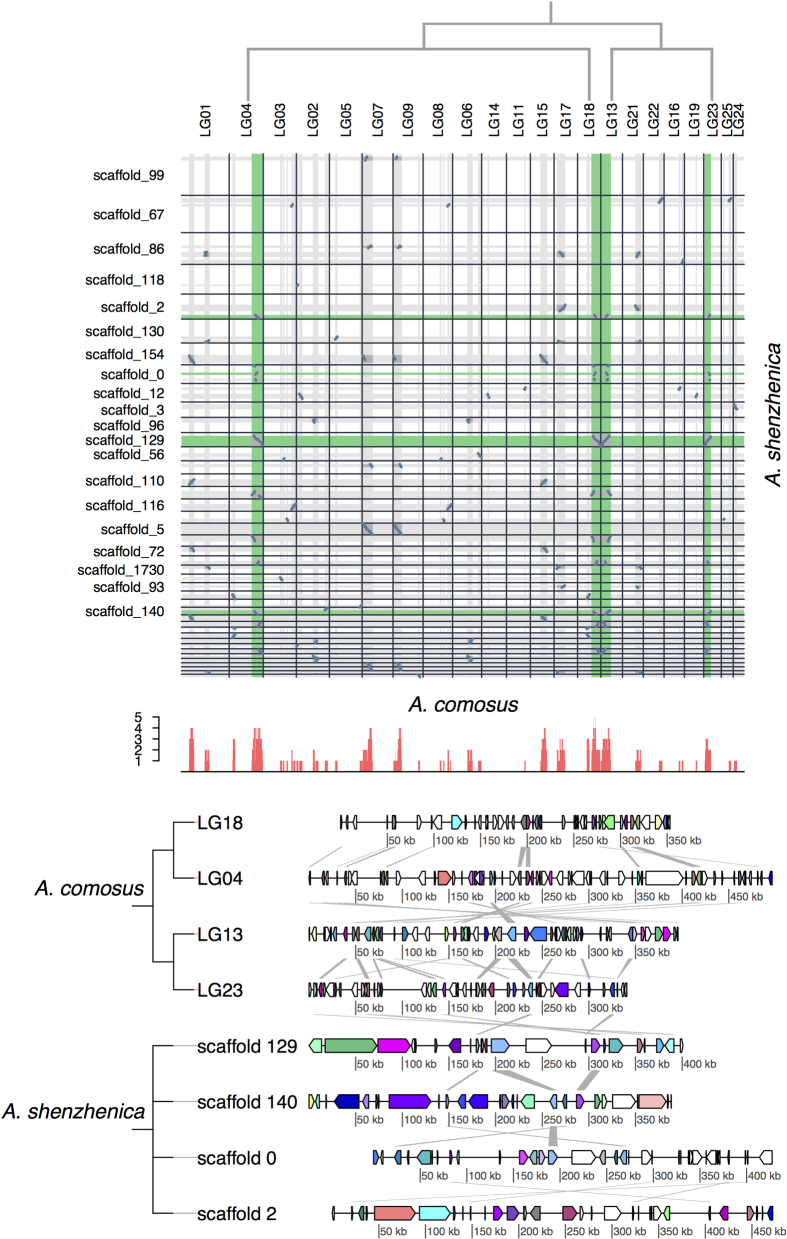
Co-linearity and synteny between *A. shenzhenica* and *A. comosus*. Only co-linear segments with at least 20 anchor pairs are shown. The sections on each scaffold with co-linear segments between *A. shenzhenica* and *A. comosus* are shown in grey. The red bars below the dot plot illustrate the duplication depths (the number of connected co-linear segments overlapping at each scaffold/chromosomal position; see Methods). Only connected co-linear segments with at least ten anchor pairs were used to calculate the duplication depths. The co-linear regions in green highlight the four co-linear segments in *A. shenzhenica* that correspond to a specific set of four co-linear segments in *A. comosus*, which originated from one of the seven ancestral pre-τ-WGD chromosomes in monocots (known as Anc6)^14^. The phylogenetic tree above the dot plot indicates how Anc6 evolved into (segments of) the current four chromosomes in *A. comosus* (the pair of paired LG18 and LG04, and LG13 and LG23; see Figure 2 in Ming *et al*.^14^) through two rounds of WGDs. Names of very small *A. shenzhenica* scaffolds are omitted for clarity. A part of the alignment of the co-linear segments between *A. shenzhenica* and *A. comosus* is shown below. The colours of genes in the alignment indicate anchor pairs with genes of the same colour being homologous. The grey links connect anchor pairs between the two closest segments.

Intragenomic co-linearity and synteny analysis identified two WGD events in *A. shenzhenica* (Fig. 2b, Supplementary Fig. 3 and Supplementary Note 2.2). Co-linearity and synteny analyses between *A. shenzhenica* and *Amborella trichopoda*, and between *A. shenzhenica* and *Vitis vinifera*, also support at least two WGDs in *A. shenzhenica* (Supplementary Figs 4 and 5); for example, four paralogous segments in *A. shenzhenica* corresponded to one orthologous region in *A. trichopoda* (Fig. 2c). Detailed genome comparisons of *A. shenzhenica* with *Ananas comosus* (pineapple) and *A. officinalis* revealed a specific 4:4 co-linearity pattern (Extended Data Fig. 6 and Supplementary Figs 6–8) that is consistent with the two monocot WGDs proposed for *A. comosus*, indicating that all three species possess a similar evolutionary history with regard to WGDs (Supplementary Note 2.2). Together, these patterns of co-linearity suggest that the older of the two WGDs evident in *A. shenzhenica* is likely to be shared with *A. comosus* and *A. officinalis* (representing the τ WGD^13,14^ shared by most monocots), and corroborate the idea that the younger WGD represents an independent event, specific to the Orchidaceae lineage. Analyses of gene trees that contained at least one paralogue pair from co-linear regions from one of the three orchid genomes placed the younger *A. shenzhenica* WGD and the *P. equestris* and *D. catenatum* WGDs on the orchid stem branch, and also provided additional evidence for the monocot τ WGD^13,14^ (Fig. 3 and Supplementary Note 2.3). We therefore find strong support for a WGD event shared by all extant orchids, which is likely to be only slightly older than their earliest divergence and might be correlated with orchid diversification. In addition, as observed for many other plant lineages, this orchid-specific WGD might be associated with the Cretaceous/Palaeogene boundary^15^.

**Figure 4:**
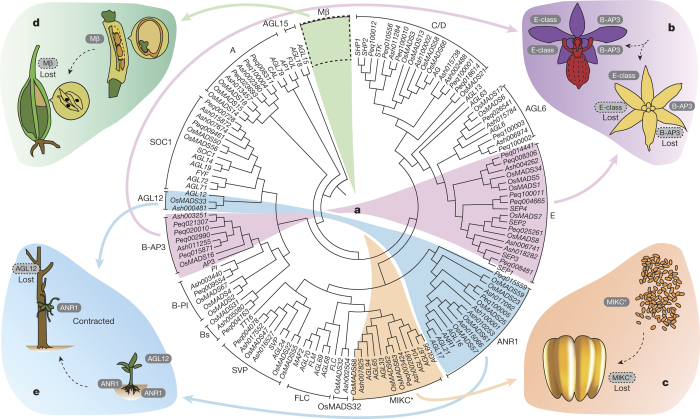
MADS-box genes involved in orchid morphological evolution. **a**, Phylogenetic analysis of MADS-box genes among *A. shenzhenica*, *P. equestris*, *O. sativa* and *Arabidopsis*. The B-AP3 and E-class, MIKC*, Mβ, and AGL12 and ANR1 subclades are marked by purple, orange, green and blue shading, respectively. **b**, *A. shenzhenica*, with fewer B-AP3 class and E class MADS-box genes, keeps an undifferentiated labellum and partially fused gynostemium, while *P. equestris*, with more B-AP3 class and E class MADS-box genes, develops the specialized labellum and column (in red). **c**, Loss of the P-subclade genes of MIKC* in *P. equestris* is likely to be related to the evolution of pollinia. **d**, The failed development of endosperm in orchids might be related to the missing type I Mβ MADS-box genes (Extended Data Fig. 9). **e**, *A. shenzhenica*, containing the *AGL12* gene and expanded ANR1 genes, is a terrestrial orchid, while epiphytic orchids, such as *P. equestris*, have lost the *AGL12* gene and some ANR1 genes. PowerPoint slide

**Extended Data Figure 7:**
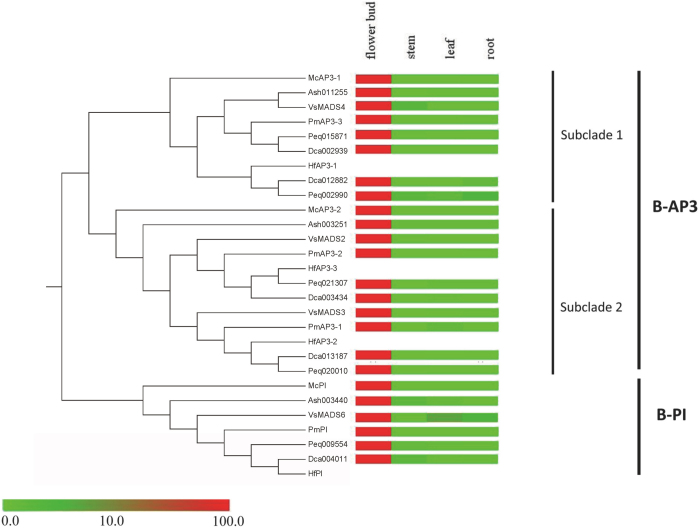
Phylogenetic and expression analysis of orchid B-AP3 genes. Ash, *A. shenzhenica*; Dca, *D. catenatum*; Hf, *H. forrestii*; Mc, *M. capitulata*; Peq, *P. equestris*; Pm, *P. malipoense*; Vs, *V. shenzhenica*. Expressions of B-class genes derived from *H. forrestii* are not shown, because only a flower sample was collected from *H. forrestii*. The expression levels (FPKM value) are represented by the colour bar.

*Apostasia* presents a number of characters that are plesiomorphic in orchids, such as an actinomorphic perianth with an undifferentiated labellum, a gynostemium with partially fused androecium and gynoecium, pollen that is not aggregated into pollinia, and underground roots for terrestrial growth^1,5,6,7^. The *A. shenzhenica* genome contains 36 putative functional MADS-box genes (Table 1, Supplementary Table 8 and Supplementary Fig. 9), 27 of which are type II MADS-box genes (Table 1). Two type II MADS-box classes appear to be reduced: *A. shenzhenica* seems to have fewer genes in the B-AP3 (two members) and E classes (three members) than *P. equestris* (four B-AP3 and six E-class members) and *D. catenatum* (four B-AP3 and five E-class members) (Fig. 4a). Previous studies have shown that expanded B-AP3 and E classes with members that have different expression patterns in floral organs are associated with the innovation of the unique labellum and gynostemium in orchids^9,16,17^, and that duplicated *B-AP3* genes are responsible for the modularization of the perianth of orchid flowers^18^. We identified *B-AP3* genes from the transcriptomes of species of each of the orchid subfamilies and the B-class MADS-box genes from the floral transcriptome data of *Molineria capitulata*, a member of Hypoxidaceae that possesses a flower with petaloid tepals and powdery pollen (similar to that found in *Apostasia*). We found one member in each of the two B-AP3 subclades for both *A. shenzhenica* and *M. capitulata*, but one or two members in each B-AP3 subclade for the other orchids (Extended Data Fig. 7). All these *B-AP3* genes are highly expressed in flower buds (Extended Data Fig. 7). These similarities suggest that the lower gene numbers in MADS-box B-AP3 and E classes in *Apostasia* represent an ancestral state, responsible for producing the plesiomorphic flower with an undifferentiated labellum and partially fused gynostemium. The B-AP3 and E classes may have expanded independently only in the non-apostasioid orchids or, alternatively, in the common ancestor of all extant orchids, possibly as a result of the shared orchid WGD, with subsequent loss of paralogous genes in *Apostasia* causing reversion to the ancestral state. The *B-AP3* gene tree topology and some evidence from co-linearity analysis of orchid *B-AP3* genes (Supplementary Fig. 10) suggest the latter. We hypothesize that differential paralogue retention and subsequent sub- and neo-functionalization of B-AP3 and E-class members resulted in the derived labellum found in other orchids (Fig. 4b).

**Table 1:** MADS-box genes in the *A. shenzhenica*, *P. equestris*, *D. catenatum*, *P. trichocarpa*, *A. thaliana* and *O. sativa* genomes

Category	*A. shenzhenica*		*P. equestris*		*D. catenatum*		*P. trichocarpa* ^*^		*A.thaliana* ^*^		*O. sativa* ^*^	
	Functional	Pseudo	Functional	Pseudo	Functional		Functional	Pseudo	Functional	Pseudo	Functional	Pseudo
Type II (Total)	27	4	29	1	35	11	64	3	47	5	48	1
MIKC^c^	25	3	28	1	32	9	55	2	43	4	47	1
MIKC^*^	2	1	1	0	3	2	2	0	2	0	1	0
Mδ	0	0	0	0	0	0	7	1	4	1	0	0
Type I (Total)	9	0	22	8	28	1	41	9	62	36	32	6
Mα	5	0	10	6	15	1	23	4	20	23	15	2
Mβ	0	0	0	0	0	0	12	5	17	5	9^†^	1
Mγ	4	0	12	2	13	0	6	0	21	8	8	3
Total	36	4	51	9	63	12	105	12	107	41	80	7

^*^Genes with stop codon in MADS-box domain were categorized as pseudogenes^29^.

^†^Nine MADS-box genes belonging to the M_β_ subgroup were identified^30^.

**Extended Data Figure 8:**
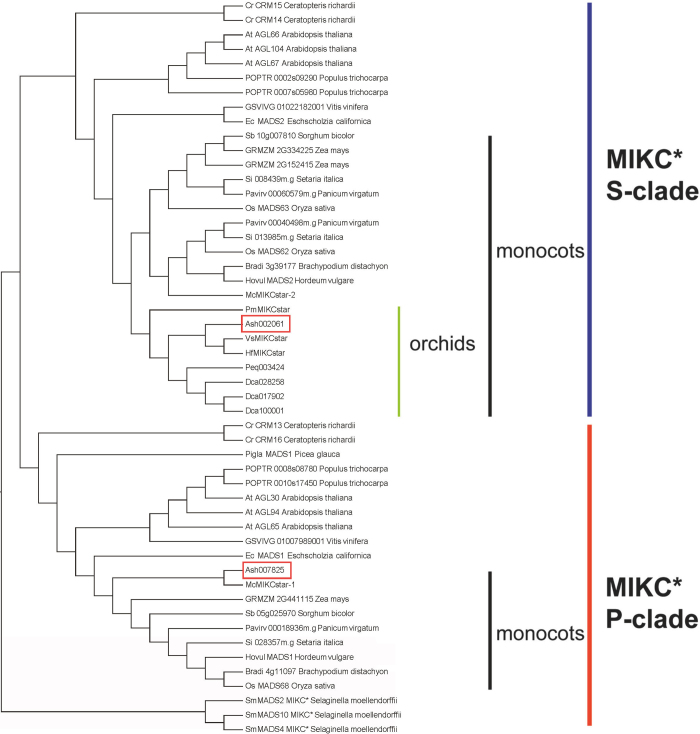
Phylogenetic tree of MIKC*-type genes. The red boxes indicate MADS-box genes from *A. shenzhenica.* Ash, *A. shenzhenica*; Dca, *D. catenatum*; Hf, *H. forrestii*; Mc, *M. capitulata*; Peq, *P. equestris*; Pm, *P. malipoense*; Vs, *V. shenzhenica*. MIKC* sequences of the other species were retrieved from GenBank based on Liu *et al*.^20^.

**Extended Data Figure 9:**
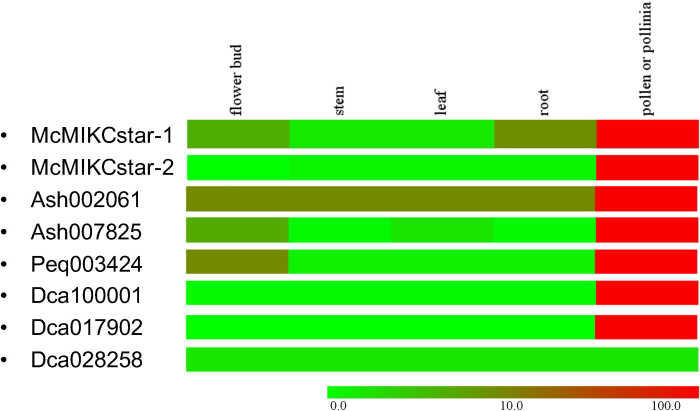
Expression patterns of MIKC* MADS-box genes. Ash, *A. shenzhenica*; Dca, *D. catenatum*; Mc, *M. capitulata*; Peq, *P. equestris*. The expression levels (FPKM value) are represented by the colour bar.

The packaging of pollen grains into a compact unit known as the pollinium, specialized for transfer as a unit by pollinating vectors, was a key innovation in the evolutionary history of Orchidaceae and may have played a role in promoting the tremendous radiation of the group^19^. In seed plants, the P- and S-subclades of MIKC*-type genes are major regulators of male gametophytic development^20,21^. The P-subclade, however, is absent in all orchids except *A. shenzhenica* (Extended Data Fig. 8). Gene expression analysis showed that, in orchids and *M. capitulata*, MIKC*-type genes are expressed in the pollinia or pollen, suggesting they play roles in its development (Extended Data Fig. 9). Although most orchids have a pollinium, *Apostasia* has scattered pollen, similar to *M. capitulata*, *Oryza sativa* (rice), and *Arabidopsis thaliana*. Therefore, we propose that the loss of the P-subclade members of MIKC*-type genes is related to the evolution of the pollinium (Fig. 4a, c and Supplementary Note 3).

**Extended Data Figure 10:**
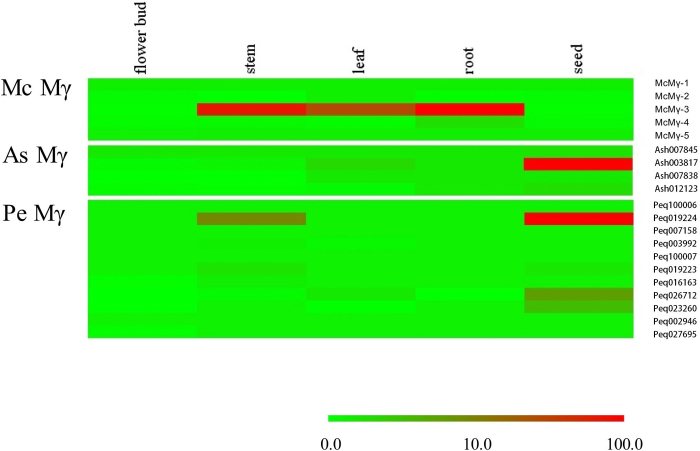
Expression of type I Mγ MADS-box genes in *M. capitulata*, *A. shenzhenica* and *P. equestris*. As, *A. shenzhenica*; Mc, *M. capitulata*; Pe, *P. equestris*. The expression levels (FPKM value) are represented by the colour bar.

The reduction of seed volume and content to an absolute minimum is a pivotal aspect of Orchidaceae evolution: in all orchid species, endosperm is absent from the seed. Type I MADS-box genes are important for the initiation of endosperm development^22^, and transcripts of type I Mα and Mγ MADS-box genes were found in developing seeds of *A. shenzhenica*, *P. equestris*, and *M. capitulata* (Extended Data Fig. 10 and Supplementary Fig. 11). Notably, the three orchid genomes do not contain any type I Mβ MADS-box genes (Fig. 4a and Supplementary Fig. 12), which are found in *Arabidopsis*, *Populus trichocarpa* (poplar), *O. sativa* (Table 1), and in *M. capitulata* (Supplementary Fig. 13). The lack of endosperm in orchids might therefore be related to the missing type I Mβ MADS-box genes (Fig. 4d).

Orchids are one of very few flowering plant lineages that have been able to successfully colonize epiphytic or lithophytic niches, clinging to trees or rocks and growing in dry conditions using crassulacean acid metabolism^2,9,10^. The roots of epiphytic orchids, such as *Phalaenopsis* and *Dendrobium*, are extremely specialized and differ from the roots of terrestrial orchids such as *Apostasia*. These aerial roots develop the velamen radicum, a spongy epidermis that traps the nutrient-rich flush during rainfall, representing an important adaptation of epiphytic orchids^23,24,25^. The *Arabidopsis AGL12* gene is involved in root cell differentiation^26^. *A. shenzhenica* contains one AGL12 clade gene, as do *Arabidopsis* and rice. In addition, we found transcripts similar to *AGL12* in *M. capitulata*. In both *A. shenzhenica* and *M. capitulata*, these genes are highly expressed in root tissue (Supplementary Fig. 14). Notably, we did not find similar genes in epiphytic orchids, suggesting that the loss of these gene(s) may be involved in losing the ability to develop true roots for terrestrial growth (Fig. 4e). *Utricularia gibba*, an asterid in the order Lamiales (only distantly related to the orchids) that lacks true roots, also lacks these AGL12 clade or similar genes^27^. The *Arabidopsis ANR1* gene is a key gene involved in regulating lateral root development in response to external nitrate supply^28^. We found that the MADS-box gene subfamily ANR1 is probably reduced in *P. equestris* (two members) and *D. catenatum* (three members), compared with four members in *A. shenzhenica* (Fig. 4a): this is consistent with no development of lateral (aerial) roots in epiphytic orchids.

In conclusion, the genome sequence of *A. shenzhenica*, an orchid belonging to a small clade that is sister to the rest of Orchidaceae, provides a reference for studying orchid evolution, revealing clear evidence of an ancient WGD shared by all orchids, facilitating reconstruction of the ancestral orchid gene toolkit, and providing insights into many orchid-specific features such as the development of the labellum and gynostemium, pollinia, and seeds without endosperm, as well as the evolution of epiphytism.

## Methods

No statistical methods were used to predetermine sample size.

### Sample preparation and sequencing

For genome sequencing, we collected leaves, stems, and flowers from wild *A. shenzhenica*, a self-pollinating species found in southeast China^4^ that has a karyotype of 2*N* = 2X = 68 with uniform small chromosomes (Supplementary Fig. 16). We extracted genomic DNA using a modified cetyltrimethylammonium bromide (CTAB) protocol. Sequencing libraries with insert sizes ranging from 180 bp to 20 kb (Supplementary Table 1) were constructed using a library construction kit (Illumina). These libraries were then sequenced using an Illumina HiSeq 2000 platform. The 80.02-Gb raw reads generated were filtered according to sequencing quality, the presence of adaptor contamination, and duplication. Only high-quality reads were used for genome assembly.

Total RNA was extracted from this study’s samples using the RNAprep Pure Plant Kit and genomic DNA contamination was removed using RNase-Free DNase I (both from Tiangen). The integrity of RNA was evaluated on a 1.0% agarose gel stained with ethidium bromide (EB), and its quality and quantity were assessed using a NanoPhotometer spectrophotometer (IMPLEN) and an Agilent 2100 Bioanalyzer (Agilent Technologies). As the RNA integrity number (RIN) was greater than 7.0 for all samples, they were used in cDNA library construction and Illumina sequencing, which was completed by Beijing Novogene Bioinformatics Technology Co., Ltd. The cDNA library was constructed using the NEBNext Ultra RNA Library Prep Kit for Illumina (NEB) and 3 μg RNA per sample, following the manufacturer’s recommendations. The PCR products obtained were purified (AMPure XP system) and library quality was assessed on the Agilent Bioanalyzer 2100 system. Library preparations were sequenced on an Illumina Hiseq 2000 platform, generating 100-bp paired-end reads.

### Genome size estimation and preliminary assembly

The genome size of species in Apostasioideae is between 0.38 pg and 5.96 pg^31^, which is relatively small compared to that of other subfamilies (ranging from 0.38 pg to 55.4 pg)^32^. To estimate the genome size of *A. shenzhenica*, we used reads from paired-end libraries to determine the distribution of *K*-mer values. According to the Lander–Waterman theory^33^, genome size can be determined by the total number of *K*-mers divided by the peak value of the *K*-mer distribution. Given only one peak in the *K*-mer distribution, we found that *A. shenzhenica* has no heterozygosity (Supplementary Fig. 17). With the peak at the expected *K*-mer depth and the formula genome size = total *K*-mer/expected *K-*mer depth, the size of the haploid genome was estimated to be 471.0 Mb (haploid). We used ALLPATHS-LG software^34^ and obtained a preliminary assembly of *A. shenzhenica* with a scaffold N50 size of 1.196 Mb and corresponding contig N50 size of 30.1 Kb.

### PacBio library construction and sequencing and filling gaps

The preliminary assembly of *A. shenzhenica* and the previous published genome assemblies of *P. equestris*^9^ and *D. catenatum*^10^ were improved using PacBio and 10X Genomics Linked-Reads.

Genomic DNA was isolated from the leaves of *A. shenzhenica*, *P. equestris* and *D. catenatum*. For a 20-kb insert size library, at least 20 μg of sheared DNA was required. SMRTbell template preparation involved DNA concentration, damage repair, end repair, ligation of hairpin adapters, and template purification, and used AMPure PB Magnetic Beads. Finally, the sequencing primer was annealed and sequencing polymerase was bound to SMRTbell template. The instructions specified as calculated by the RS Remote software were followed. We carried out 20-kb single-molecule real-time DNA sequencing by PacBio and sequenced the DNA library on the PacBio RS II platform, yielding about 5.44 Gb (*A. shenzhenica*), 10.54 Gb (*P. equestris*) and 11.06 Gb (*D. catenatum*) PacBio data (read quality ≥ 0.80, mean read length of *A. shenzhenica* ≥ 7 Kb, of *P. equestris* and *D. catenatum* ≥ 10 Kb) (Supplementary Table 2).

We used PBjelly software^35^ to fill gaps with PacBio data. The options were “<blasr>-minMatch 8 -sdpTupleSize 8 -minPctIdentity 75 -bestn 1 -nCandidates 10 -maxScore -500 -nproc 10 -noSplitSubreads</blasr>” for the protocol.xml file. Then, we used Pilon^36^ with default settings to correct assembled errors. For the input BAM file, we used BWA to align all the Illumina short reads to the assembly and SAMTOOLS to sort and index the BAM file.

### 10X Genomics library construction, sequencing, and extending scaffolds

DNA sample preparation, indexing, and barcoding were done using the GemCode Instrument from 10X Genomics. About 0.7 ng input DNA with 50 kb length was used for GEM reaction procedure during PCR, and 16-bp barcodes were introduced into droplets. Then, the droplets were fractured following the purifying of the intermediate DNA library. Next, we sheared DNA into 500 bp for constructing libraries, which were finally sequenced on the Illumina HiseqXTen^37^ (Supplementary Table 3).

We used BWA mem to align the 10X Genomics data to the filled gaps assembly using default settings. Then, we used fragScaff^38^ for scaffolding. The options were as follows: *A. shenzhenica* (stages1 “-m 3000 -q 30”; stages2 “-C 2”; stages3 “-j 1.25 -u 2”), *D. catenatum* (stages1 “-m 3000 -q 30”; stages2 “-C 1”; stages3 “-j 2 -u 2”) and *P. equestris* (stages1 “-m 3000 -q 30”; stages2 “-C 1”; stages3 “-j 2 -u 2”)^39^.

The total length of the final assembly for *A. shenzhenica* was 349 Mb with a scaffold N50 size of 3.029 Mb and corresponding contig N50 size of 80.1 Kb. (Supplementary Table 4). For the two previously published orchid genomes of *P. equestris* and *D. catenatum*, the scaffold N50 size as well as the completeness (see below) improved considerably: for *P. equestris*, the scaffold N50 size increased from 359.12 Kb^9^ to 1.217 Mb and the corresponding contig N50 size from 20.56 Kb^9^ to 45.79 Kb, while for *D. catenatum* the scaffold N50 size increased from 391.46 Kb^10^ to 1.055 Mb, and the corresponding contig N50 size from 33.1 Kb^10^ up to 51.7 Kb (Supplementary Table 7).

### Repeat prediction

A total of 146.65 Mb of repetitive elements occupying more than 42.05% of the *A. shenzhenica* genome were annotated using a combination of structural information and homology prediction^10^. Retrotransposable elements, known to be the dominant form of repeats in angiosperm genomes, constituted a large part of the *A. shenzhenica* genome and included the most abundant subtypes, such as LTR/Copia (4.97%), LTR/Gypsy (11.84%), LINE/L1 (2.78%) and LINE/RTE-BovB (9.32%), among others. In addition, the percentage of *de novo* predicted repeats was notably larger than that obtained for homologous repeats based on Repbase^40^, indicating that *A. shenzhenica* has multiple unique repeats compared with other sequenced plant species (Supplementary Table 9).

### Gene and non-coding RNA prediction

MAKER^41^ was used to generate a consensus gene set based on *de novo* predictions from AUGUSTUS^42^ and GlimmerHMM^43^, homology annotation with the universal single-copy genes from CEGMA^44^ and the genes from *Arabidopsis* (TAIR10) and another four sequenced monocots (*O. sativa*, *P. equestris*, *S. bicolor* and *Zea mays*) using exonerate^45^, and RNA-seq prediction by Cufflinks^46^ and Tophat^47^. These results were integrated into a final set of protein-coding genes for annotation (Supplementary Table 5). Using the same annotation pipeline as for *A. shenzhenica*, 29,545 and 29,257 protein-coding genes were predicted for *P. equestris* and *D. catenatum*, respectively (Supplementary Table 7). *A. shenzhenica* was found to have a greater average gene length (here we considered the start and stop codons as the two boundaries for a gene) than most other sequenced plants, but this length was similar to that of *P. equestris* and *D. catenatum* (Supplementary Fig. 18 and Supplementary Table 10), in both of which this is due to a long average intron length^9,10^.

We then generated functional assignments of the *A. shenzhenica* genes with BLAST (version 2.2.28+) by aligning their protein-coding regions to sequences in public protein databases, including KEGG (59.3)^48^, SwissProt (release 2013_06)^49^, TrEMBL (release 2013_06)^50^ and NCBI non-redundant protein database (20150617), and InterProScan (v5.11-51.0)^51^ was also used to provide functional annotation (Supplementary Table 11). We were able to generate functional assignments for 84.2% of the *A. shenzhenica* genes from at least one of the public protein databases (Supplementary Table 11).

The tRNA genes were searched by tRNAscan-SE^52^. For rRNA identification, we downloaded the *Arabidopsis* rRNA sequences from NCBI and aligned them with the *A. shenzhenica* genome to identify possible rRNAs. Additionally, other types of non-coding RNAs, including miRNA and snRNA, were identified by using INFERNAL^53^ to search from the Rfam database. In the end, we identified 43 microRNAs, 203 transfer RNAs, 452 ribosomal RNAs and 93 small nuclear RNAs in the *A. shenzhenica* genome (Supplementary Table 12).

### Transcriptome assembly

Before assembly, we got high-quality reads by removing adaptor sequences and filtered low-quality reads by using TRIMMOMATIC^54^ from raw reads with parameters: ILLUMINACLIP:path/adaptor:2:30:10 LEADING:5 TRAILING:5 SLIDINGWINDOW:4:15 MINLEN:36. The resulting high-quality reads were *de novo* assembled and annotated with the TRINITY program^55^. The commands and parameters used for running TRINITY were as follows: Trinity –seqType fq –JM 200G –left sample_1.fq –right sample_2.fq –normalize_by_read_set –CPU 32 –output sample –min_kmer_cov 2. Protein sequences and coding sequences of transcripts were predicted using TransDecoder (http://transdecoder.github.io), a software tool that identifies likely coding sequences from transcript sequences and compares the translated coding sequences with the PFAM domain database^55^. For genes with more than one transcript, the longest one was used to calculate transcript abundance and coverage. Transcript abundance level was normalized using the fragments per kilobase per million mapped reads (FPKM) method, and FPKM values were computed as proposed by Mortazavi *et al*.^56^.

Transcriptomes of *Agave deserti*^57^ and *Allium cepa*^58^ were downloaded from Dryad (h5t68) and NCBI (PRJNA175446), respectively. We removed the redundant unigenes in *A. cepa* by CD-HIT-EST with 99% identity and used TransDecoder to predict proteins with default parameters.

We carried out BLASTP (*E* value <1 × 10^−3^) to search the best hits for the proteins predicted in the transcriptomes against a customized database, built with proteins from the genomes of *A. shenzhenica*, *P. equestris*^9^, *D. catenatum*^10^, and *A. officinalis* (GenBank accession number GCF_001876935.1) as well as public databases, such as NCBI Plant RefSeq (release 80), Ensembl (release 77), Ensembl Metazoa (release 24), Ensembl Fungi (release 24), and Ensembl Protists (release 24). Only plant-homologous proteins were retained in the transcriptomes to eliminate the effects of genes derived from commensal organisms, laboratory contaminants, and artefacts resulting from incorrect assembly (Supplementary Table 13).

### Gene family identification

We downloaded genome and annotation data of *A. trichopoda* (http://amborella.huck.psu.edu, version1.0), *A. comosus* (GenBank accession number GCF_001540865.1), *A. thaliana* (TAIR 10), *A. officinalis* (GenBank accession number GCF_001876935.1), *B. distachyon* (purple false brome; Phytozome v9.0), *M. acuminata* (http://ensemblgenomes.org, release-21), *O. sativa* (Nipponbare, IRGSP-1.0), *P. dactylifera* (http://qatar-weill.cornell.edu/research/datepalmGenome), *P. trichocarpa* (http://ensemblgenomes.org, release-21), *S. bicolor* (sorghum; Phytozome v9.0), *S. polyrhiza* (common duckweed; http://www.spirodelagenome.org), and *V. vinifera* (Phytozome v9.0). We chose the longest transcript to represent each gene and removed gene models with open reading frames shorter than 150 bp. Gene family clustering was performed using OrthoMCL^59^ based on the set of 21,841 predicted genes of *A. shenzhenica* and the protein sets of the above ten other monocots, three dicots and the outgroup *A. trichopoda.* This analysis yielded 11,995 gene families in *A. shenzhenica* containing 18,268 predicted genes (83.6% of the total genes identified; orthologous genes in the 15 sequenced plant species are shown in Supplementary Fig. 19 and Supplementary Table 14) (see also Supplementary Note 1).

### Phylogenetic tree construction and phylogenomic dating

We constructed a phylogenetic tree based on a concatenated sequence alignment of 439 single-copy gene families from *A. shenzhenica* and the 14 other plant species using MrBayes^60^ software with GTR+Γ model (Fig. 1). For the phylogenetic analysis incorporating ten additional transcriptome species (Extended Data Fig. 2), we first picked up the genes of *A. shenzhenica*, *D. catenatum*, and *P. equestris* in the single-copy gene families as seed genes, and then made a BLASTP alignment between the transcriptome unigenes and the seed sequences. For one single-copy family, if the three seed genes all had the identical best-hit to a unigene, this gene was identified as the orthologous gene to the gene family. With this method we found 132 single-copy gene families of the total 25 species, then constructed the phylogenetic tree based on a concatenated sequence alignment of them using PhyML^61^ with GTR+Γ model. Divergence times were estimated by PAML MCMCTREE^62^. The Markov chain Monte Carlo (MCMC) process was run for 1,500,000 iterations with a sample frequency of 150 after a burn-in of 500,000 iterations. Other parameters used the default settings of MCMCTREE. Two independent runs were performed to check convergence. The following constraints were used for time calibrations: (i) the *O. sativa* and *B. distachyon* divergence time (40–54 million years ago (Ma))^63^; (ii) the *P. trichocarpa* and *A. thaliana* divergence time (100–120 Ma)^64^; (iii) the monocot and eudicot divergence time with a lower boundary of 130 Ma^65^; and (iv) 200 Ma as the upper boundary for the earliest-diverging angiosperms^66^.

### Identification of WGD events in *A. shenzhenica* and phylogenomic analyses

*K*_S_-based age distributions were constructed as previously described^67^. In brief, the paranome was constructed by performing an all-against-all protein sequence similarity search using BLASTP with an *E* value cutoff of 1 × 10^−10^, after which gene families were built with the mclblastline pipeline (v10-201) (http://micans.org/mcl)^68^. Each gene family was aligned using MUSCLE (v3.8.31)^69^, and *K*_S_ estimates for all pairwise comparisons within a gene family were obtained through maximum likelihood estimation using the CODEML program^70^ of the PAML package (v4.4c)^62^. Gene families were then subdivided into subfamilies for which *K*_S_ estimates between members did not exceed a value of 5. To correct for the redundancy of *K*_S_ values (a gene family of *n* members produces *n*(*n*−1)/2 pairwise *K*_S_ estimates for *n*−1 retained duplication events), a phylogenetic tree was constructed for each subfamily using PhyML^61^ under default settings. For each duplication node in the resulting phylogenetic tree, all *m K*_S_ estimates between the two child clades were added to the *K*_S_ distribution with a weight of 1/*m* (where *m* is the number of *K*_S_ estimates for a duplication event), so that the weights of all *K*_S_ estimates for a single duplication event summed to one. The resulting age distribution of the *A. shenzhenica* paranome is shown in Extended Data Fig. 4a.

Absolute dating of the identified WGD event in *A. shenzhenica* was performed as previously described^9,12^. In brief, paralogous gene pairs located in duplicated segments (anchors) and duplicated pairs lying under the WGD peak (peak-based duplicates) were collected for phylogenetic dating. Anchors, assumed to correspond to the most recent WGD event, were detected using i-ADHoRe (v3.0)^71,72^. Their *K*_S_ distribution is shown in Extended Data Fig. 4b. The identified anchors confirmed the presence of a WGD peak near a *K*_S_ value of 1 (the long tail and additional peaks in the anchor pair distribution are most likely due to small saturation effects^67^ and the remnants of older WGD events in the monocot lineage, such as the τ WGD^13,14^). We selected anchor pairs and peak-based duplicates present under the WGD peak and with *K*_S_ values between 0.6 and 1.4 (dashed lines in Extended Data Fig. 4a, b) for absolute dating. For each WGD paralogous pair, an orthogroup was created that included the two paralogues plus several orthologues from other plant species as identified by InParanoid (v4.1)^73^ using a broad taxonomic sampling: one representative orthologue from the order Cucurbitales, one from the Rosales, two from the Fabales, one from the Malpighiales, two from the Brassicales, one from the Malvales, one from the Solanales, two from the Poaceae (Poales), one from *A. comosus*^14^ (Bromeliaceae, Poales), one from either *M. acuminata*^74^ (Zingiberales) or *P. dactylifera*^75^ (Arecales), and one orthologue from the Alismatales, either from *S. polyrhiza*^76^ or *Zostera marina*^77^. In total, 85 orthogroups based on anchors and 230 orthogroups based on peak-based duplicates were collected. The node joining the two *A. shenzhenica* WGD paralogues was then dated using the BEAST v1.7 package^78^ under an uncorrelated relaxed clock model and an LG+G (four rate categories) evolutionary model. A starting tree with branch lengths satisfying all fossil prior constraints was created according to the consensus APGIV phylogeny^79^. Fossil calibrations were implemented using log-normal calibration priors on the following nodes: the node uniting the Malvidae based on the fossil *Dressiantha bicarpellata*^80^ with prior offset = 82.8, mean = 3.8528, and s.d. = 0.5^81^; the node uniting the Fabidae based on the fossil *Paleoclusia chevalieri*^82^ with prior offset = 82.8, mean = 3.9314, and s.d. = 0.5^83^; the node uniting the *A. shenzhenica* WGD paralogues with the other non-Alismatalean monocots based on fossil *Liliacidites*^84^ with prior offset = 93.0, mean = 3.5458, and s.d. = 0.5^85^; and the root with prior offset = 124, mean = 4.0786, and s.d. = 0.5^86^. The offsets of these calibrations represent hard minimum boundaries, and their means represent locations for their respective peak mass probabilities in accordance with some recent and most taxonomically complete dating studies available for these specific clades^87^. A run without data was performed to ensure proper placement of the marginal calibration prior distributions^88^. The MCMC for each orthogroup was run for 10 million generations with sampling every 1,000 generations, resulting in a sample size of 10,000. The resulting trace files of all orthogroups were evaluated manually using Tracer v1.5^78^ with a burn-in of 1,000 samples to ensure proper convergence (minimum ESS for all statistics was at least 200). In total, 303 orthogroups were accepted, and all age estimates for the node uniting the WGD paralogous pairs were then grouped into one absolute age distribution (Extended Data Fig. 5; too few anchor pairs were available to evaluate them separately from the peak-based duplicates), for which KDE and a bootstrapping procedure were used to find the peak consensus WGD age estimate and its 90% confidence interval boundaries, respectively. More detailed methods are available in Vanneste *et al*.^12^.

To compare the relative timing of speciations and WGD event(s) in orchids based on *K*_S_ distributions, we first identified 839 anchors from *D. catenatum* and 355 anchors from *P. equestris* using i-ADHoRe 3.0 and calculated their *K*_S_ as described above. Identification of orthologues between *A. shenzhenica* and *A. officinalis*, *A. shenzhenica* and *P. equestris*, *A. shenzhenica* and *D. catenatum*, and *P. equestris* and *D. catenatum* was performed first by reciprocal BLASTP with *E* value <1 × 10^−5^ for proteins from the three orchids and asparagus, followed by sorting BLAST hits by bit-scores and *E* values. Reciprocal best hits in the four comparisons were selected as orthologues. In this way, we identified 9,142 orthologues between *A. shenzhenica* and *A. officinalis*, 10,699 orthologues between *A.shenzhenica* and *P. equestris*, 11,386 orthologues between *A. shenzhenica* and *D. catenatum*, and 13,139 orthologues between *P. equestris* and *D. catenatum*. For each pair of orthologues, ClustalW^89^ alignment was carried out to perform sequence alignment using the parameter for amino acids recommended by Hall^90^. PAL2NAL^91^ was then used to back-translate aligned protein sequences into codon sequences and to remove any gaps in the alignment. Estimates of *K*_S_ values were obtained from CODEML in PAML using the Goldman-Yang model with codon frequencies estimated by the F3 × 4 model.

We performed pairwise co-linearity analysis within *A. shenzhenica* and between *A. shenzhenica* and *A. officinalis*, *A. comosus*, *V. vinifera*, and *A. trichopoda*. Homologous pairs of *A. shenzhenica* and the above species were identified by all-against-all BLASTP (*E* value <1 × 10^−5^), followed by the removal of weak matches by applying a *c*-score of 0.5 (indicating their BLASTP bit-scores were below 50% of the bit-scores of the best matches)^92^. Then, i-ADHoRe 3.0 was used to identify co-linear segments with parameters as described above except using ‘level_2_only = FALSE’, enabling the functionality to detect highly degenerated co-linear segments resulting from more ancient large-scale duplications (this is achieved by recursively building genomic profiles based on relatively recent co-linear segments). All co-linear dot plots were drawn by selecting co-linear segments according to a specified required number of anchor pairs (given in the figure legend of each of the dot plots). For the comparisons between *A. shenzhenica* and the chromosome-level assembled genomes (*A. officinalis*, *A. comosus*, and *V. vinifera*) we retained co-linear segments with at least ten anchor pairs (Extended Data Fig. 6 and Supplementary Figs 5, 7, 8). For the comparisons with fragmented genomes, like *A. trichopoda*, and the self-comparison of *A. shenzhenica*, we kept co-linear segments with five anchor pairs (Fig. 2b and Supplementary Figs 3, 4). The start and end boundaries of selected co-linear segments were used to define broader regions containing such segments on the chromosomes or scaffolds by further connecting co-linear segments if they overlapped with each other. Then, duplication depths, that is, the number of connected co-linear segments overlapping at each position of a broader region, were illustrated in the margins of the plots by mapping the connected co-linear segments over each other. The number of anchors required in the co-linear segments could affect the duplication depth in such a way that increasing the number of anchors required tends to remove co-linear segments originating from more ancient WGD(s) due to increased gene loss.

To identify the duplication events that resulted in the 1,488 anchor pairs in *A. shenzhenica*, the 839 anchor pairs in *D. catenatum*, and the 355 anchor pairs in *P. equestris*, we performed phylogenomic analyses employing protein-coding genes from 20 species, including 12 orchids across all five subfamilies of Orchidaceae (the three orchids with genomes (*A. shenzhenica*, *D. catenatum* and *P. equestris*) plus nine orchid transcriptomes (Supplementary Table 13)), four non-orchid Asparagales (*A. officinalis* (genome), *M. capitulata* (Supplementary Table 13), *A. deserti*^57^ and *A. cepa*^58^), three commelinid monocots (*Elaeis guineensis*, *P. dactylifera*, and *A. comosus*), and *A. trichopoda*. OrthoMCL (v2.0.9)^59^ was used with default parameters to identify gene families based on sequence similarities resulting from an all-against-all BLASTP with *E* value <1 × 10^−5^. Then, 1,101 of the 2,582 anchor pairs with *K*_S_ values greater than five were removed. If the remaining anchors fell into different gene families, indicating incorrect assignment of gene families by OrthoMCL, we merged the corresponding gene families. In this way, we obtained 32,217 multi-gene gene families. Next, phylogenetic trees were constructed for the subset of 777 gene families with no more than 300 genes that had at least one pair of anchors and one gene from *A. trichopoda*. Multiple sequence alignments were produced by MUSCLE (v3.8.31) using default parameters. These were trimmed by trimAl (v1.4)^93^ to remove low-quality regions based on a heuristic approach (-automated1) that depends on a distribution of residue similarities inferred from the alignments for each gene family. RAxML (v8.2.0)^94^ was then used with the GTR+Γ model to estimate a maximum likelihood tree starting with 200 rapid bootstraps followed by maximum likelihood optimizations on every fifth bootstrap tree. Gene trees were rooted based on genes from *A. trichopoda* if these formed a monophyletic group in the tree; otherwise, mid-point rooting was applied. The timing of the duplication event for each anchor pair relative to the lineage divergence events was then inferred using the following approach (Supplementary Fig. 20): we first mapped internodes from a gene tree to the species phylogeny according to the common ancestor of the genes in the gene tree. Each internode of the gene tree was then defined as either a duplication node, a speciation node, or a ‘dubious’ node. A duplication node is a node that shares at least one pair of paralogues, a speciation node is a node that has no paralogues and is consistent with divergence in the species phylogeny, and a ‘dubious’ node is a node that has no paralogues and is inconsistent with divergence in the species phylogeny. Then, if a pair of anchors coalesced to a duplication node, we traced back its parental node(s) until we reached a speciation node in the gene tree. In this way, we circumscribed the duplication event as between these two nodes with the duplication node as the lower bound and the speciation node as the upper bound on the species tree. If the two nodes were directly connected by a single branch on the species tree, the duplication was thus considered to have occurred on the branch. To reduce biased estimations, we used the bootstrap value on the branch leading to the common ancestral node of an anchor pair as support for a duplication event. In total, 628 anchor pairs in 493 gene families coalesced as duplication events on the species phylogeny, and duplication events from 318 anchor pairs in 262 gene families (or from 448 anchor pairs in 367 gene families) had bootstrap values greater than or equal to 80% (or 50%).

### Evolution and expression analysis of orchid MADS box genes

We identified candidates of MADS-box genes by searching the InterProScan^51^ result of all the predicted *A. shenzhenica* proteins. The candidates of MADS-box genes were further determined by SMART^95^, which identified MADS-box domains comprised by 60 amino acids. The protein-sequence set of the MADS-box gene candidates was BLAST against the assembled *A. shenzhenica* transcriptomes with the TBLASTN program. The matched transcript sequences were then assembled with the candidates of MADS-box genes using Sequencher v5.1 (Gene Codes Corp.) and the exon structure of the final MADS-box genes was manually edited (Supplementary Data 1). In the end, we aligned all the identified MADS-box genes using the ClustalW program^89^. An unrooted neighbour-joining phylogenetic tree was constructed in MEGA5^96^ with default parameters.

### Transcriptomic analysis of other orchids

In addition, 53 more transcriptomes derived from 9 more taxa and 8 tissues (flower bud, anther, pollinium, shoot, stem, leaf, aerial root and root) (Supplementary Table 13) were sampled to investigate the roles of the genes that may be important for the evolution of orchid traits. The gene expression levels were indicated by FPKM on the longest assembled transcript.

### Data availability

Genome sequences and whole-genome assembly of *A. shenzhenica* and whole transcriptomes have been submitted to the National Center for Biotechnology Information (NCBI) database under BioProject PRJNA310678; the remaining transcriptomes used in this study can be found in the previously available BioProjects PRJNA288388, PRJNA304321, and PRJNA348403; the raw data and the updated whole-genome assembly of *P. equestris* have been submitted to NCBI under BioProject PRJNA389183; and the raw data and the updated whole-genome assembly of *D. catenatum* have been renewed under the already existing BioProject PRJNA262478. All other data are available from the corresponding authors upon reasonable request.

This work is licensed under a Creative Commons Attribution 4.0 International (CC BY 4.0) licence. The images or other third party material in this article are included in the article’s Creative Commons licence, unless indicated otherwise in the credit line; if the material is not included under the Creative Commons licence, users will need to obtain permission from the licence holder to reproduce the material. To view a copy of this licence, visit http://creativecommons.org/licenses/by/4.0/.

## Supplementary information

Supplementary Information This file contains Supplementary Notes 1-3, Supplementary Figures 1-27, Supplementary Tables 1-23, Supplementary Data and additional references. (PDF 7375 kb)

## Data Availability

BioProject
PRJNA262478
PRJNA310678
PRJNA389183

## PowerPoint slides

PowerPoint slide for Fig. 1
PowerPoint slide for Fig. 2
PowerPoint slide for Fig. 3
PowerPoint slide for Fig. 4
