# Author Correction: The *Apostasia* genome and the evolution of orchids

**DOI:** 10.1038/s41586-020-2524-1

**Published:** 2020-07-17

**Authors:** Guo-Qiang Zhang, Ke-Wei Liu, Zhen Li, Rolf Lohaus, Yu-Yun Hsiao, Shan-Ce Niu, Jie-Yu Wang, Yao-Cheng Lin, Qing Xu, Li-Jun Chen, Kouki Yoshida, Sumire Fujiwara, Zhi-Wen Wang, Yong-Qiang Zhang, Nobutaka Mitsuda, Meina Wang, Guo-Hui Liu, Lorenzo Pecoraro, Hui-Xia Huang, Xin-Ju Xiao, Min Lin, Xin-Yi Wu, Wan-Lin Wu, You-Yi Chen, Song-Bin Chang, Shingo Sakamoto, Masaru Ohme-Takagi, Masafumi Yagi, Si-Jin Zeng, Ching-Yu Shen, Chuan-Ming Yeh, Yi-Bo Luo, Wen-Chieh Tsai, Yves Van de Peer, Zhong-Jian Liu

**Affiliations:** 1 Shenzhen Key Laboratory for Orchid Conservation and Utilization, The National Orchid Conservation Center of China and The Orchid Conservation and Research Center of Shenzhen, Shenzhen, 518114 China; 2 Department of Plant Biotechnology and Bioinformatics, Ghent University, 9052 Gent, Belgium; 3 VIB Center for Plant Systems Biology, 9052 Gent, Belgium; 4 Orchid Research and Development Center, National Cheng Kung University, Tainan, 701 Taiwan; 5 Department of Life Sciences, National Cheng Kung University, Tainan, 701 Taiwan; 6 State Key Laboratory of Systematic and Evolutionary Botany, Institute of Botany, Chinese Academy of Sciences, Beijing, 100093 China; 7 College of Forestry, South China Agricultural University, Guangzhou, 510640 China; 8 Technology Center, Taisei Corporation, Nase-cho 344-1, Totsuka-ku, Yokohama, Kanagawa 245-0051 Japan; 9 Bioproduction Research Institute, National Institute of Advanced Industrial Science and Technology (AIST), Central 6, Higashi 1-1-1, Tsukuba, Ibaraki, 305-8562 Japan; 10 PubBio-Tech Services Corporation, Wuhan, 430070 China; 11 Graduate School of Science and Engineering, Saitama University, 255 Shimo-Okubo, Sakura-ku, Saitama, 338-8570 Japan; 12 NARO Institute of Floricultural Science (NIFS), 2-1 Fujimoto, Tsukuba, Ibaraki, 305-8519 Japan; 13 Institute of Tropical Plant Sciences, National Cheng Kung University, Tainan, 701 Taiwan; 14 Department of Genetics, Genomics Research Institute, Pretoria, 0028 South Africa; 15 College of Landscape Architecture, Fujian Agriculture and Forestry University, Fuzhou, 350002 China; 16 The Center for Biotechnology and BioMedicine, Graduate School at Shenzhen, Tsinghua University, Shenzhen, 518055 China; 17 Present Address: Biotechnology Center in Southern Taiwan, Agricultural Biotechnology Research Center, Academia Sinica, 741 Tainan, Taiwan

**Keywords:** Speciation, Genome

Correction to: *Nature* 10.1038/nature23897Published online 13 September 2017

In this Letter, the following affiliation was missing for author Ke-Wei Liu: School of Life Sciences, Beijing and Graduate School at Shenzhen, Tsinghua University, Shenzhen 518055, China. The original Letter has not been corrected.

