# Supplementary material for: The Apostasia genome and the evolution of orchids
Source: Nature. 2017 Sep 13;549(7672):379–83. doi: 10.1038/nature23897 (PMC7416622; doi:10.1038/nature23897)
Supplement: Supplementary Information — This file contains Supplementary Notes 1-3, Supplementary Figures 1-27, Supplementary Tables 1-23, Supplementary Data and additional references. (PDF 7375 kb) [file 41586_2017_BFnature23897_MOESM1_ESM.pdf]

doi:10.1038/nature23897

Supplementary Notes

1 Evolution of gene families

1.1 Evolution of gene family sizes

We determined the expansion and contraction of orthologous gene families using CAFÉ 2.2[1]. One hundred and thirteen gene families were expanded in the lineage leading to the orchids, whereas 1,047 families became smaller (**Figure 1**). Five hundred and twenty-two gene families were expanded in *A. shenzhenica* (six by a significant margin), compared to 557 (five by a significant margin) in *P. equestris* and 872 (34 by a significant margin) in *D. catenatum*. At the same time, 1,661 (four by a significant margin) gene families became smaller in *A. shenzhenica* compared to 1,384 (27 by a significant margin) in *P. equestris* and 703 (one by a significant margin) in *D. catenatum* (**Supplementary Tables 15–18**).

1.2 Orchidaceae-specific gene families

A four-way comparison of Orchidaceae (*A. shenzhenica*, *D. catenatum*, and *P. equestris*), dicots (*A. thaliana*, *P. trichocarpa*, and *V. vinifera*), Poales (*A. comosus*, *B. distachyon*, *O. sativa*, and *S. bicolor*), and a group of *M. acuminata* and *P. dactylifera* found 10,377 gene families to be shared by all taxa (**Extended Data Figure 3**). In addition, 474 gene families present in all three orchid genomes are found to be unique to Orchidaceae, suggesting that orchids have fewer unique gene families than the three dicots (522), but much more than the four Poales (180).

For the *A. shenzhenica*-specific gene families, we conducted a Gene ontology (GO) / Kyoto Encyclopedia of Genes and Genomes (KEGG) enrichment analysis via enrichmentpipeline (https://sourceforge.net/projects/enrichmentpipeline/) and found

enrichment of the GO Terms 'RNA-directed DNA polymerase activity', 'RNA-dependent DNA replication' (**Supplementary Table 19**). The Orchidaceae-specific gene families were found to be enriched for GO terms 'Cysteine-type peptidase activity', 'O-methyltransferase activity' and the KEGG pathways 'Flavone and flavonol biosynthesis', 'Stilbenoid, diarylheptanoid and gingerol biosynthesis' (**Supplementary Tables 20** and **21**). The monocot-specific gene families were enriched for the GO terms 'two-component response regulator activity', 'solute antiporter activity', 'two-component signal transduction system', 'signal transducer activity' and 'molecular transducer activity', 'hydrogen ion transmembrane transporter activity' and the KEGG pathways 'Plant hormone signal transduction' and 'RNA polymerase' (**Supplementary Tables 22** and **23**).

O-methyltransferases can collectively mono- or polymethylate a great number of plant natural products. The methylation of plant natural products can alter their solubility and intracellular compartmentalization and can increase their antimicrobial activity[2]. For instance, amongst the genes with O-methyltransferase activity, Ash015798 is highly expressed in stem and seed, while significant expression of Ash015796 is detected in floral buds, stems, leaves and seeds (**Supplementary Figure 21**). Flavonoids and related phenolic compounds have essential functions in protecting plants against pathogens. The evolution of flavonoids has enabled vascular plants to cope with pathogen attacks and damaging UV light[3]. Ash006087, a gene involved in flavone and flavonol biosynthesis, and predominantly expressed in stems and roots, might play a protective role in vegetative tissues (**Supplementary Figure 22**). Stilbenes are a small family of plant secondary metabolites that are found in a limited number of plant species, such as *Vitis sylvestris*[4]. Stilbenes are important phytoalexins that accumulate in response to various biotic and abiotic stresses, and increasing evidence suggests a positive correlation between the stilbene content of plants and disease resistance[5]. The two tandem duplicates, Ash006321 and Ash006322, which are involved in the biosynthesis of stilbenoids, diarylheptanoids, and gingerol,

are both highly expressed in flower buds, stems and seeds, and likely have important functions against stresses (**Supplementary Figure 23**).

In addition, the GO-term 'cysteine-type peptidase activity' seem to be specifically enriched (**Supplementary Table 20**). Cysteine proteases are an important class of enzymes implicated in both developmental and defense-related programmed cell death and other biological processes in plants[6]. The *Arabidopsis* papain-like cysteine protease CEP1 for instance is involved in tapetal programmed cell death and pollen development[7]. In addition, in *Arabidopsis,* cysteine proteases such as aleurain-like proteases, cathepsin B-like proteases, and vacuolar processing enzymes are correlated with the remobilization of seed storage proteins during seed germination[6]. Among *A. shenzhenica* cysteine-type peptidase genes, significant expression of Ash003370 could be detected in the flower bud, stem, leaf, and seed (**Supplementary Figure 24**). Gene Ash003370 thus likely plays important roles in the reproductive and vegetative development of *A. shenzhenica*, but further studies are necessary to unravel its precise functional role.

2 Whole genome duplication

2.1 K_S distributions and absolute phylogenomic dating

Analyses of the number of synonymous substitutions per synonymous site (K_S) in *A. shenzhenica* for both the whole paranome (the set of all duplicated genes in the genome, **Extended Data Figure 4a**) and 'anchor' duplicates retained in co-linear regions only (i.e., excluding duplicates from small-scale duplications, **Extended Data Figure 4b**) consistently identified a clear peak of duplicates with a K_S value close to 1. The K_S distributions generated from the genomes of the epidendroid orchids *P. equestris*[8] and *D. catenatum*[9] and from transcriptomes of nine additional orchids (together covering all five orchid subfamilies) all show similar K_S peaks with K_S values of 0.7 to 1.1 (**Supplementary Figure 2**). In the apostasioid *Neuwiedia*

malipoensis, a prominent second peak at a much lower K_S value of about 0.25 may signify a more recent and *Neuwiedia*-specific second WGD (in contrast, the peaks at K_S values < 0.2 as apparent in the genomes of *D. catenatum*, *P. equestris* and *A. shenzhenica* stem from background (tandem) duplications and most likely do not signify additional recent WGDs).

We constructed K_S-based age distributions of the one-to-one orthologues of *A. shenzhenica* and *Asparagus officinalis* (asparagus, family Asparagaceae, a sister family to Orchidaceae) (GenBank accession GCF_001876935.1), *A. shenzhenica* and *P. equestris*, *A. shenzhenica* and *D. catenatum*, and *P. equestris* and *D. catenatum* (each representing the divergence event between the two respective species) and compared these to the distributions of the duplicated anchors from each of the three orchids for which we have a genome (**Figure 2a**). The peaks of the three anchor-pair distributions all have lower K_S values than the peak of the *A. shenzhenica*–*A. officinalis* orthologue distribution, indicating that the WGD signatures are specific to Orchidaceae and not shared with other non-orchid Asparagales (see also **Supplementary Figures 2** and **25)**. They also all have higher K_S values than the peak of the *P. equestris*–*D. catenatum* orthologue distribution, confirming a WGD event that is shared at least between these two species, as reported previously[9]. The anchor-pair distributions of *A. shenzhenica* and *P. equestris* are also slightly shifted towards higher K_S values compared with the *A. shenzhenica*–*P. equestris* and *A. shenzhenica*–*D. catenatum* orthologue distributions (each of which represents the divergence between the *A. shenzhenica* lineage and the rest of the Orchidaceae), whereas the anchor pair distribution of *D. catenatum* largely overlaps with these two orthologue distributions. *D. catenatum* likely has a slightly lower substitution rate, as hinted by the slightly “younger” peak of the *A. shenzhenica*–*D. catenatum* orthologue distribution compared to the *A. shenzhenica*–*P. equestris* orthologue distribution (also compare the orchid–*A. officinalis* orthologue distributions in **Supplementary Figure 2,** long-dashed versus dashed vertical yellow lines for *D. catenatum* and *P. equestris*).

These patterns suggest that the WGD(s) and the initial orchid speciation events occurred relatively closely in time and that the WGD events evident in the three orchid genomes (and, by extension, those in the orchid transcriptomes) could represent a single event, slightly older than their divergence, and thus shared among all orchids. However, the distance between the peaks is small, there is substantial overlap among the distributions and the number of anchor pairs we could extract is relatively low for all three species. In addition, some heterogeneity in substitution rate between these species is expected given the age of the events and this is apparent in the one-to-one orthologue K_S distributions with *A. officinalis* (yellow distributions and long-dashed vertical yellow lines versus dashed vertical yellow lines in each panel of **Supplementary Figure 2**).

We performed absolute phylogenomic dating[10] of the WGD event identified from the *A. shenzhenica* genome to determine its age in relation to orchid phylogeny. Paralogous gene pairs present under the WGD peak in the *A. shenzhenica* K_S distributions (**Extended Data Figures 4a** and **b**) were dated (see **Methods**) and the resulting absolute age distribution showed a peak at 74 million years ago (Ma) with a 90% confidence interval of 72–78 Ma (**Extended Data Figure 5**). This estimate of the date of the WGD event in the *A. shenzhenica* lineage coincides with the date estimated for the WGD event in the *P. equestris* lineage (76 Ma, 90% CI: 72–81 Ma[8]). Estimates for the crown age of extant orchids, i.e., the time of divergence of the *A. shenzhenica* lineage from the lineage that gave rise to the rest of the Orchidaceae, vary widely and range from 54 Ma to 121 Ma (Ramírez *et al.*[11]: 71–90 Ma, youngest mean minus 1 SD to oldest mean plus 1 SD; Gustafsson *et al.*[12]: 63–92 Ma, 95% HPD; Chen *et al.*[13]: 54–82 Ma, 95% HPD; Chomicki *et al.*[14]: 75–121 Ma, 95% HPD; Givnish *et al.*[15, 16]: 80–100 Ma, 95% CI; see also **Figure 1** and **Extended Data Figure 2**), but again suggest that the initial divergence of extant orchid lineages and any single or multiple ancient orchid WGD event(s) likely occurred closely together in time. Some of these ranges would support the possibility of a single shared WGD

in the most recent common ancestor of extant orchids indicated in the K_S-based age distributions, as discussed above (**Figure 2a**), though our date estimate for such an event would fall towards the lower end of most of those range estimates.

2.2 Co-linearity and synteny analyses

Co-linearity analysis (see **Methods**) of *A. shenzhenica* indicated that 43.85% of the genome retains paralogous genes from WGD events, with 7,271 (33.31%), 1,423 (6.52%), 669 (3.06%), and 210 (0.96%) genes on co-linear regions with two, three, four, and five paralogous segments, respectively, suggesting two WGD events that can be identified in the current *A. shenzhenica* genome (**Figure 2b** and **Supplementary Figure 3**). One of the WGD events seems much more recent than the other because co-linear regions consisting of three and four segments are much rarer than those with two, which almost cover one third of the genome. The few co-linear regions with five segments (making up less than 1% of the genes) might be remnants of a more ancient third WGD event.

To circumscribe the two WGD events, we compared the genome of *A. shenzhenica* with the genomes of several other flowering plants: the earliest-diverging extant angiosperm *Amborella trichopoda*, the dicot *Vitis vinifera*, and the two monocots *Ananas comosus* (pineapple, order Poales) and *Asparagus officinalis*. Due to the fragmented nature of the current genome assembly of *A. shenzhenica*, counting the number of co-linear segments in a comparison of *A. shenzhenica* with other species did not directly unveil the number of individual WGDs. We thus illustrated duplication depth, i.e., the number of overlapping co-linear segments at a broader genomic region, by mapping such co-linear segments onto their corresponding orthologous regions in the other species (see **Methods**). The pairwise comparisons with *A. trichopoda* and *V. vinifera* both support at least two WGDs in *A. shenzhenica* (**Supplementary Figures 4 and 5**) consistent with the

doi:10.1038/nature23897

A. shenzhenica self-comparison (**Figure 2b**).

In comparison with *A. comosus*, for each co-linear segment in *A. shenzhenica* we found mostly up to four orthologous co-linear segments in *A. comosus* and vice versa for each *A. comosus* segment mostly up to four orthologous segments in *A. shenzhenica* (**Extended Data Figure 6**). Such a 4:4 pattern is consistent with the two monocot WGDs that have been proposed in the evolutionary history of *A. comosus*, the τ WGD[17, 18] shared by most monocots and the σ WGD shared by all Poales[18]. In some of these co-linear regions, four co-linear segments in *A. shenzhenica* corresponded to a specific set of four co-linear segments in *A. comosus* (on chromosomes LG4, LG13, LG18 and LG23) which have been shown to originate from one of the seven ancestral pre-τ-WGD chromosomes in monocots (Anc6) (**Extended Data Figure 6** and Figures 2 and 3c in Ming *et al*.[18]). This and the 4:4 co-linearity pattern indicate that *A. shenzhenica* followed a similar evolutionary trajectory with regard to WGDs as *A. comosus*, with one (Orchidaceae-)lineage-specific WGD in addition to the shared τ WGD. Consistently, by tightening and relaxing the criterion influencing duplication depth (i.e., the required number of anchors on the co-linear segments) we could distinguish these two WGDs (and a putative older third event) (**Supplementary Figures 6** and **7**). Similarly, the comparison between *A. shenzhenica* and *A. officinalis* showed much the same pattern as the comparison between *A. shenzhenica* and *A. comosus* (suggesting *A. officinalis* also has one independent Asparagaceae-specific WGD; also see **Supplementary Figure 25**), but with less paralogous segments retained from the τ WGD (**Supplementary Figure 8**).

2.3 Gene tree analyses

To further corroborate the timing of the WGD events relative to orchid and monocot divergences, we constructed and analysed gene trees that included genes

from 12 orchid species across the five subfamilies of Orchidaceae, four non-orchid Asparagales, three Poales, and *A. trichopoda* (see **Methods**). We selected the subset of trees that contained at least one pair of duplicated anchor genes from co-linear regions from one of the three orchids with complete genome information (*A. shenzhenica*, *D. catenatum* and *P. equestris*; i.e., we only used gene families that also had 'spatial/structural' evidence of a WGD event) and mapped the coalescence points of these anchor pairs onto the species phylogeny (see **Methods**; see also **Supplementary Figure 20**). Overall, the anchor-pair coalescence points in nearly half of such gene family trees, containing the majority of anchor pairs from *D. catenatum* and *P. equestris* and the largest fraction of anchor pairs from *A. shenzhenica*, mapped onto the orchid stem branch, providing support for a WGD event shared by all orchids (**Figure 3**). The anchor pairs in most of the remaining gene family trees mapped either onto the branch leading to all the included monocots, again providing support for the ancient τ WGD event shared by these monocots[17, 18], or onto the two first diverging/descending branches from the orchid ancestor (**Figure 3**). A low number of anchor pairs from *D. catenatum* and *P. equestris* mapped along the internal orchid branches leading to Epidendroideae. However, even though the largest fraction of *A. shenzhenica* anchor pairs mapped onto the orchid stem branch (76), a substantial fraction of anchor pairs (70) actually mapped onto the Apostasioideae stem branch. These could be considered support for an additional WGD event in the Apostasioideae lineage. However, the co-linearity analyses discussed above suggest only one WGD event unique to Orchidaceae in the evolutionary history of *A. shenzhenica*. Therefore, we believe the large number of anchor points mapped on the Apostasioideae stem branch to be due to phylogenetic discordance as a result of the probably very short time interval between the shared WGD event and the divergence of Orchidaceae.

Such phylogenetic discordance could theoretically be due to incomplete lineage sorting (or other difficulties in tree inference when short branches are involved due to short time intervals between successive phylogenetic events, or, alternatively, due to

homeologous recombination). A possible scenario for a specific gene (tree) is illustrated in **Supplementary Figure 26**. A gene in the common ancestor of all extant orchids was polymorphic before the shared orchid-specific WGD, i.e., it had two diverging alleles, G and G'. Then both alleles got duplicated during the WGD, resulting in two paralogous loci with a total of four alleles that were retained in the early orchid polyploid. If the ancestral orchid speciation event followed relatively soon after the WGD event, all four alleles may have been retained in the two diverging orchid species, which gave rise to the ancestors of the current Apostasioideae (which includes *A. shenzhenica*) and the ancestors of the rest of the orchid families including the lineage eventually leading to Epidendroideae (which includes *P. equestris* and *D. catenatum*), respectively. Over time, if both paralogous loci are retained in the current orchids and if we assume, for simplicity, that alleles eventually became fixed at the paralogous loci, i.e., two of the four alleles got lost owing to genetic drift or selection, then only two of the alleles were retained at the paralogous loci forming an anchor pair. As a result, the coalescence points of such anchor pairs in the current species depend on which of the alleles were finally fixed at the paralogous loci in a species. There are in total 36 possible combinations of any two fixed alleles from each species (in a species/gene tree with two species), but only three possible coalescence points on branches in the species-level phylogenetic tree, i.e., the stem branch of Orchidaceae or one of the two early-diverging branches of Orchidaceae after the ancestral speciation event. We elaborate on four examples in **Supplementary Figure 26**. If all the retained anchors are all from one ancestral allele (**Supplementary Figure 26A**, all from allele G), the coalescence points of the two anchor pairs are both the duplication (i.e., WGD) event and they fall onto the orchid stem branch. If one ancestral species kept anchors from different alleles (**Supplementary Figure 26B** and **C**, species A and E, respectively, one from allele G and the other from allele G') and the other ancestral species kept anchors from the same allele (**Supplementary Figure 26B** and **C**, species E and A, respectively, all from allele G), then the coalescence point of one of the anchor pairs is the duplication

event (e.g., of E_1 and E_2 in **Supplementary Figure 26B**) and the coalescence point of the other anchor pair is actually the divergence event of the two ancestral alleles (e.g., of A_1 and A'_2 in **Supplementary Figure 26B**). Therefore, both coalescence points map onto the orchid stem branch but seemingly indicate different phylogenetic events. In the fourth example, if one ancestral species retained both anchors from one allele (**Supplementary Figure 26D**, species A, both from allele G') while the other ancestral species retained both anchors from the other allele (**Supplementary Figure 26D**, species E, both from allele G), then although the two coalescence points reflect the duplications of each of the two alleles (during shared orchid WGD event) they actually erroneously map onto the two orchid subbranches.

To substantiate our hypothesis on discordance in anchor-pair mapping due to incomplete lineage sorting, we additionally built gene families as described in **Methods** ('Identification of WGD events in *A. shenzhenica* and phylogenomic analyses'), but now only using the three orchid genomes, plus *Asparagus officinalis* and *Amborella trichopoda*. Phylogenetic trees of 2,573 gene families were reconstructed and rooted for gene families that had at least five genes, with at least one gene from *A. trichopoda*, *A. officinalis*, and *A. shenzhenica* plus at least one gene from either *P. equestris* or *D. catenatum* and at least one duplicate pair in at least one of the three orchid species. For computational reasons, gene families with more than 200 genes were removed from further analysis. We traversed this full set of the 2,573 rooted gene trees to explore tree topologies related to duplication events in orchids based on all paralogues and not just the limited set of anchor pairs (**Supplementary Figure 27**).

We first extracted subtree(s) from each node for which the node itself included genes only from the orchids and the sister clade had genes from *A. officinalis* and/or *A. trichopoda*. Subtrees that did not have genes from at least *A. shenzhenica* and one other orchid plus a pair of paralogues were removed. Based on these criteria, we obtained 2,085 subtrees from 1,864 rooted gene trees. The subtrees were explored to

look for expected topologies supporting the corresponding topologies under the four different scenarios described in **Supplementary Figure 26**. The expected gene tree topologies, including those with gene loss and/or incomplete sampling taken into account (see below), are illustrated in **Supplementary Figure 26E**. The bootstrap values on the branches leading to nodes that were used to distinguish different topologies, i.e., whether the nodes represented speciation or duplication events, were used to evaluate the support for such events with a cutoff of greater than or equal to 50%. We found 167, 39, 45, and 72 gene trees that showed subtrees with the expected topologies under the respective four scenarios as depicted in **Supplementary Figure 26A–D**. The first three topologies thus support a (whole-genome) duplication event that occurred before the divergence of Orchidaceae, while the gene trees of the last topology could erroneously support two duplication events, each occurring on one of the two subbranches of Orchidaceae (**Supplementary Figure 26E**). Due to extensive gene loss (the most common fate for duplicated genes) and/or incomplete sampling, the resulting gene trees may have a second set of four prevalent topologies consisting of only three retained genes (i.e., one lost or non-sampled gene). Two of these topologies support a coalescence point on the stem branch of Orchidaceae (we found 548 and 296 such gene trees), one topology could erroneously support a coalescence point on the subbranch of Orchidaceae leading to Epidendroideae (we found 429 such gene trees), and the fourth topology could erroneously support a coalescence point on the stem branch of Apostasioideae (we found 267 such gene trees) (**Supplementary Figure 26E**). Considering all these scenarios together, we found that there were fewer gene (sub)trees with duplicated genes from *A. shenzhenica* than gene (sub)trees with duplicated genes from Epidendroideae, indicating massive gene loss in *A. shenzhenica*. In total, 1,450 gene families with 1,620 subtrees were considered among the 1,864 gene families with 2,085 subtrees.

3 MIKC*-type genes and the evolution of the pollinium

MIKC*-type genes are one of the major regulators of the male gametophytic developmental programme. These genes were shown to have a conserved function in both *Arabidopsis* and rice pollen development[19]. MIKC*-type genes are present in all major groups of land plants, including bryophytes, lycophytes, ferns, gymnosperms, and angiosperms[20]. In seed plants, two different monophyletic groups of MIKC*-type genes could be identified, the P- and S-subclades[20]. The former, however, is absent in all orchids except *A. shenzhenica* (**Extended Data Figure 8**).

In rice, there are two S-subclade MIKC* genes, *MADS62* and *MADS63*, and one P-subclade *MADS68* gene. All three rice genes are specifically expressed late in pollen development[19], which resembles the expression of *AGL66*, *AGL104*, and *AGL30* in *Arabidopsis*[21]. This suggests that a pollen-specific expression pattern was already established in the most recent common ancestor of monocots and eudicots. The single knockdown or knockout lines, respectively, of the S-subclade *MADS62* and *MADS63* in rice did not show a mutant phenotype, but lines in which both S-subclade genes were affected showed severe defects in pollen maturation and germination[19]. This indicates that the two S-subclade genes of *MADS62* and *MADS63* act redundantly in pollen development, as do their homologs *AGL66* and *AGL104* in *Arabidopsis*[22, 23].

In *Arabidopsis* no complete knockout or a knockdown of P-subclade genes were achieved. The down-regulation of the sole rice P-subclade gene *MADS68* in RNAi transgenic lines resulted in defects in pollen maturation and germination[23]. Taken together, both S- and P-subclade MIKC*-type genes confer an indispensable and highly conserved function in pollen maturation and germination in the monocot rice as well as in the eudicot *Arabidopsis*.

Therefore, it is possible that the loss of the P-subclade members of MIKC*-type genes is related to the evolution of the pollinium. During pollen development, a pollen

mother cell undergoes meiosis to produce four haploid microspores, which are first packaged in common callose. Subsequently, due to the decomposition of the tetrad callose wall, the microspores are separated and form the pollen grains[24]. In orchids, with the exception of *Apostasia* and *Neuwiedia*, the pollen callose wall is not decomposed, leading to the formation of a tetrad of pollen grains as a unit, and these ‘sticky balls’ together form the pollinium[24, 25]. Because the P-subclade genes have been lost in most orchids but not in *A. shenzhenica* and because their presence seems to correlate with the presence of a pollinium, we propose that the P-subclade genes provide the ability to decompose the callose wall and that their loss leads to the production of pollen that aggregate into pollinia (**Figure 4a, c**), which however will need to be confirmed with experimental data. The formation of pollinia has been an important transition during the evolution of orchids. This transition, combined with the evolution and formation of the flower lip and gynostemium, has proven very useful for efficient insect pollination and likely lead to the emergence of reproductive barriers[15].

doi:10.1038/nature23897

Supplementary Figures

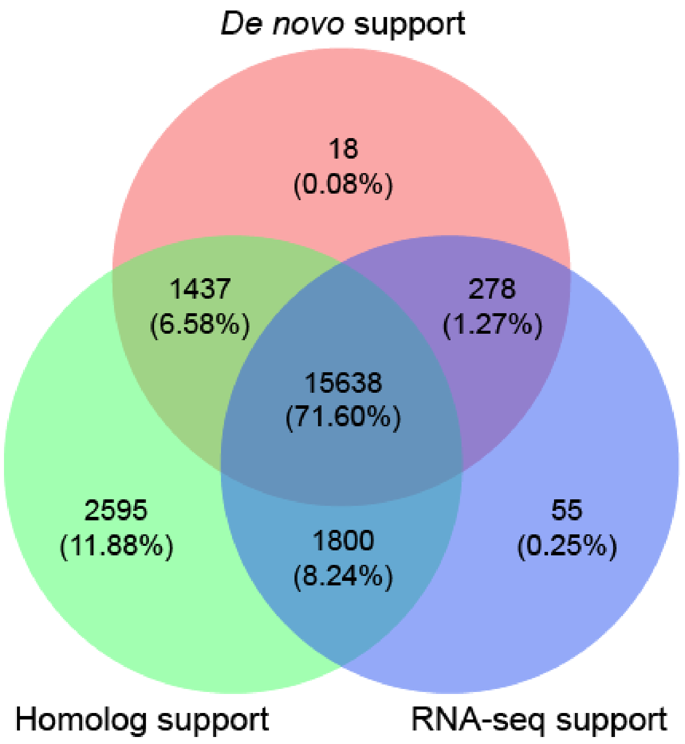

Supplementary Figure 1 | Evidence for gene annotation of *A. shenzhenica*. The respective support of final gene sets by the three methods (*De novo* prediction, Homology searching and RNA-seq mapping) are shown.

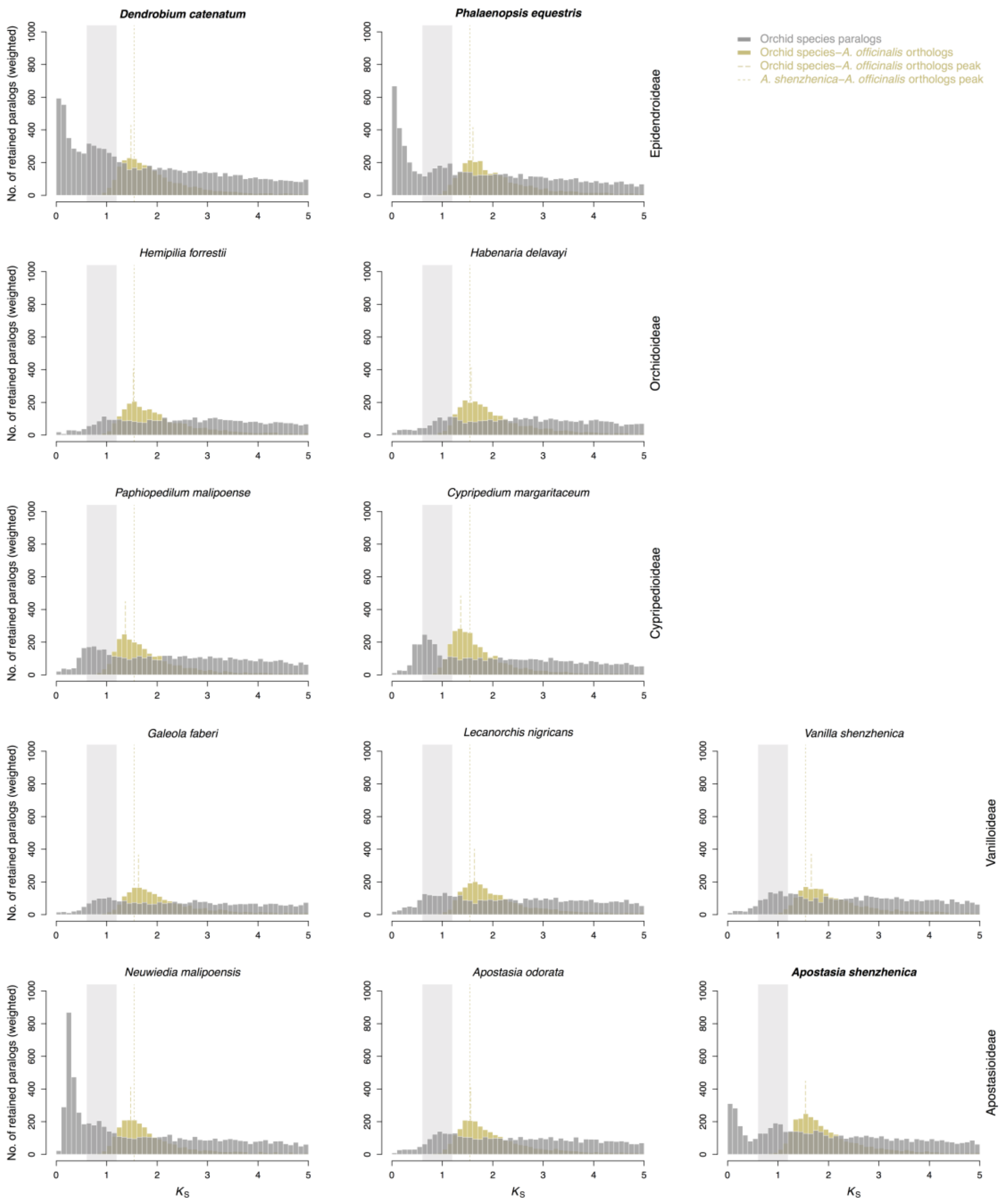

Supplementary Figure 2 | Distribution of synonymous substitutions per synonymous site (K_S) of the whole paranome for three orchid genomes and nine orchid transcriptomes. K_S distributions of paralogues are shown in gray. The light gray rectangle in the background of each plot highlights the K_S range from 0.6–1.2 in which putative WGD peaks can be identified for all 12 orchid species shown. K_S distributions of one-to-one orthologues between each orchid species and *A. officinalis*, representing their time of divergence, are shown in yellow, with long-dashed lines indicating the peak (based on KDE) of the distributions. The K_S value of the peak of

the *A. shenzhenica*–*A. officinalis* one-to-one orthologue distribution is shown for comparison as a dashed line in each plot, indicating potential differences in substitution rates compared to *A. shenzhenica*. The three orchids with genomes are in bold.

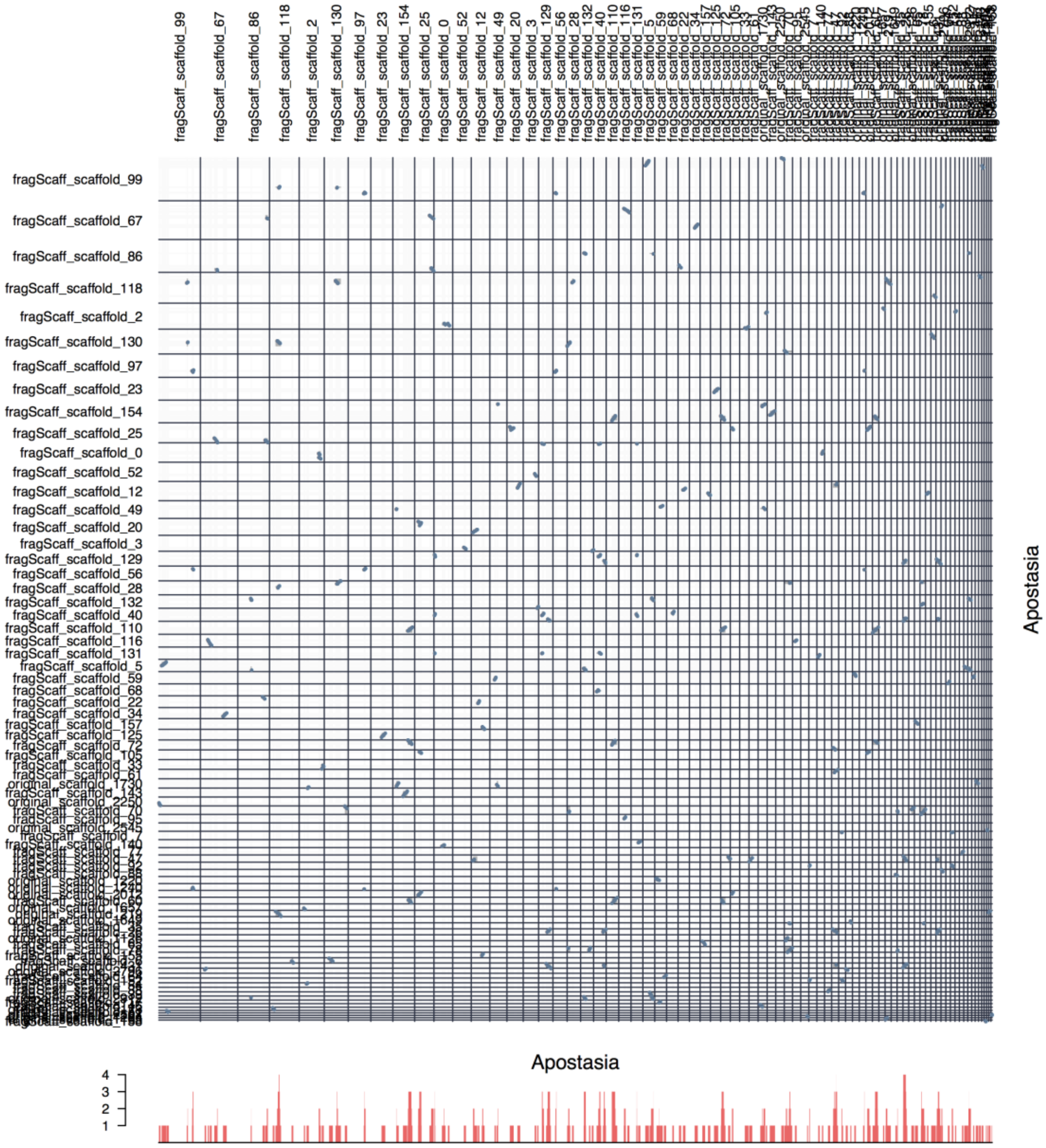

Supplementary Figure 3 | 'Synteny' dot plot of the self-comparison of *A. shenzhenica*. Only co-linear segments with at least 5 anchor pairs are shown. The red bars below the dot plot illustrate the duplication depths (the number of connected co-linear segments overlapping at each scaffold/chromosomal position; see **Methods**).

doi:10.1038/nature23897

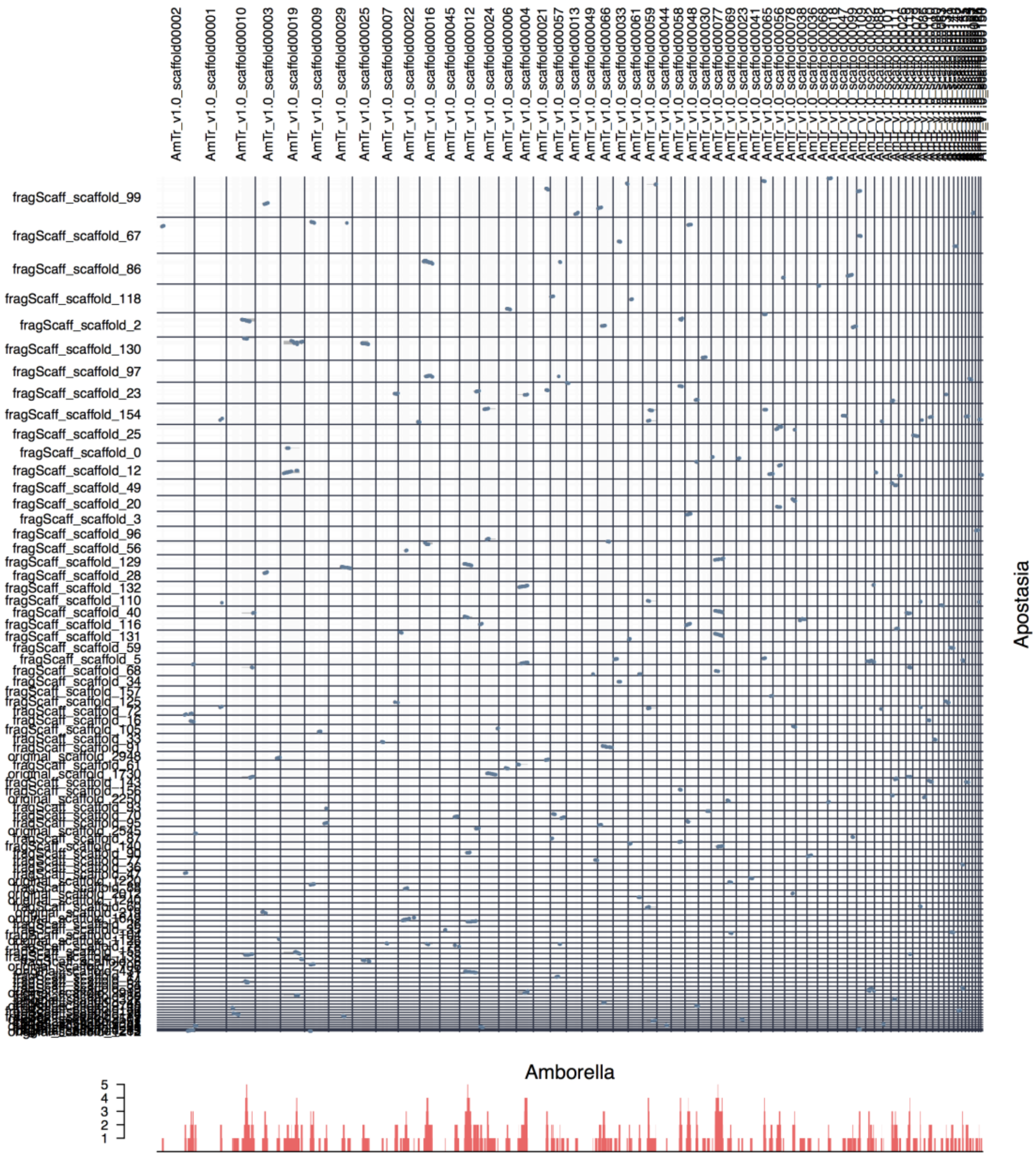

Supplementary Figure 4 | 'Synteny' dot plot of the comparison between *A. shenzhenica* and *A. trichopoda*. Only co-linear segments with at least 5 anchor pairs are shown. The red bars below the dot plot illustrate the duplication depths (the number of connected co-linear segments overlapping at each scaffold/chromosomal position; see **Methods**).

doi:10.1038/nature23897

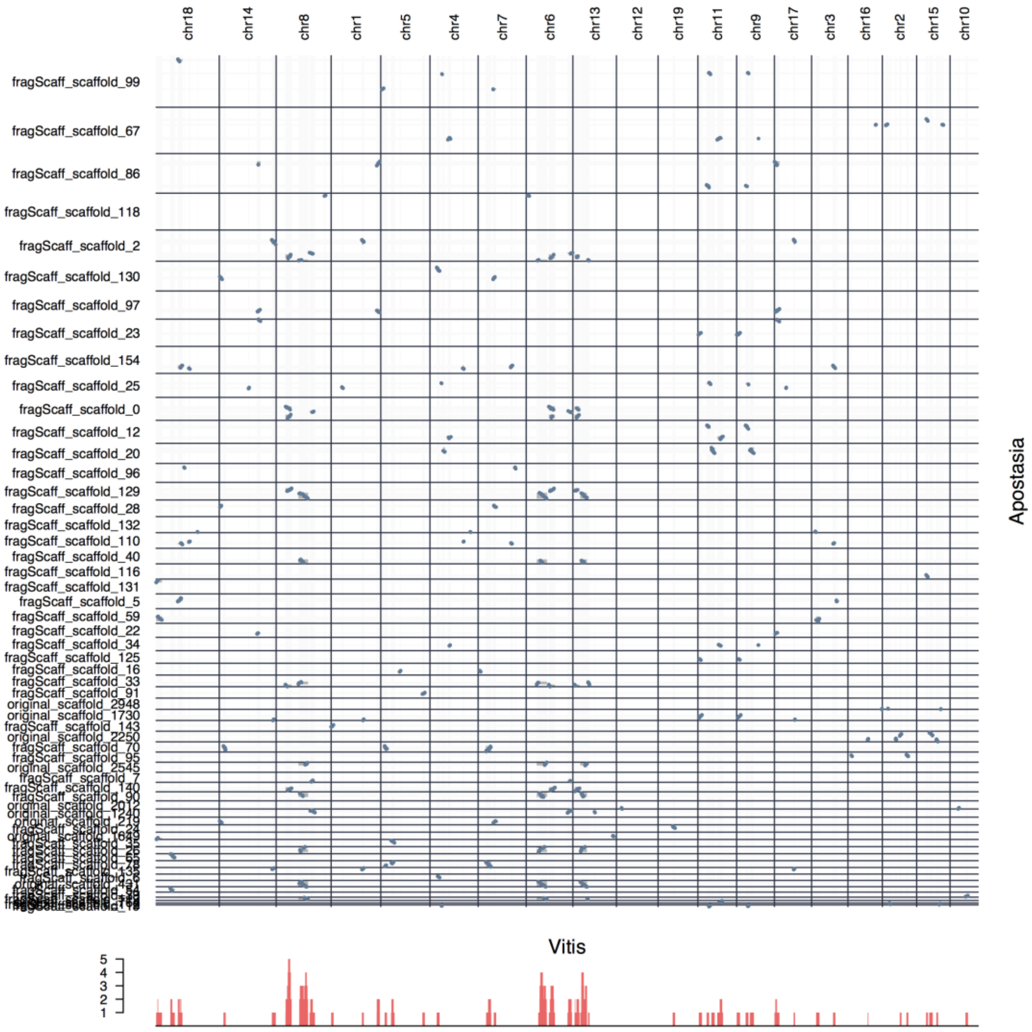

Supplementary Figure 5 | ‘Synteny’ dot plot of the comparison between *A. shenzhenica* and *V. vinifera*. Only co-linear segments with at least 10 anchor pairs are shown. The red bars below the dot plot illustrate the duplication depths (the number of connected co-linear segments overlapping at each scaffold/chromosomal position; see **Methods**). Only connected co-linear segments with at least 10 anchor pairs were used to calculate the duplication depths.

doi:10.1038/nature23897

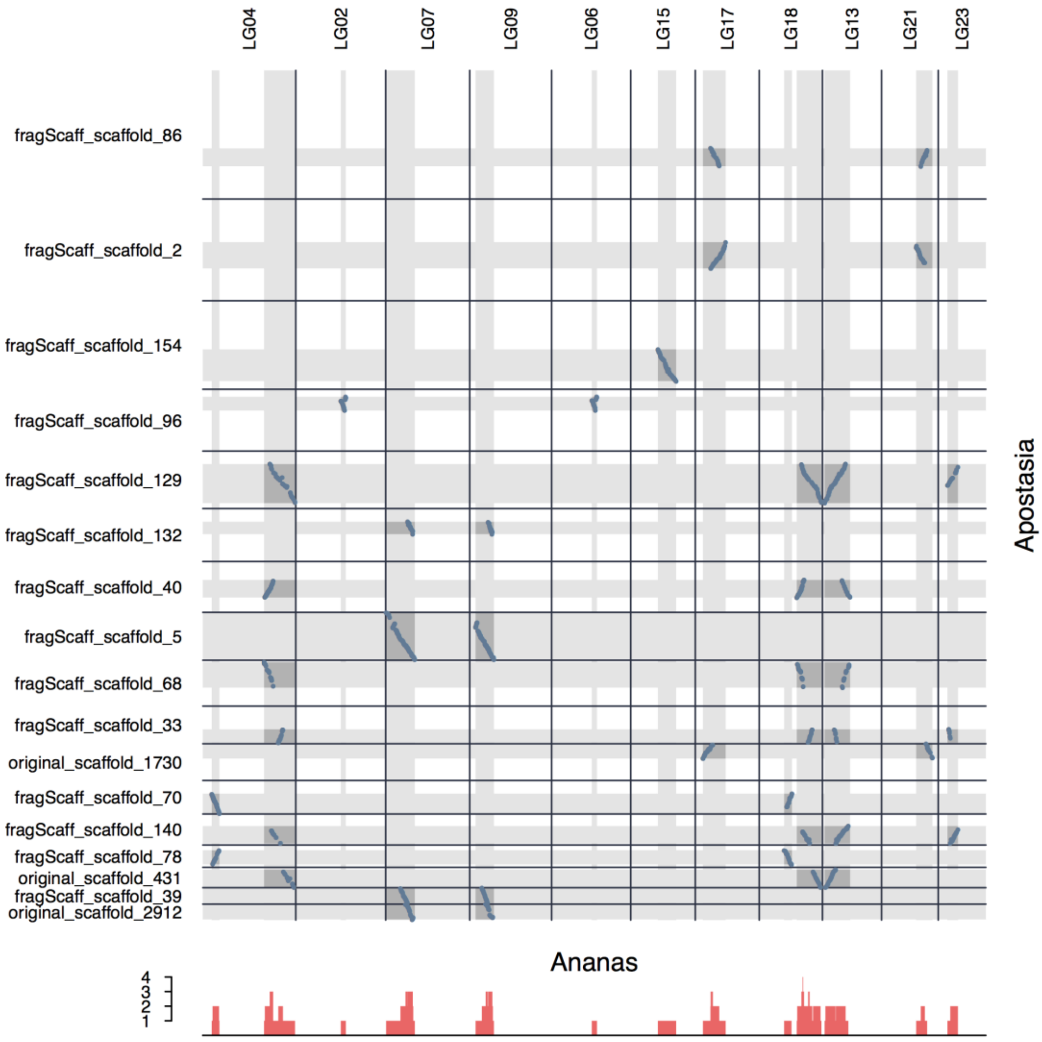

Supplementary Figure 6 | 'Synteny' dot plot of the comparison between *A. shenzhenica* and *A. comosus*. Only co-linear segments with at least 30 anchor pairs are shown. The sections on each scaffold with co-linear segments between are shown in grey. The red bars below the dot plot illustrate the duplication depths (the number of connected co-linear segments overlapping at each scaffold/chromosomal position; see **Methods**). Only connected co-linear segments with at least 15 anchor pairs were used to calculate the duplication depths.

doi:10.1038/nature23897

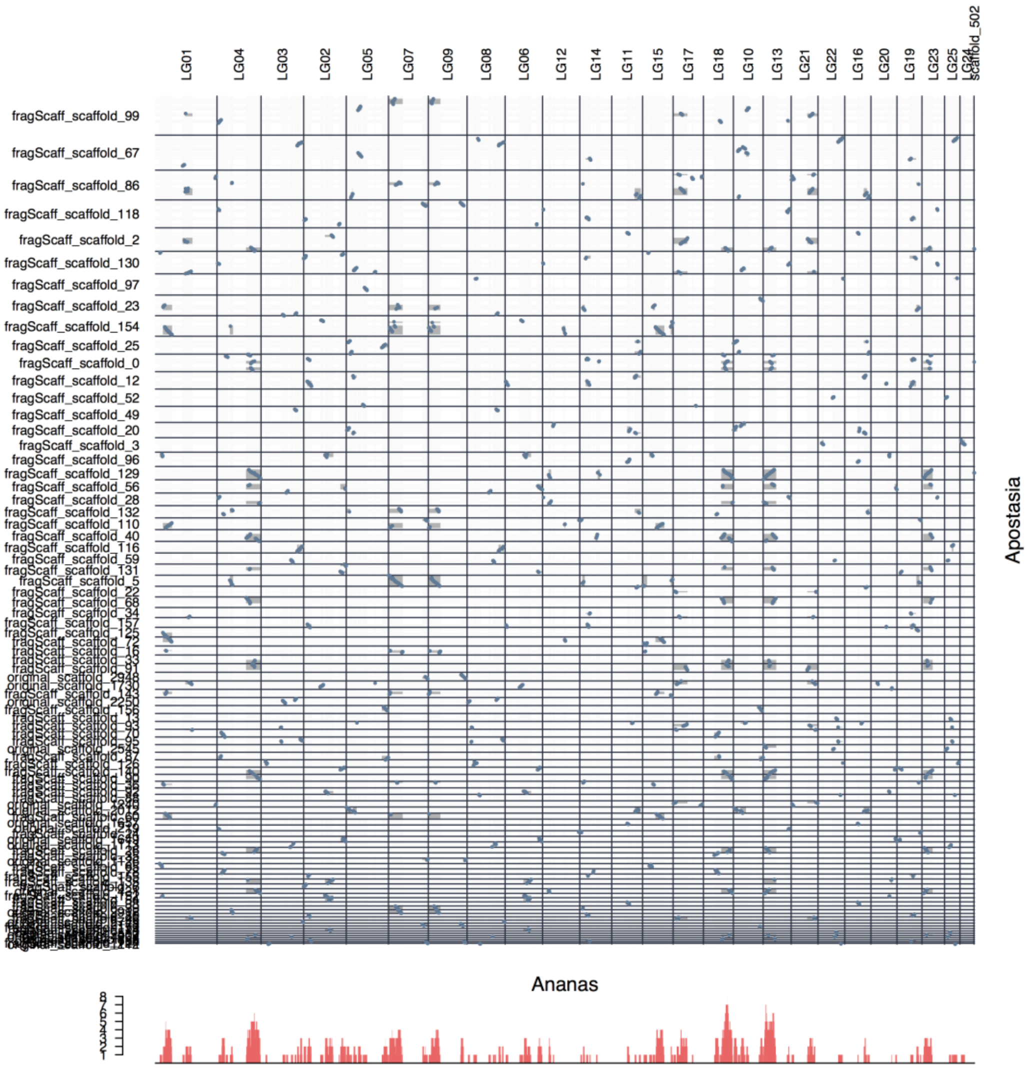

Supplementary Figure 7 | ‘Synteny’ dot plot of the comparison between *A. shenzhenica* and *A. comosus*. Only co-linear segments with at least 10 anchor pairs are shown. The sections on each scaffold with co-linear segments between are shown in grey. The red bars below the dot plot illustrate the duplication depths (the number of connected co-linear segments overlapping at each scaffold/chromosomal position; see **Methods**). Only connected co-linear segments with at least 10 anchor pairs were used to calculate the duplication depths.

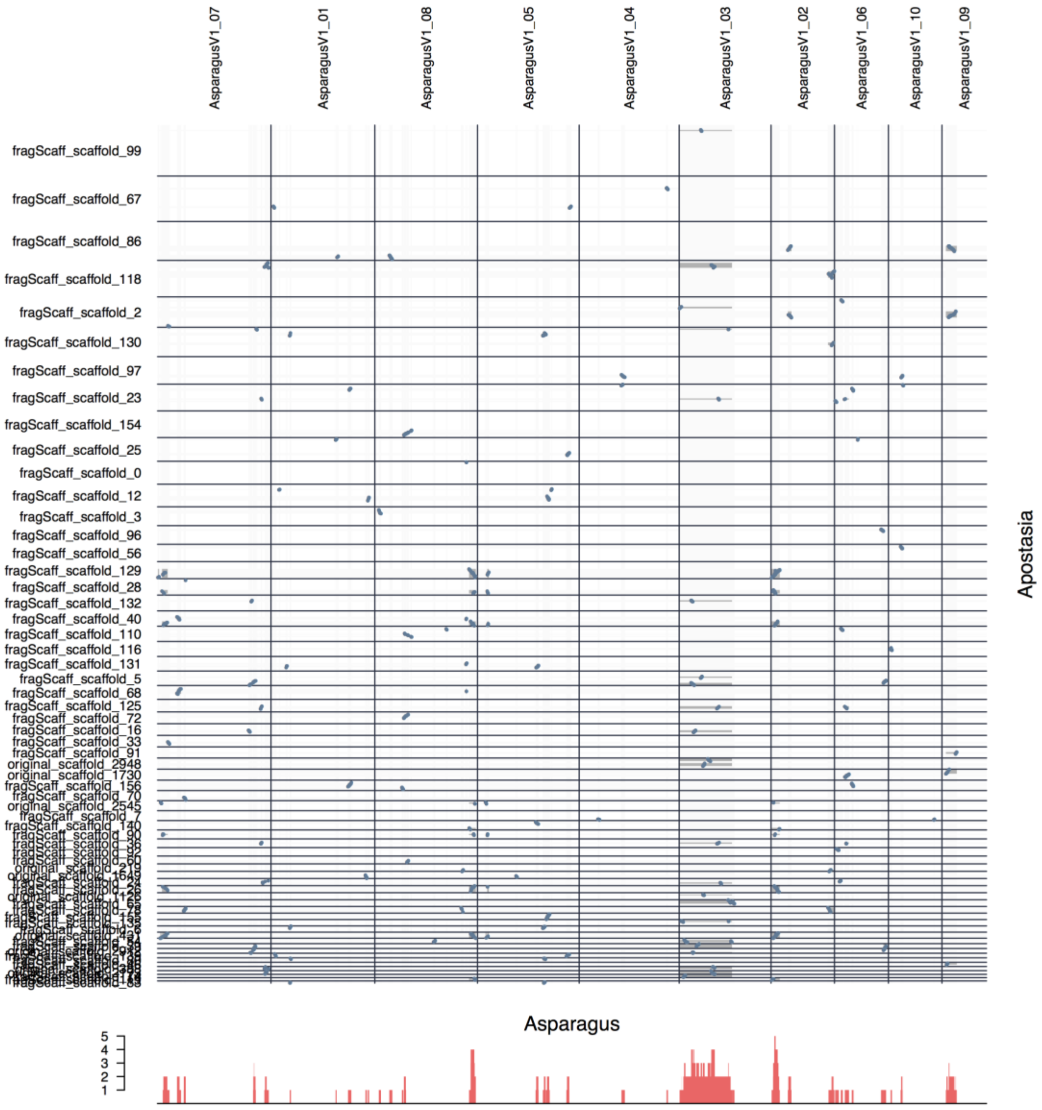

Supplementary Figure 8 | ‘Synteny’ dot plot of the comparison between *A. shenzhenica* and *A. officinalis*. Only co-linear segments with at least 10 anchor pairs are shown. The sections on each scaffold with co-linear segments between are shown in grey. The red bars below the dot plot illustrate the duplication depths (the number of connected co-linear segments overlapping at each scaffold/chromosomal position; see **Methods**). Only connected co-linear segments with at least 10 anchor pairs were used to calculate the duplication depths.

doi:10.1038/nature23897

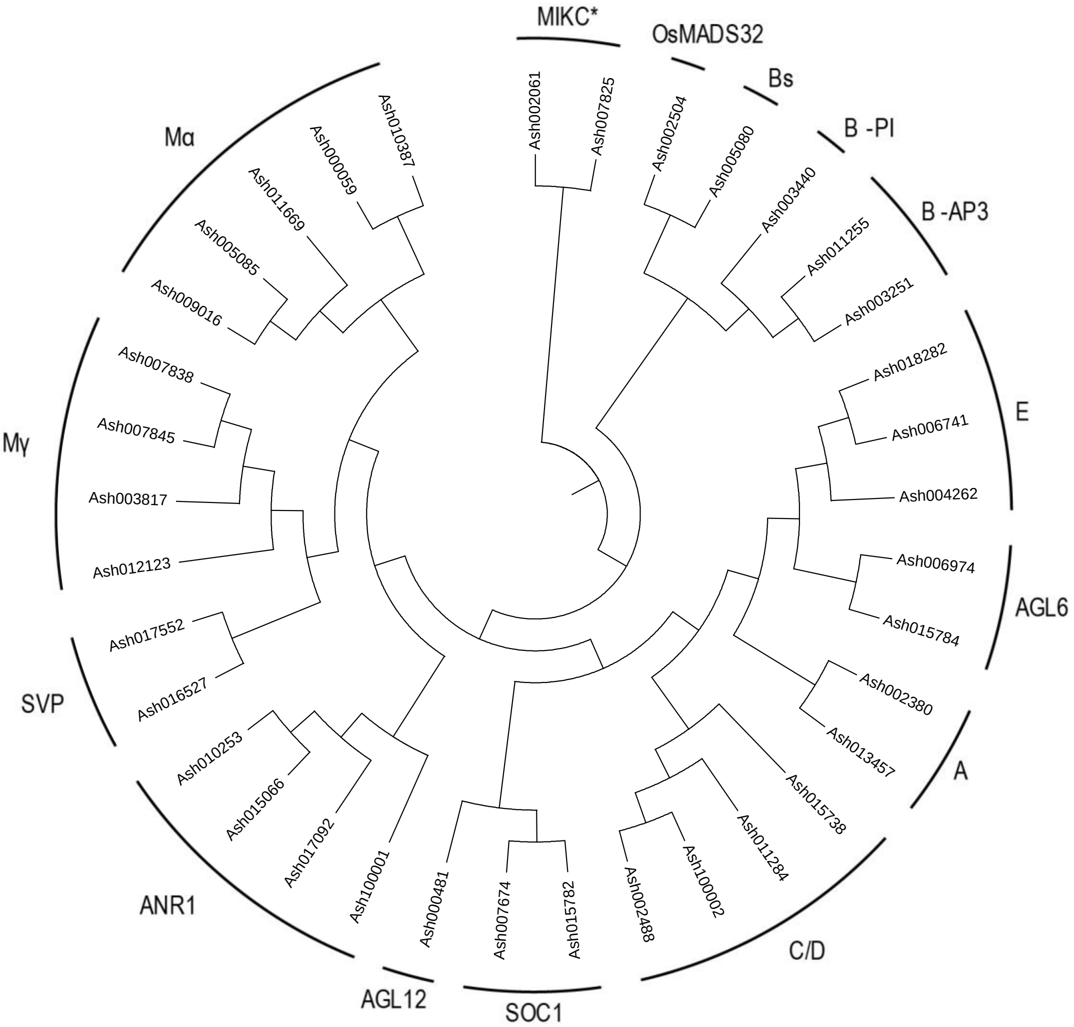

Supplementary Figure 9 | The MADS-box genes involved in orchid morphological evolution. MADS-box genes in *A. shenzhenica*, which contains 36 putative functioning MADS-box genes belonging to 15 subfamilies.

doi:10.1038/nature23897

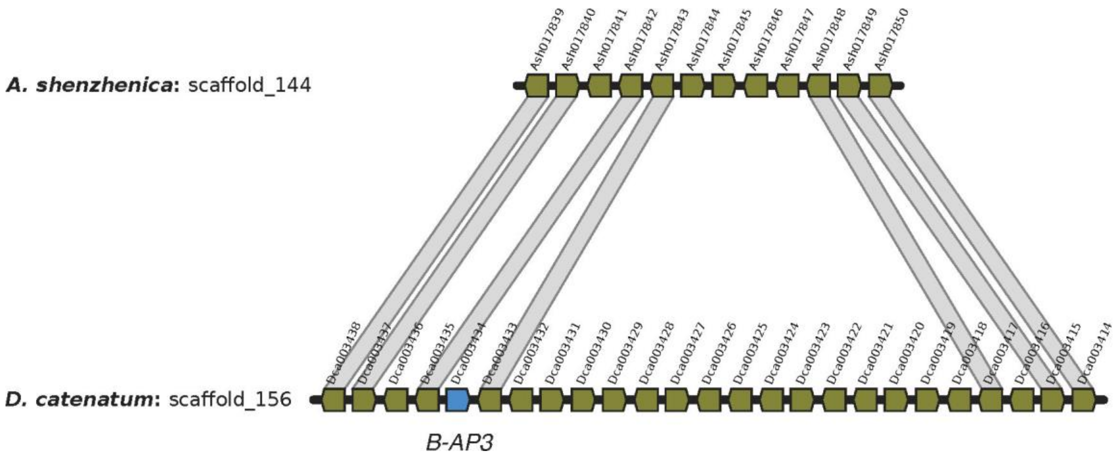

Supplementary Figure 10 | One co-linear segment between *A. shenzhenica* and *D. catenatum* showing a loss of a *B-AP3* gene in *A. shenzhenica*. The co-linearity between the two species was identified by i-ADHoRe (v3.0) using homologous pairs aligned by BLASTP (*E* value < 1 × 10−5 and c-score ⩾ 0.5). Arrowed blocks indicate genes with orientations, and grey lines connect homologues in the co-linear region. Two genes that are on both sides of one *B-AP3* gene (blue) in *D. catenatum* have two corresponding homologues that remain in the same order in *A. shenzhenica* but without any gene in between, suggesting the *B-AP3* gene got lost in *A. shenzhenica*.

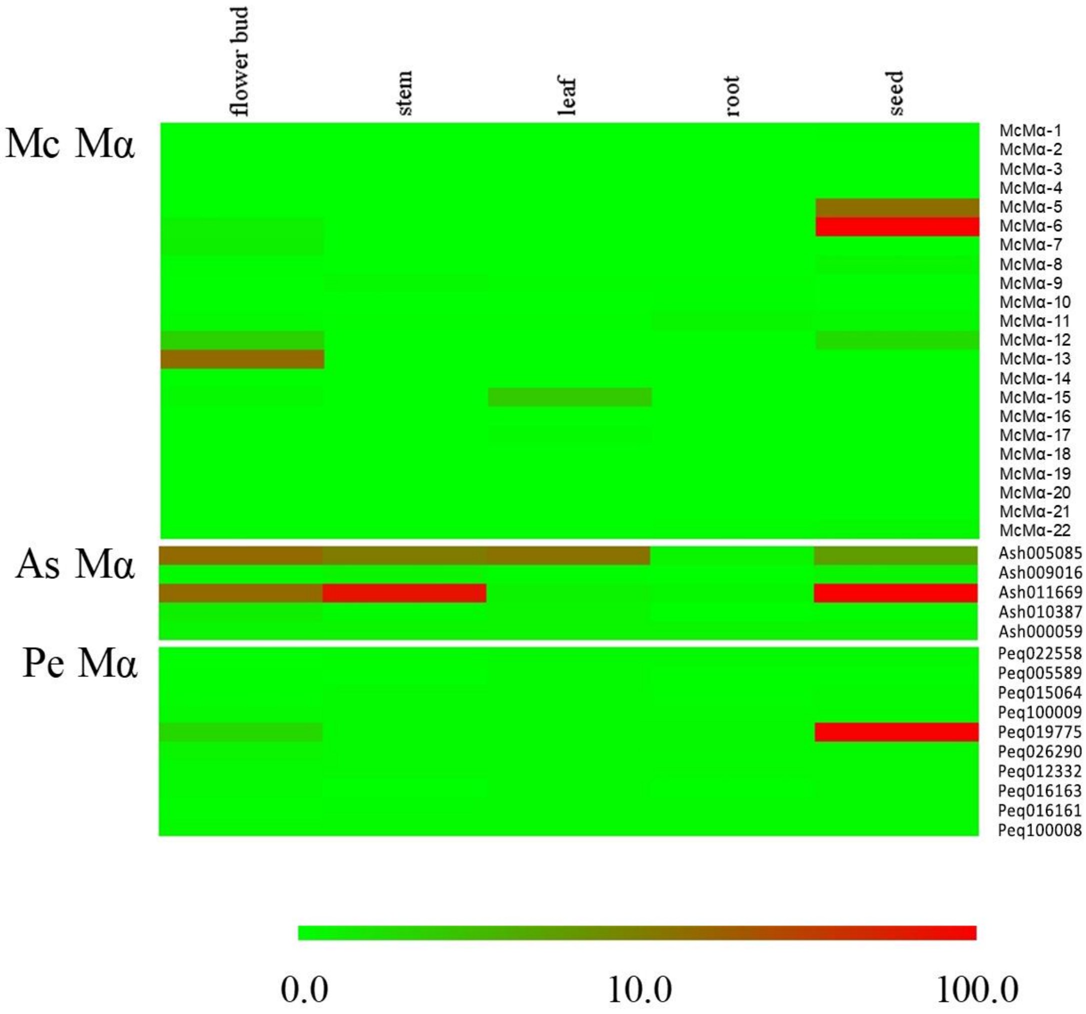

Supplementary Figure 11 | Expression of type I Mα MADS-box genes in *M. capitulata, A. shenzhenica* and *P. equestris*. As: *A. shenzhenica*; Mc: *M. capitulata*; Pe: *P. equestris*. The expression levels (FPKM value) are represented by the color bar.

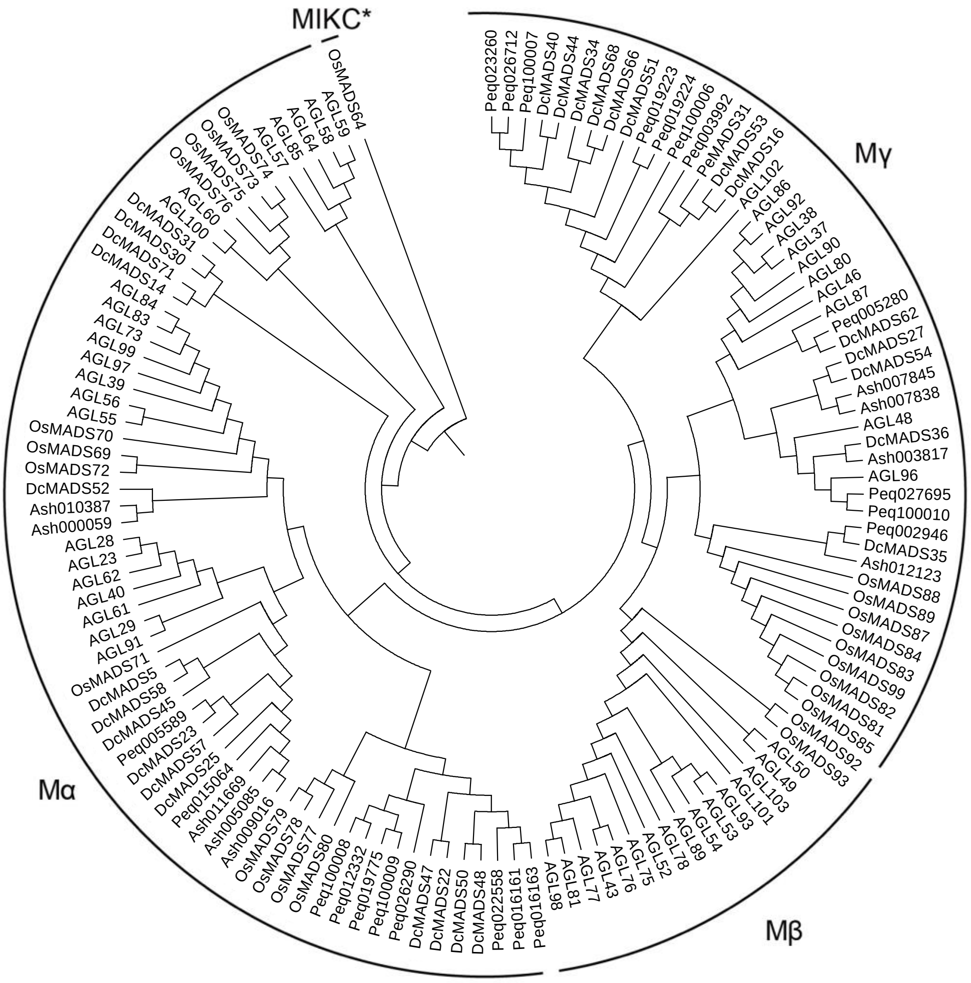

Supplementary Figure 12 | Phylogenetic tree of type I MADS-box genes.

Phylogenetic analysis shows that the genomes of *A. shenzhenica*, *P. equestris* and *D. catenatum* do not contain any type I Mβ MADS–box genes (see Mβ subclade). For gene names: Ash, *A. shenzhenica*; Peq, *P. equestris*; Dc, *D. catenatum*; Os, *O. sativa*; AGL, *Agamous-like* genes from *Arabidopsis.*

doi:10.1038/nature23897

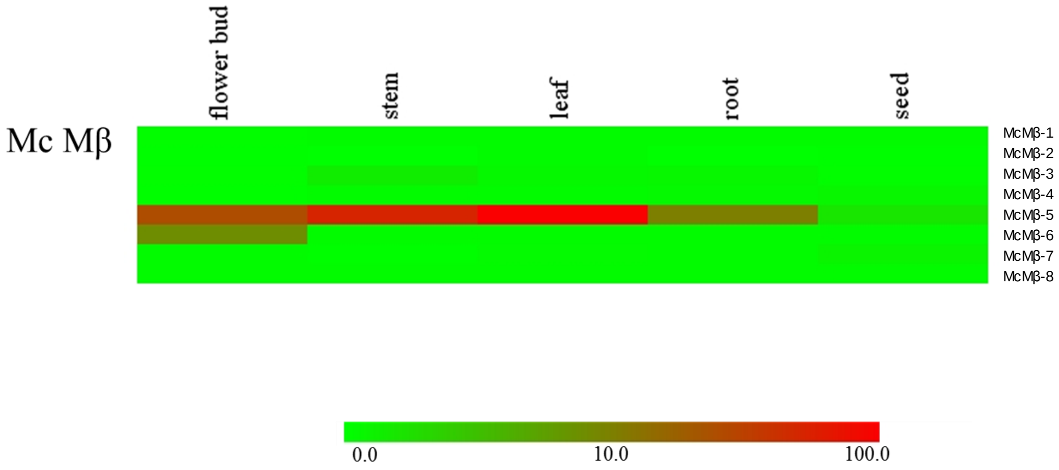

Supplementary Figure 13 | Expression of type I Mβ MADS-box genes in *M. capitulata*. The expression levels (FPKM value) are represented by the color bar.

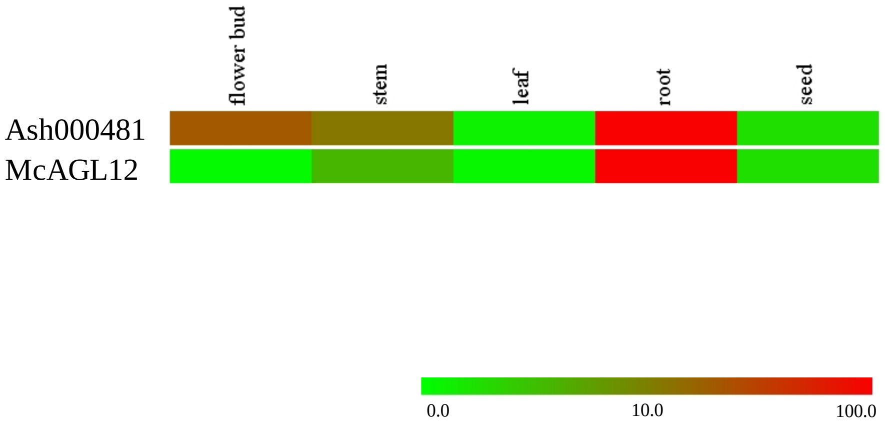

Supplementary Figure 14 | Expression level of *A. shenzhenica* and *M. capitulata AGL12* genes. *Arabidopsis AGL12* is involved in root cell differentiation and in flowering transition[26]. The expression levels (FPKM value) are represented by the color bar.

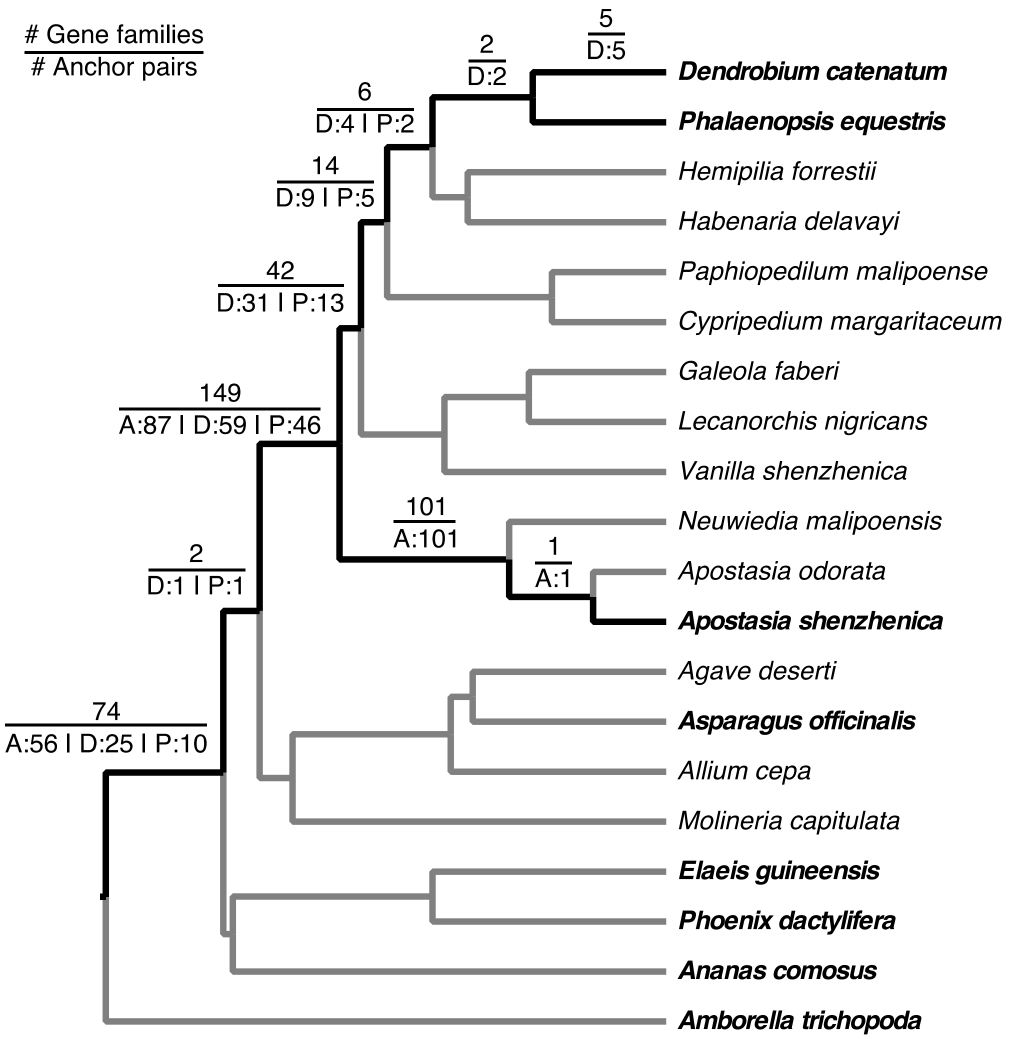

Supplementary Figure 15 | Phylogenomic analysis of orchid WGD events. The numbers on the branches of the species tree indicate the number of gene families that had one or more anchor pairs (duplicated genes found in co-linear regions) from at least one of the three orchids with genomes that coalesced on the respective branch (top), as well as the individual contributions of anchor pairs from the three orchids (bottom; A: *A. shenzhenica*; D: *D. catenatum*; and P: *P. equestris*). All the duplication events have bootstrap values over 50%.

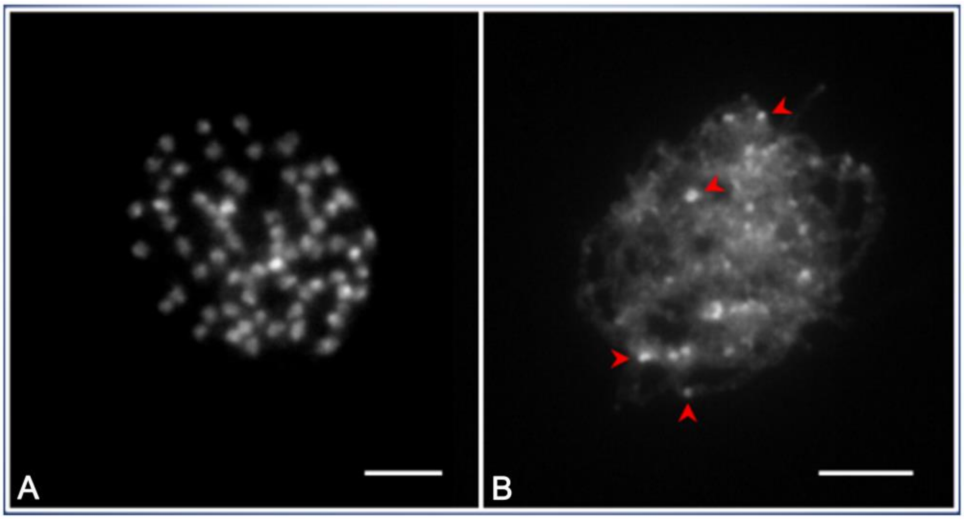

Supplementary Figure 16 | Fluorescent dye (DAPI)-stained chromosomal complements. *A. shenzhenica* presents 68 mitotic metaphase chromosomes in a root cell (A) and shows euchromatin with a few scattered heterochromatin spots (some of these are indicated by the red arrowheads) in a cell at the meiotic pachytene stage (B). The bars represent 5 μm.

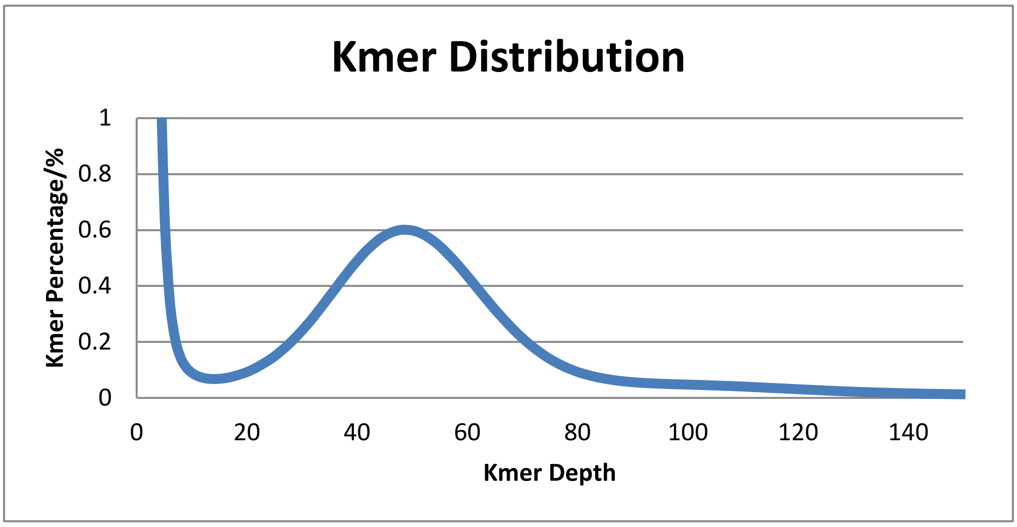

Supplementary Figure 17 | *K-mer* distribution of sequencing reads. According to the distribution, we estimate that the genome size of *A. shenzhenica* is approximately 471 Mb. The analysis is based on the Illumina data.

doi:10.1038/nature23897

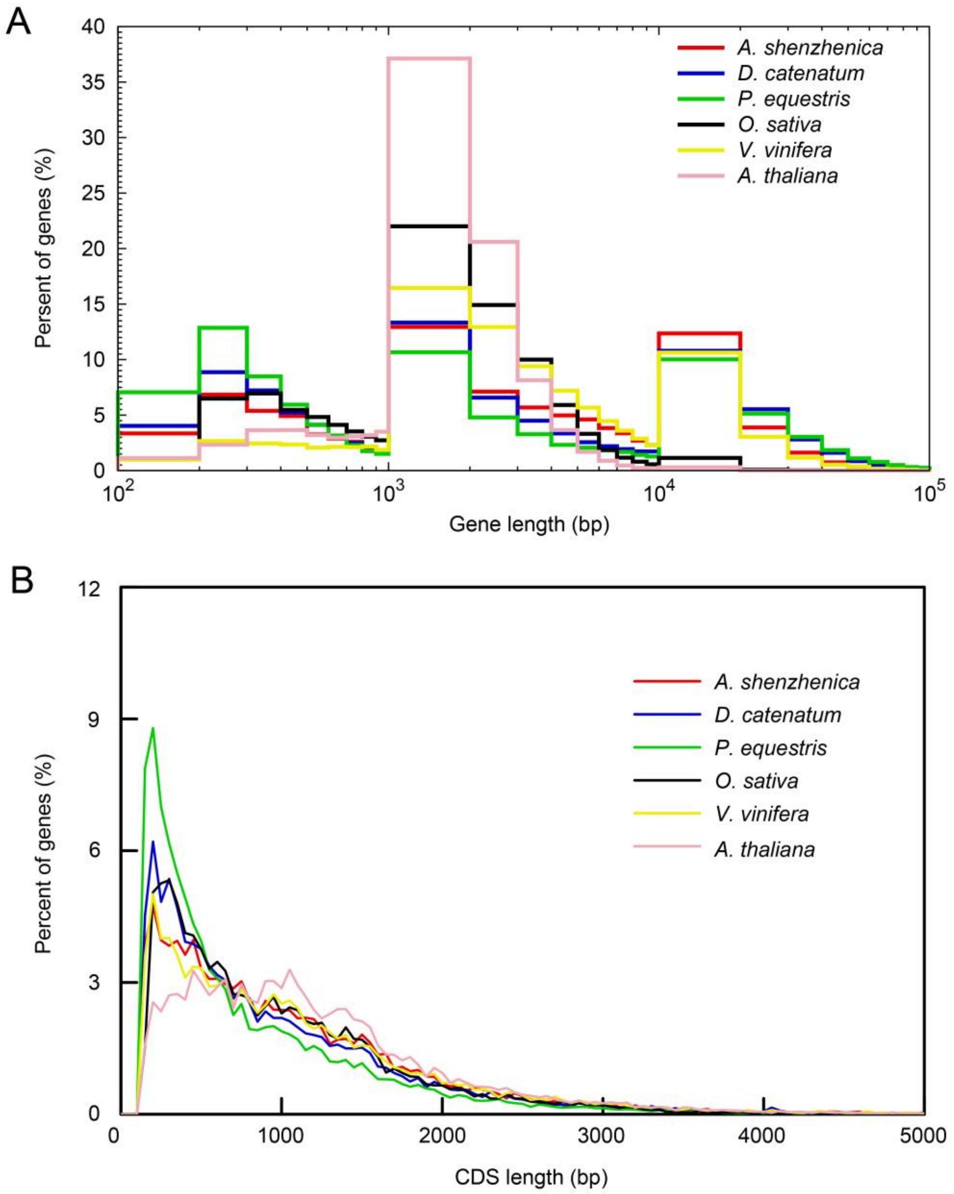

Supplementary Figure 18 | Distribution of gene length (A) and CDS length (B) for six plants.

doi:10.1038/nature23897

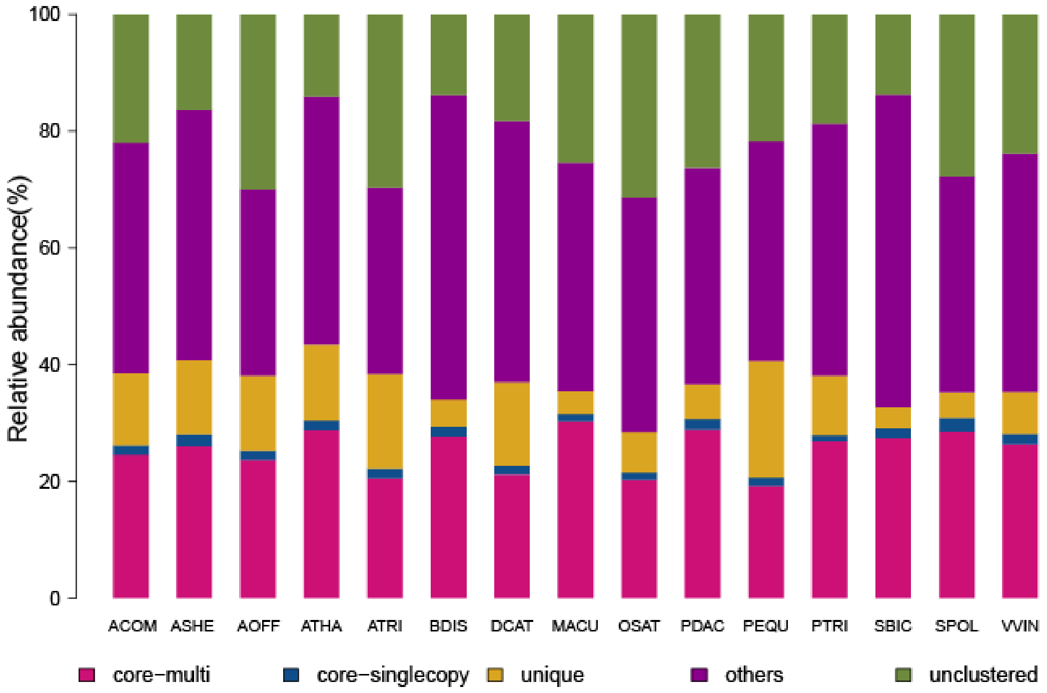

Supplementary Figure 19 | Orthologous genes found in different plant species. **Core-multi**: genes have orthologues in all other species and might have paralogues in species within one family. **Core-single copy**: genes have orthologues in all other species and no other paralogues in this species within one family. **Unique**: genes for which only one family contains genes of this species. **Other orthologues**: genes are not included in the other mentioned categories. **Unclustered genes**: genes that are unclustered into any family. ACOM, *A. comosus*; AOFF, *A. offcinalis*; ASHE, *A. shenzhenica*; ATHA, *A. thaliana*; ATRI, *A. trichopoda*; BDIS, *B. distachyon*; DCAT, *D. catenatum*; MACU, *M. acuminata*; OSAT, *O. sativa*; PDAC, *P. dactylifera*; PEQU, *P. equestris*; PTRI, *P. trichocarpa*; SBIC, *S. bicolor;* SPOL, *S. polyrrhiza;* VVIN, *V. vinifera.*

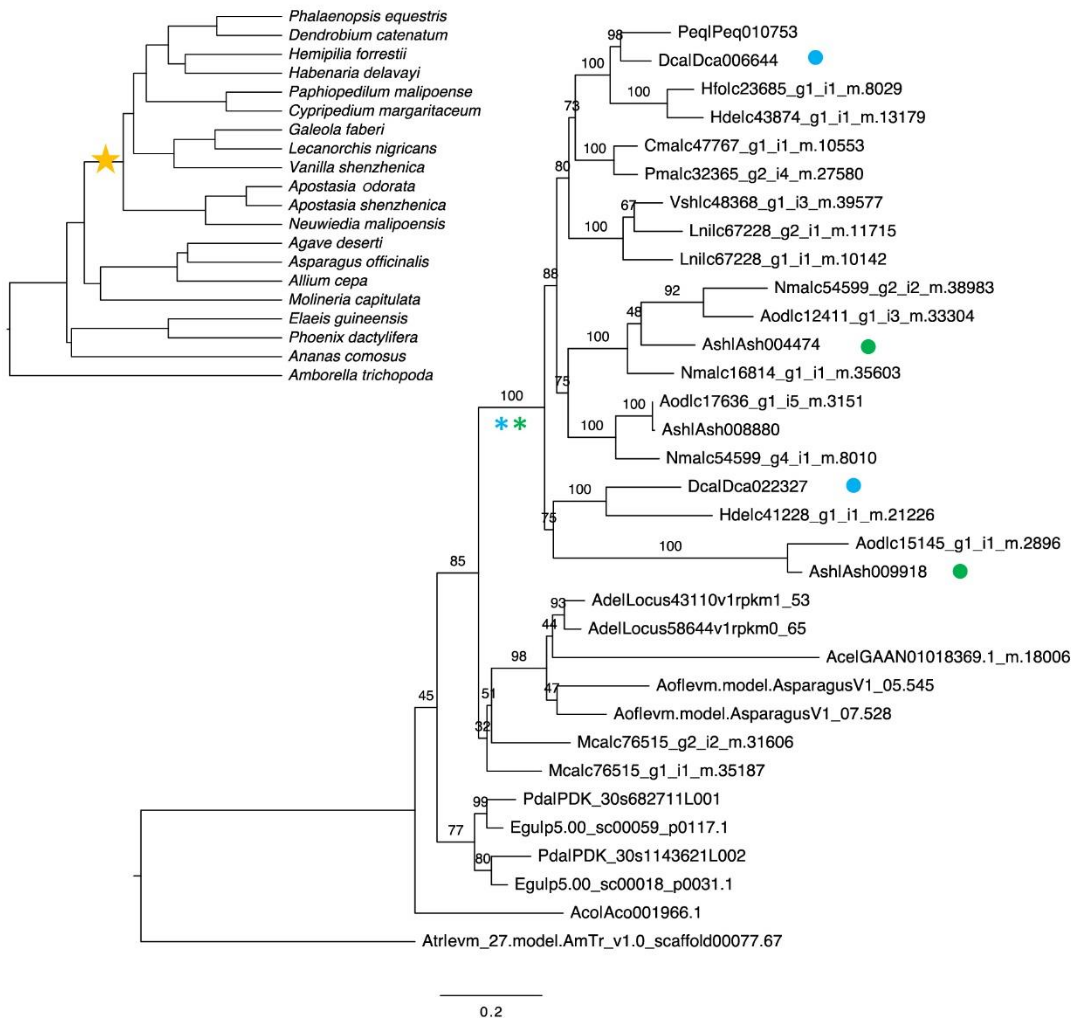

Supplementary Figure 20 | Example of coalescence points of anchor pairs mapped onto the species phylogeny. The blue and green dots in the gene tree (right) denote pairs of duplicated anchor genes from *D. catenatum* and *A. shenzhenica*, respectively. Both anchor pairs coalesce onto the same node (blue and green asterisks), which corresponds to a putative WGD event on the stem branch of the orchids in the species tree (left, yellow stars). Although two anchor pairs were found here, we count the gene family only once as support for a shared WGD event in orchids in **Figure 3** and **Supplementary Figure 14** because the anchor pairs are from different species and support the same single event. The numbers on the gene tree branches are bootstrap values (%). For gene names, the first three letters denote species: Ace, *A. cepa*; Aco, *A. comosus*; Ade, *A. deserti*; Aod, *A. odorata*; Aof, *A. officinalis*; Ash, *A. shenzhenica*; Atr, *A. trichopoda*; Cma, *C. margaritaceum*; Dca, *D. catenatum*; Egu,

E. guineensis; Gfa: *G. faberi*; Hde, *H. delavayi*; Hfo, *H. forrestii*; Lni, *L. nigricans*; Mca, *M. capitulata*; Nma, *N. malipoensis*; Pda, *P. dactylifera*; Peq, *P. equestris*; Pma, *P. malipoense*; Vsh, *V. shenzhenica*.

doi:10.1038/nature23897

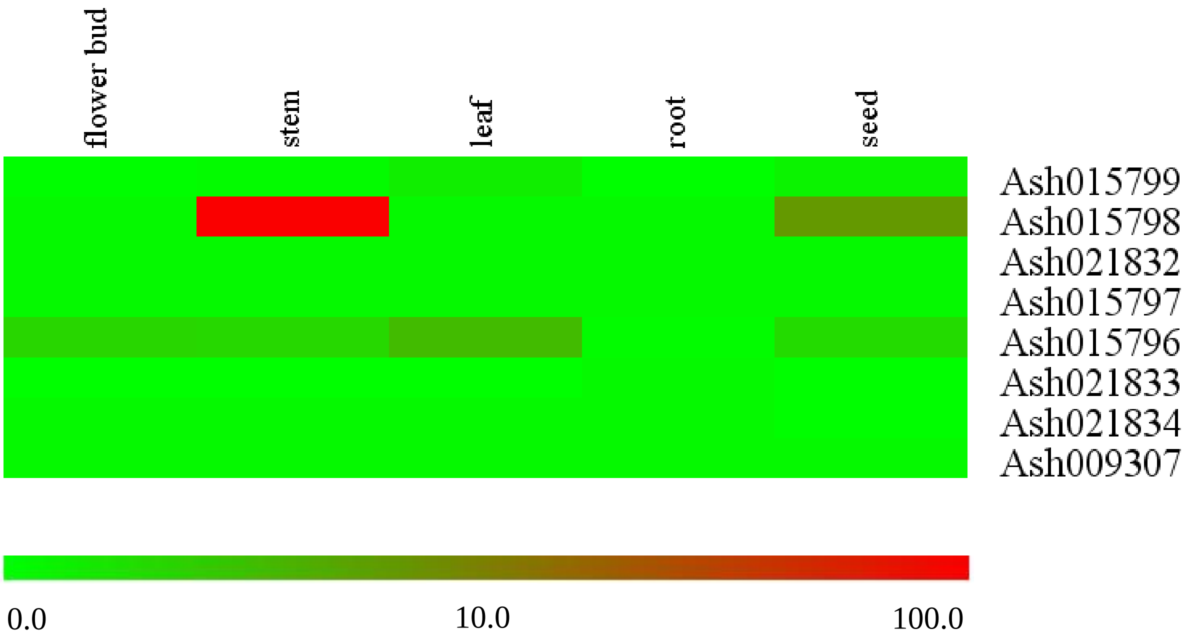

Supplementary Figure 21 | Expression of *A. shenzhenica* genes with O-methyltransferase activity. The expression levels (FPKM value) are represented by the color bar.

doi:10.1038/nature23897

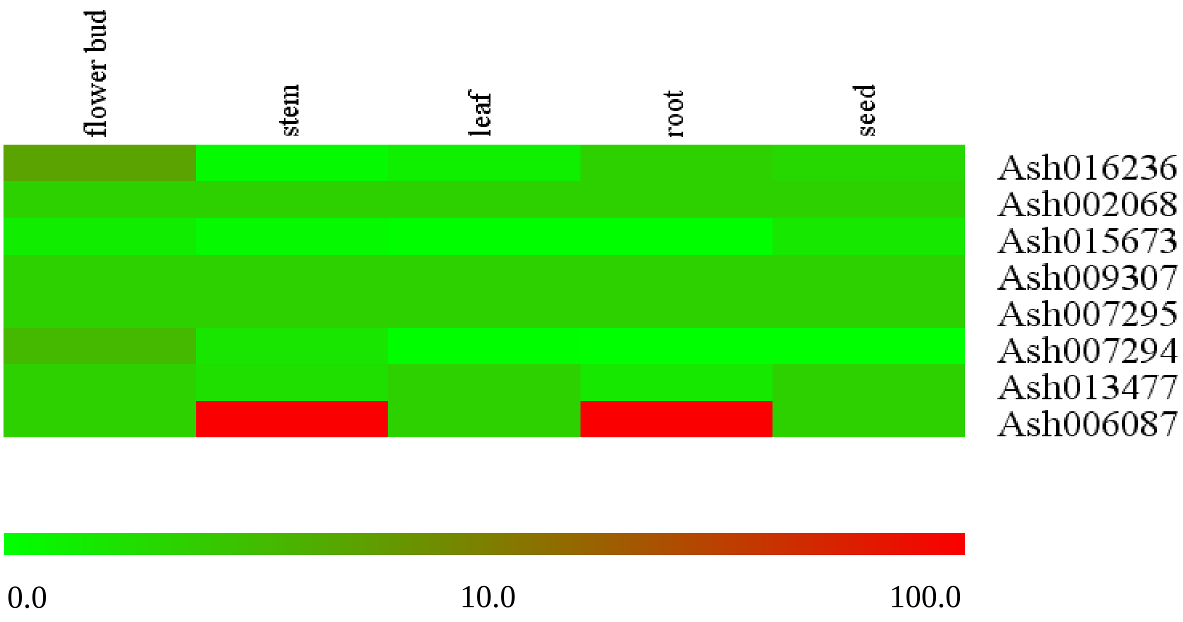

Supplementary Figure 22 | Expression of *A. shenzhenica* genes involved in flavone and flavonol biosynthesis. The expression levels (FPKM value) are represented by the color bar.

doi:10.1038/nature23897

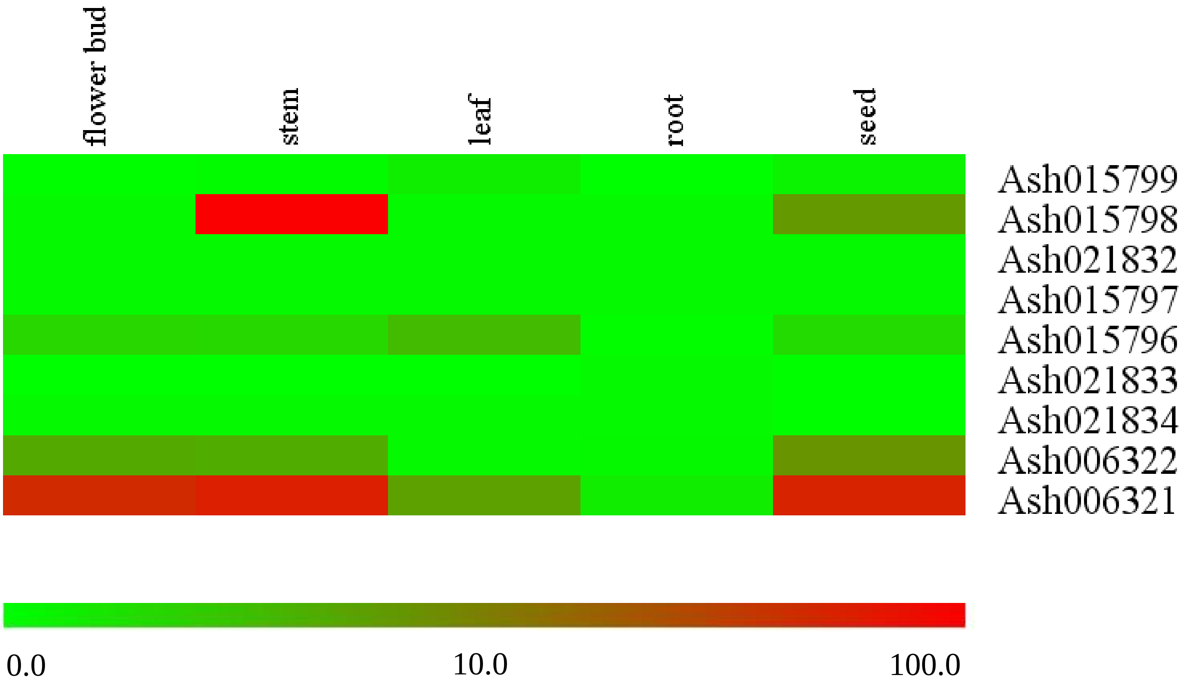

Supplementary Figure 23 | Expression of *A. shenzhenica* genes involved in biosynthesis of stilbenoids, diarylheptanoids, and gingerol. The expression levels (FPKM value) are represented by the color bar.

doi:10.1038/nature23897

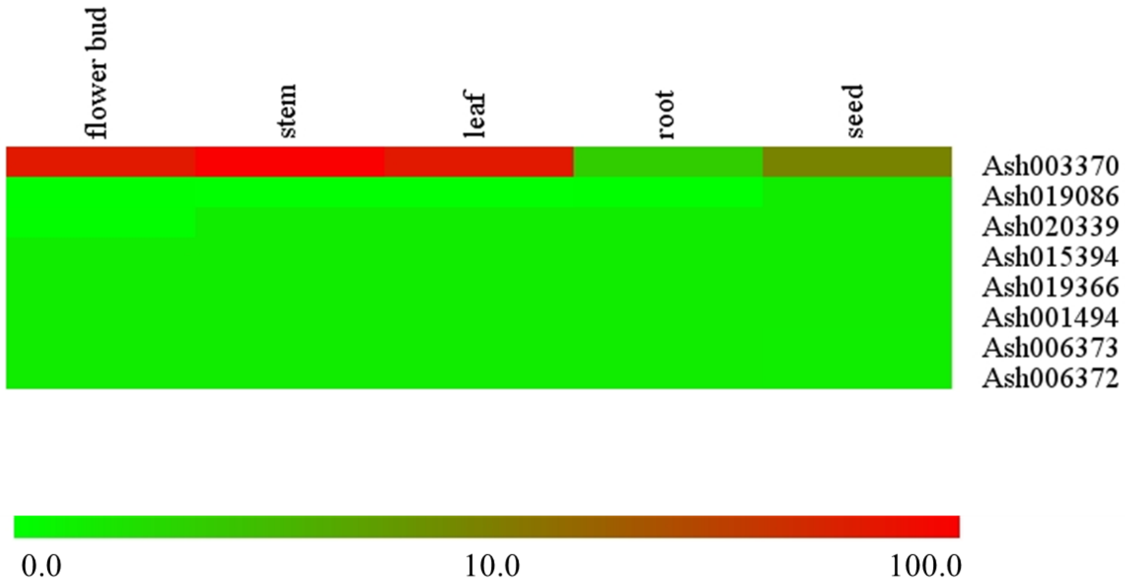

Supplementary Figure 24 | Expression of *A. shenzhenica* cysteine-type peptidase genes. The expression levels (FPKM value) are represented by the color bar.

doi:10.1038/nature23897

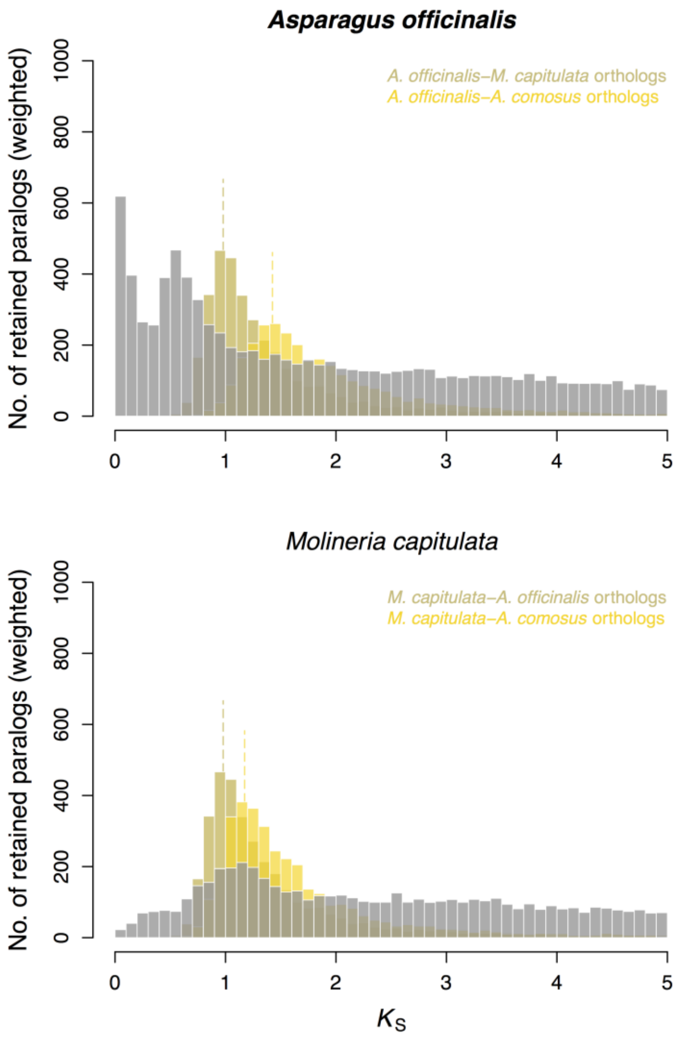

Supplementary Figure 25 | Distribution of synonymous substitutions per synonymous site (K_S) of the whole paranome for the *A. officinalis* genome and the *M. capitulata* transcriptome. K_S distributions of paralogues are shown in gray. K_S distributions of one-to-one orthologues between *A. officinalis* and *M. capitulata*, and between each of these and *A. comosus*, representing their respective times of divergence, are shown in light brown and bright yellow, respectively, with long-dashed lines indicating the peaks (based on KDE) of the distributions. The putative WGD evident from the K_S peak in *A. officinalis* does not seem to be shared with *M. capitulata*. The K_S peak in *M. capitulata* likely represents a signature of the ancient monocot τ WGD event[17, 18], shared with *A. comosus* and all other commelinids as well as Orchidaceae.

doi:10.1038/nature23897

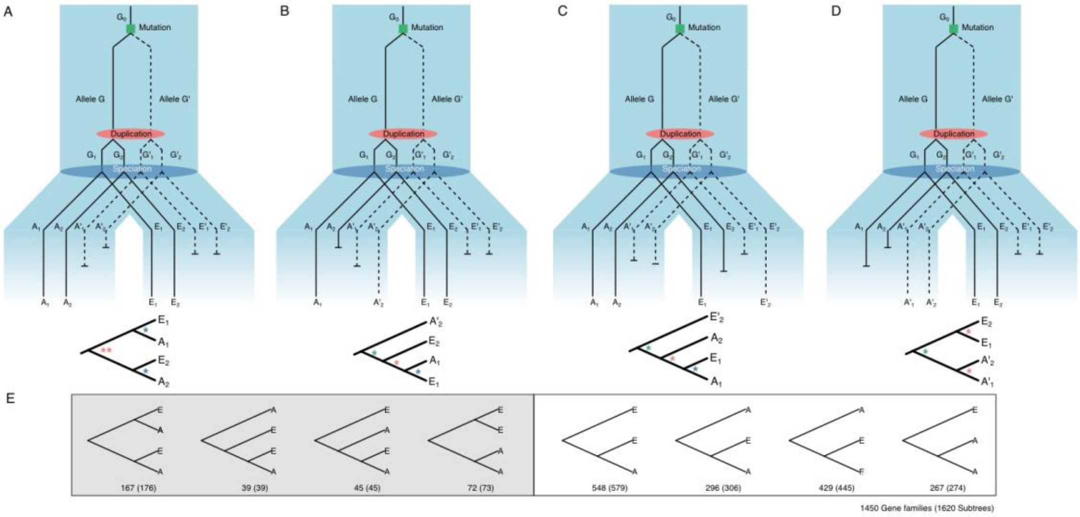

Supplementary Figure 26 | Incomplete lineage sorting underlying the gene tree discordance owing to short time intervals between the orchid-specific WGD and speciation. (A)–(D) Four possible scenarios illustrating fixation of different alleles at two paralogous loci resulting from a duplication event that occurred shortly before a speciation event (see **Supplementary Note 2.3** for details). The possible resulting gene trees are shown below for each scenario with asterisks denoting possible phylogenetic events that branch the gene trees. The colors of the asterisks indicate different phylogenetic events, with blue, red and green, for speciation, duplication and mutation/allele divergence, respectively. (E) The gene trees on the left highlighted in grey are topologies that would be expected without gene loss for each scenario above; the gene trees on the right show the expected topologies including loss or incomplete sampling of one gene. The numbers below the topologies show support from phylogenomic analysis including the three orchids, plus *A. officinalis* and *A. trichopoda* based on full gene trees found and contained subtrees (in parentheses), see **Supplementary Figure 26**. A, *A. shenzhenica*; E, Epidendroideae (including *D. catenatum* and *P. equestris*).

doi:10.1038/nature23897

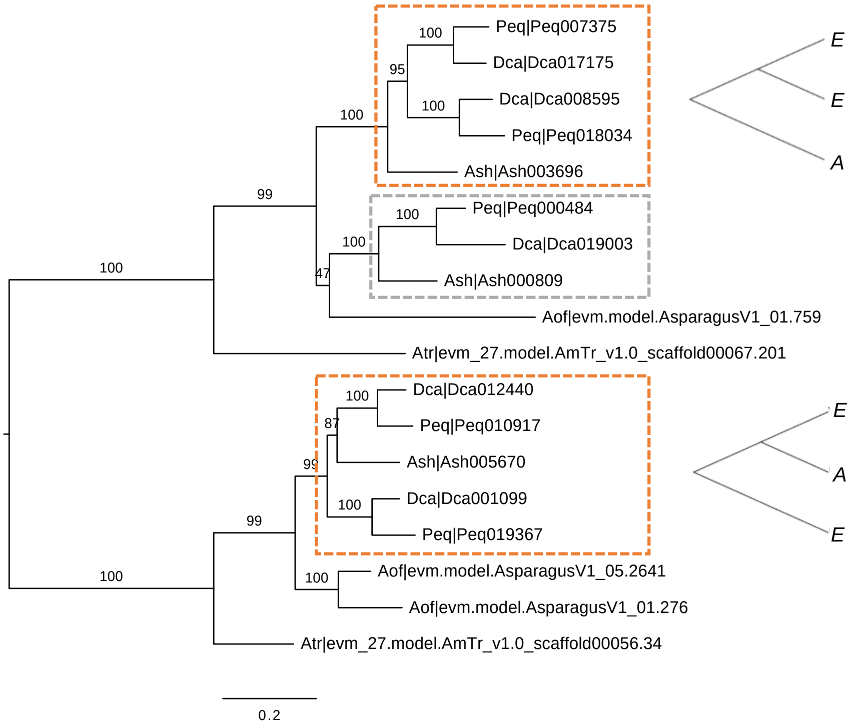

Supplementary Figure 27 | Example of the analysis of gene (sub)tree topologies. Full gene family tree with three subtrees (clades) highlighted; the highlighted subtrees each only contain genes from the three orchids (dashed rectangles). Only two of the subtrees (orange rectangles) show duplication events; one supports an independent duplication in Epidendroideae (top), and the other supports a shared duplication in orchids (bottom). The numbers on the gene tree branches are bootstrap values (%). For gene names, the first three letters denote species: Ash, *A. shenzhenica*; Atr, *A. trichopoda*; Dca, *D. catenatum*; Aof, *A. officinalis*; Peq, *P. equestris*. In the two simplified topologies, A indicates *A. shenzhenica*; E, Epidendroideae (including *D. catenatum* and *P. equestris*).

doi:10.1038/nature23897

Supplementary Tables

Supplementary Table 1 | Summary of the *A. shenzhenica* genome sequencing data derived from the Illumina technology.

Insert size(bp)	Read length(bp)	Number of reads	Total data(Gb)	Sequence depth(X)
180	90	168,223,606	15.14	34.97
500	100	275,127,442	27.51	63.54
800	90	98,419,616	8.86	20.46
2000	90	91,713,060	8.25	19.06
5000	90	119,967,670	10.80	24.94
10000	90	46,631,366	4.20	9.69
20000	125	58,492,233	5.26	12.23

doi:10.1038/nature23897

Supplementary Table 2 | Summary of the 3rd generation sequencing derived from the PacBio RS II.

Species	Number of bases	Number of reads	Mean read length (bp)
A. shenzhenica	5,441,238,461	1,352,628	4,023
D. catenatum	11,060,594,629	1,502,920	7,359
P. equestris	10,539,372,308	1,352,628	7,792

doi:10.1038/nature23897

Supplementary Table 3 | Summary of the 10X genomics Linked-Reads sequencing derived from the Illumina technology.

Species	Read length (bp)	Raw paired reads	Raw bases	Filtered paired reads	Filtered bases
A. shenzhenica	150	369,749,121	110,924,736,300	318,763,894	95,629,168,200
D. catenatum	150	415,271,158	124,581,347,400	387,320,106	116,196,031,800
P. equestris	150	411,429,315	123,428,794,500	383,649,995	115,094,998,500

doi:10.1038/nature23897

Supplementary Table 4 | Summary of the *A. shenzhenica* genome assembled by Illumina, PacBio and 10X genomics technologies.

	**Scaffold**		**Contig**	
	**Length(bp)**	**Number**	**Length(bp)**	**Number**
**max_len**	12,424,053		556,054	
**N10**	10,110,636	4	223,148	112
**N20**	6,237,011	8	166,933	283
**N30**	5,003,307	14	130,671	503
**N40**	3,457,059	22	103,308	780
**N50**	3,029,156	32	80,069	1,136
**N60**	2,413,737	45	63,275	1,590
**N70**	1,972,814	61	47,252	2,184
**N80**	1,402,703	82	31,086	3,022
**N90**	765,391	115	15,048	4,473
**Total_length**	348,734,287		322,901,144	
**GC_rate**	31.2%		33.7%	

doi:10.1038/nature23897

Supplementary Table 5 | Summary of gene annotation of *A. shenzhenica*.

Gene set		Protein coding gene number	Average gene length (bp)	Average CDS length (bp)	Average exon per gene	Average exon length (bp)	Average intron length (bp)
De novo	AUGUSTUS	26,015	8771.89	1128.15	4.68	241.14	2078.02
	GlimmerHMM	36,406	8249.20	701.52	3.39	207.17	3163.09
Homolog	*A. thaliana*	19,532	5099.79	904.39	4.02	225.25	1391.48
(exonerate)	*O. sativa*	21,804	4905.70	870.30	3.81	228.44	1436.23
	P. equestris	28,179	3899.98	775.81	3.35	231.88	1331.82
	S. bicolor	20,483	4829.59	881.21	3.93	223.95	1345.31
	Z. mays	20,929	4620.41	852.71	3.79	224.79	1348.81
RNA-seq (Cufflinks)		20,202	9588.04	1144.15	4.77	239.67	1471.21
CEGMA		448	11532.37	1225.80	8.46	144.82	1380.78
MAKER		23,181	7866.12	994.09	4.08	243.45	1915.22
Final set		21,841	7103.12	1099.99	4.51	244.07	1436.59

doi:10.1038/nature23897

Supplementary Table 6 | The assessment of assembled genomes by BUSCO.

	A. shenzhenica				*P. equestris*				*D. catenatum*			
	**Assembly**		**Gene set**		**Assembly**		**Gene set**		**Assembly**		**Gene set**	
	**Proteins**	**Percentage**	**Proteins**	**Percentage**	**Proteins**	**Percentage**	**Proteins**	**Percentage**	**Proteins**	**Percentage**	**Proteins**	**Percentage**
Complete Single-Copy BUSCOs	685	71.65%	575	60.15%	676	70.71%	553	57.85%	679	71.02%	550	57.53%
Complete Duplicated BUSCOs	210	21.97%	304	31.8%	194	20.29%	299	31.28%	205	21.44%	324	33.89%
Fragmented BUSCOs	20	2.09%	38	3.97%	33	3.45%	60	6.28%	29	3.03%	42	4.39%
Missing BUSCOs	41	4.29%	39	4.08%	53	5.54%	44	4.60%	43	4.50%	40	4.18%
Total BUSCO groups searched	956	100%	956	100%	956	100.00%	956	100.00%	956	100.00%	956	100.00%

doi:10.1038/nature23897

Supplementary Table 7 | Summary of the improved *P. equestris* and *D. catenatum* assemblies.

	P. equestris			***D. catenatum***		
	**Original assembly**	**Add PacBio and 10X genomics**	**Final assembly**	**Original assembly**	**Add PacBio and 10X genomics**	**Final assembly**
**Total length (bp)**	1,086,208,158	1,151,255,532	1,133,282,102	1,008,546,262	1,123,989,432	1,119,944,395
**Longest scaffold (bp)**	81,761,211	88,274,276	80,517,012	2,592,627	2,684,897	34,145,153
**N50 of scaffold**	523	499	192	723	822	213
**N50 length of scaffold (bp)**	359,115	408,145	1,217,477	391,462	367,633	1,055,340
**Total length of contigs (bp)**	1,002,400,532	1,090,349,635	1,045,027,212	955,235,028	1,092,146,538	1,060,791,339
**N50 of contig**	13,281	6,372	6,124	8,479	5,824	5,656
**N50 length of contig (bp)**	20,555	45,984	45,791	33,094	51,913	51,736
**Number of protein-coding genes**	29,431	-	29,545	28,910	-	29,257

Note: Final assembly is with both PacBio and 10X genomics Linked-reads Data

doi:10.1038/nature23897

Supplementary Table 8 | List of 40 MADS-box genes identified in *A. shenzhenica*.

Gene ID	Name	ORF (bp)	Protein length (aa)	Type	Subfamily	Pseudogene
Ash007825	AsMADS1	1050	349	MIKC*		
Ash005085	AsMADS2	714	237	Type I	Mα	
Ash011255	AsMADS3	684	227	MIKC^c	B-AP3	
Ash007845	AsMADS4	1167	388	Type I	Mγ	
Ash003440	AsMADS5	633	210	MIKC^c	B-PI	
Ash005080	AsMADS6	792	263	MIKC^c	Bs	
Ash004262	AsMADS7	822	273	MIKC^c	E	
Ash006974	AsMADS8	720	239	MIKC^c	AGL6	
AsMADS9	AsMADS9	216	71	MIKC^c	AGL6	✓
Ash015784	AsMADS10	738	245	MIKC^c	AGL6	
Ash018282	AsMADS11	657	218	MIKC^c	E	
Ash006741	AsMADS12	738	245	MIKC^c	E	
Ash002380	AsMADS13	447	148	MIKC^c	SQUA	
Ash015738	AsMADS14	630	229	MIKC^c	C/D	
Ash013457	AsMADS15	750	249	MIKC^c	SQUA	
Ash011284	AsMADS16	450	149	MIKC^c	C/D	
Ash002061	AsMADS17	1188	395	MIKC*		

Ash015066	AsMADS18	759	252	MIKC^c	ANR1	
Ash017092	AsMADS19	762	253	MIKC^c	ANR1	
Ash100002	AsMADS20	702	233	MIKC^c	C/D	
Ash002488	AsMADS21	879	292	MIKC^c	C/D	
Ash003251	AsMADS22	681	226	MIKC^c	B-AP3	
Ash007674	AsMADS23	705	234	MIKC^c	SOC	
Ash016527	AsMADS24	699	232	MIKC^c	SVP	
Ash010253	AsMADS25	486	161	MIKC^c	ANR1	
Ash017552	AsMADS26	696	231	MIKC^c	SVP	
Ash100001	AsMADS27	342	113	MIKC^c	ANR1	
Ash000481	AsMADS28	639	212	MIKC^c	AGL12	
Ash002504	AsMADS29	585	194	MIKC^c	OsMADS32	
Ash009016	AsMADS30	732	243	Type I	Mα	
Ash011669	AsMADS31	669	222	Type I	Mα	
Ash010387	AsMADS32	810	269	Type I	Mα	
Ash000059	AsMADS33	540	179	Type I	Mα	
Ash003817	AsMADS34	831	276	Type I	Mγ	
Ash007838	AsMADS35	660	219	Type I	Mγ	
AsMADS36	AsMADS36	204	67	MIKC*		✓
Ash015872	AsMADS37	648	215	MIKC^c	SOC	
AsMADS38	AsMADS38	492	163	MIKC^c	SVP	✓

doi:10.1038/nature23897

Ash012123	AsMADS39	756	251	Type I	Mγ	
AsMADS40	AsMADS40	357	118	MIKC^{c}		✓

doi:10.1038/nature23897

Supplementary Table 9 | Summary of repeat annotation of *A. shenzhenica*.

	RepBase TEs		TE Proteins		*De novo*		Combined TEs	
	**Length (bp)**	**%in Genome**	**Length (bp)**	**% in Genome**	**Length (bp)**	**% in Genome**	**Length (bp)**	**% in Genome**
DNA	3,604,289	1.03	3,106,810	0.89	18,995,301	5.45	22,534,396	6.46
LINE	10,240,458	2.94	9,511,200	2.73	41,316,780	11.85	44,203,442	12.68
SINE	12,411	0.00	0	0.00	149,789	0.04	161,345	0.05
LTR	10,644,451	3.05	15,167,007	4.35	72,767,333	20.87	76,930,066	22.06
Other	5,732	0.00	0	0.00	0	0.00	5,732	0.00
Unknown	38,684	0.01	0	0.00	20,482,533	5.87	20,520,835	5.88
Total	24,555,914	7.04	27,699,462	7.94	137,241,384	39.35	146,653,786	42.05

doi:10.1038/nature23897

Supplementary Table 10 | Length distribution of gene elements in twelve sequenced plants.

Species	Protein coding gene number	Average gene length (bp)	Median gene length (bp)	Average CDS length (bp)	Median CDS length (bp)	Average exon per gene	Average exon length (bp)	Median exon length (bp)	Average intron length (bp)	Median intron length (bp)	Average intergenic region length (bp)	Median intergenic region length (bp)
A. shenzhenica	21,841	6137.69	2518.00	1099.28	876.00	4.50	244.17	138.00	1438.65	403.00	9531.45	6177.00
P. equestris	29,545	8373.90	1185.00	841.32	570.00	3.56	236.05	150.00	2937.72	381.00	24903.93	12783.00
D. catenatum	29,257	7816.87	1757.00	1011.00	756.00	3.85	262.52	148.00	2387.03	329.00	22639.53	10503.50
Z. mays	39,815	3361.45	1824.00	1091.32	915.00	4.51	241.77	134.00	646.06	152.00	49040.89	23226.50
S. bicolor	27,160	2942.00	2165.00	1260.98	1095.00	4.85	259.90	136.50	436.44	145.00	22363.18	5336.00
O. sativa	35,402	2177.38	1479.50	998.58	810.00	3.80	262.69	142.00	420.79	150.00	9459.07	4625.00
P. heterocycla	31,987	3099.24	2373.00	1210.22	981.00	5.28	229.03	132.00	440.95	209.00	35884.38	21907.50
A. thaliana	26,637	1909.57	1593.00	1242.78	1065.00	5.23	237.50	134.00	157.54	98.00	2569.35	1223.00
V. vinifera	25,328	6129.29	3160.00	1177.49	918.00	6.12	192.54	123.00	967.97	208.00	13026.83	5015.00
A. officinalis	27,375	6835.52	3385.00	1004.02	732.00	5.03	199.52	124.00	1446.29	361.00	34806.10	13301.00
A. comosus	27,024	4341.80	2903.00	1171.28	927.00	5.53	211.68	123.00	699.41	295.00	9286.57	4375.00
A. trichopoda	25,933	5761.95	1308.00	962.18	669.00	4.16	231.31	136.00	1519.05	397.00	20684.78	11242.00

Note: We considered the start and stop codons as the two boundaries for calculating gene length.

doi:10.1038/nature23897

Supplementary Table 11 | Gene function annotation of *A. shenzhenica*.

		Number	Percent (%)
Total		21,841	
Annotated	InterPro	14,693	67.27
	GO	10,499	48.07
	KEGG	10,283	47.08
	SwissProt	11,578	53.01
	TrEMBL	18,277	83.68
	NCBI non-redundant	18,243	83.52
Unannotated		3,449	15.79

doi:10.1038/nature23897

Supplementary Table 12 | Summary of ncRNA annotation of *A. shenzhenica*.

Type		Number	Average length (bp)	Total length (bp)	% of genome
miRNA		43	125.56	5,399	0.00155
tRNA		203	74.75	15,174	0.00435
rRNA	rRNA	452	162.68	73,530	0.02109
	18S	35	771.83	27,014	0.00775
	28S	26	192.96	5,017	0.00144
	5.8S	11	151.73	1,669	0.00048
	5S	380	104.82	39,830	0.01142
snRNA	snRNA	93	103.60	9,635	0.00276
	CD-box	45	98.42	4,429	0.00127
	HACA-box	0	0.00	0	0.00000
	splicing	48	108.46	5,206	0.00149
	scaRNA	0	0.00	0	0.00000

doi:10.1038/nature23897

Supplementary Table 13 | Information about the transcriptomes used in this study.

Family	Subfamily	Species	Tissues used in genome annotation	Tissues used in expression analysis	Tissues used in WGD and phylogenetic analysis	Number of predicted proteins	Number of predicted proteins with plant homologs
Orchidaceae	Apostasioideae	*Apostasia shenzhenica*	Flower bud, leaf, root, seed, stem, tuber	flower bud, pollen, stem, root, leaf, seed			
		Apostasia odorata			flower bud	23,504	18,030
		Neuwiedia malipoensis			flower	25,211	23,011
	Vanilloideae	*Vanilla shenzhenica*		flower bud, pollinia, stem, root, leaf	floral bract, lip, sepal, column, leaf, node with leaf and root, aerial root	25,767	18,188
		Lecanorchis nigricans			flower	19,529	16,608
		Galeola faberi			flower	19,093	16,969
	Cypripedioideae	*Paphiopedilum malipoense*		flower bud, pollinia, stem, root, leaf	flower bud	24,360	20,631
		Cypripedium margaritaceum			flower, leaf	22,803	16,485
	Orchidoideae	*Hemipilia forrestii*		flower	flower	20,641	18,295
		Habenaria delavayi			leaf	20,260	17,842
	Epidendroideae	*Phalaenopsis equestris*		flower bud, pollinia, stem, root, leaf, seed			
		Dendrobium catenatum		flower bud, pollinia, stem, root, leaf			
Hypoxidaceae		*Molineria capitulata*		flower bud, pollen, stem, root, leaf, seed	flower bud	26,430	19,865

doi:10.1038/nature23897

Supplementary Table 14 | Summary of orthologous gene families in 15 sequenced plant species.

Species	Genes	Unclustered genes	Clustered genes	Familys	Unique families	Unique families genes	Common families	Common families genes	Single Copy	Average genes per family
A. comosus	27,024	5,950	21,074	13,279	936	3,346	4,120	7,079	439	1.587
A. shenzhenica	21,841	3,573	18,268	11,995	562	2,789	4,120	6,121	439	1.523
A. officinalis	27,375	8,220	19,155	12,014	901	3,521	4,120	6,920	439	1.594
A. thaliana	26,637	3,750	22,887	12,719	859	3,466	4,120	8,108	439	1.799
A. trichopoda	25,933	7,699	18,234	12,200	1,044	4,206	4,120	5,758	439	1.495
B. distachyon	26,415	3,655	22,760	15,344	421	1,240	4,120	7,748	439	1.483
D. catenatum	29,257	5,339	23,918	14,050	1,036	4,183	4,120	6,638	439	1.702
M. acuminata	34,241	8,710	25,531	12,865	538	1,359	4,120	10,792	439	1.985
O. sativa	35,402	11,106	24,296	16,352	958	2,473	4,120	7,604	439	1.486
P. dactylifera	23,890	6,281	17,609	11,011	444	1,431	4,120	7,331	439	1.599
P. equestris	29,545	6,420	23,125	13,752	1,197	5,887	4,120	6,112	439	1.682
P. trichocarpa	40,984	7,683	33,301	14,471	1,362	4,181	4,120	11,440	439	2.301
S. bicolor	27,160	3,723	23,437	15,749	361	984	4,120	7,893	439	1.488
S. polyrrhiza	18,357	5,095	13,262	10,076	264	797	4,120	5,672	439	1.316
V. vinifera	25,328	6,032	19,296	12,808	643	1,833	4,120	7,113	439	1.507

Unique families = families present only in one species

doi:10.1038/nature23897

Supplementary Table 15 | The GO term enrichment of *A. shenzhenica* lineage significantly contracted gene families.

GO ID	GO Term	GO Class	P value	Adjusted P value	x1	x2	n	N	GO level
GO:0005507	copper ion binding	MF	1.69E-18	9.95E-17	9	74	18	21841	7
GO:0003824	catalytic activity	MF	2.61E-12	1.54E-10	18	4970	18	21841	2
GO:0008152	metabolic process	BP	9.77E-12	5.76E-10	18	5347	18	21841	2
GO:0005488	binding	MF	1.38E-10	8.16E-09	18	6194	18	21841	2
GO:0004713	protein tyrosine kinase activity	MF	3.13E-10	1.85E-08	9	597	18	21841	7
GO:0055114	oxidation-reduction process	BP	9.01E-10	5.31E-08	9	673	18	21841	3
GO:0006468	protein phosphorylation	BP	9.12E-10	5.38E-08	9	674	18	21841	6
GO:0016491	oxidoreductase activity	MF	4.06E-09	2.39E-07	9	799	18	21841	3
GO:0046872	metal ion binding	MF	9.87E-08	5.82E-06	10	1582	18	21841	5
GO:0005524	ATP binding	MF	2.15E-07	1.27E-05	9	1265	18	21841	8
GO:0005515	protein binding	MF	1.14E-05	0.00067253	9	2031	18	21841	3
GO:0044238	primary metabolic process	BP	0.0005568 37	0.03285341 2	10	4120	18	21841	3

Note: N: total gene number; n: gene number in the list; x1: gene number with a GO term in the list; x2: gene number with a GO term in total.

doi:10.1038/nature23897

Supplementary Table 16 | The GO term enrichment of *A. shenzhenica* lineage significantly expanded gene families.

GO ID	GO Term	GO Class	P value	Adjusted P value	x1	x2	n	N	GO level
GO:0006259	DNA metabolic process	BP	4.44E-40	2.93E-38	38	702	65	21841	5
GO:0003964	RNA-directed DNA polymerase activity	MF	3.37E-36	2.22E-34	29	322	65	21841	7
GO:0006278	RNA-dependent DNA replication	BP	3.37E-36	2.22E-34	29	322	65	21841	7
GO:0003723	RNA binding	MF	1.95E-28	1.28E-26	29	593	65	21841	4
GO:0015074	DNA integration	BP	3.37E-23	2.22E-21	18	177	65	21841	6
GO:0044249	cellular biosynthetic process	BP	3.61E-18	2.38E-16	32	1796	65	21841	4
GO:0016740	transferase activity	MF	2.60E-17	1.71E-15	32	1921	65	21841	3
GO:0044237	cellular metabolic process	BP	1.94E-16	1.28E-14	41	3758	65	21841	3
GO:0003676	nucleic acid binding	MF	3.22E-16	2.12E-14	30	1783	65	21841	3
GO:0044238	primary metabolic process	BP	5.37E-15	3.54E-13	41	4120	65	21841	3
GO:0008152	metabolic process	BP	2.29E-13	1.51E-11	44	5347	65	21841	2
GO:0003824	catalytic activity	MF	5.12E-08	3.38E-06	35	4970	65	21841	2
GO:0004144	diacylglycerol O-acyltransferase activity	MF	5.00E-07	3.30E-05	3	6	65	21841	8
GO:0005488	binding	MF	4.34E-06	0.000287	36	6194	65	21841	2
GO:0045017	glycerolipid biosynthetic process	BP	7.81E-05	0.005158	3	28	65	21841	5
GO:0046914	transition metal ion binding	MF	9.27E-05	0.006116	12	1110	65	21841	6
GO:0006979	response to oxidative stress	BP	0.000326	0.021548	3	45	65	21841	4
GO:0004601	peroxidase activity	MF	0.000446	0.029449	3	50	65	21841	3

Note: N: total gene number; n: gene number in the list; x1: gene number with a GO term in the list; x2: gene number with a GO term in total.

doi:10.1038/nature23897

Supplementary Table 17 | The KEGG Pathway enrichment of *A. shenzhenica* lineage significantly contracted gene families.

MapID	MapTitle	P value	Adjusted P value	x	y	n	N
map00053	Ascorbate and aldarate metabolism	5.53E-17	4.42E-16	8	57	18	21841
map04626	Plant-pathogen interaction	2.09E-10	1.67E-09	8	364	18	21841
map01100	Metabolic pathways	9.33E-06	7.46E-05	9	1982	18	21841

Note: N: total gene number; n: gene number in the list; x: gene number with a KEGG term in the list; y: gene number with a KEGG term in total.

Supplementary Table 18 | The KEGG pathway enrichment of *A. shenzhenica* lineage significantly expanded gene families.

MapID	MapTitle	P value	Adjusted P value	x	y	n	N
map03440	Homologous recombination	1.23E-31	1.36E-30	20	104	65	21841
map03008	Ribosome biogenesis in eukaryotes	6.19E-13	6.81E-12	12	199	65	21841
map03018	RNA degradation	1.64E-12	1.80E-11	12	216	65	21841
map00073	Cutin, suberine and wax biosynthesis	0.000266	0.002924	3	42	65	21841
map03015	mRNA surveillance pathway	0.00097	0.010668	4	147	65	21841
map00360	Phenylalanine metabolism	0.001194	0.013139	3	70	65	21841

Note: N: total gene number; n: gene number in the list; x: gene number with a KEGG term in the list; y: gene number with a KEGG term in total.

Supplementary Table 19 | GO term enrichment of *A. shenzhenica*-specific gene families.

GO ID	GO Term	GO Class	P value	Adjusted P value	x1	x2	n	N	GO level
GO:0003964	RNA-directed DNA polymerase activity	MF	3.24E-05	0.010411	67	322	2789	21841	7
GO:0006278	RNA-dependent DNA replication	BP	3.24E-05	0.010411	67	322	2789	21841	7

Note: N: total gene number; n: gene number in the list; x1: gene number with a GO term in the list; x2: gene number with a GO term in total.

Supplementary Table 20 | GO term enrichment of Orchidaceae-specific gene families.

GO ID	GO Term	GO Class	P value	Adjusted P value	x1	x2	n	N	GO level
GO:0008171	O-methyltransferase activity	MF	3.91E-07	9.90E-05	8	28	568	21841	6
GO:0008234	Cysteine-type peptidase activity	MF	4.45E-05	0.011261	6	26	568	21841	7

Note: N: total gene number; n: gene number in the list; x1: gene number with a GO term in the list; x2: gene number with a GO term in total.

Supplementary Table 21 | KEGG pathway enrichment of Orchidaceae-specific gene families.

MapID	MapTitle	P value	Adjusted P value	x	y	n	N
map00945	Stilbenoid, diarylheptanoid and gingerol biosynthesis	7.75E-07	3.87E-05	12	78	568	21841
map00944	Flavone and flavonol biosynthesis	5.79E-05	0.0028937	7	39	568	21841

Note: N: total gene number; n: gene number in the list; x: gene number with a KEGG term in the list; y: gene number with a KEGG term in total.

doi:10.1038/nature23897

Supplementary Table 22 | GO term enrichment of monocot-specific gene families.

GO ID	GO Term	GO Class	P value	Adjusted P value	x1	x2	n	N	GO level
GO:0000156	two-component response regulator activity	MF	5.43E-06	0.000521	3	20	38	21841	4
GO:0015299	solute:hydrogen antiporter activity	MF	1.23E-05	0.00118	3	26	38	21841	8
GO:0015298	solute:cation antiporter activity	MF	1.23E-05	0.00118	3	26	38	21841	7
GO:0000160	two-component signal transduction system (phosphorelay)	BP	1.54E-05	0.001483	3	28	38	21841	4
GO:0015300	solute:solute antiporter activity	MF	1.91E-05	0.001833	3	30	38	21841	7
GO:0004871	signal transducer activity	MF	7.96E-05	0.007643	3	48	38	21841	3
GO:0060089	molecular transducer activity	MF	7.96E-05	0.007643	3	48	38	21841	2
GO:0015078	hydrogen ion transmembrane transporter activity	MF	0.000188	0.018061	3	64	38	21841	9
GO:0015297	antiporter activity	MF	0.000245	0.023559	3	70	38	21841	6
GO:0015291	secondary active transmembrane transporter activity	MF	0.000364	0.034939	3	80	38	21841	5

Note: N: total gene number; n: gene number in the list; x1: gene number with a GO term in the list; x2: gene number with a GO term in total.

Supplementary Table 23 | KEGG pathway enrichment of monocot-specific gene families.

Map ID	Map Title	P value	Adjusted P value	x	y	n	N
map04075	Plant hormone signal transduction	0.000748	0.007479	5	418	38	21841
map03020	RNA polymerase	0.004582	0.045821	2	58	38	21841

Note: N: total gene number; n: gene number in the list; x: gene number with a KEGG term in the list; y: gene number with a KEGG term in total.

doi:10.1038/nature23897

Supplementary Data

Supplementary Data 1 | The protein sequences of *A. shenzhenica* MADS-box genes.

>Ash005085

MATVGKKRQSMGRQKIPMKRIEREEARQVCFSKRRVGLFKKATELSILCGAEIGIVVFSPAGKP
FSFGHPSLDYIIKRFAGGDRIRGVGPMLAAQGIDCGALQGLNINHARLTEELEEQRRRKEVLEA
SMEALRSSDHRRPLPWEADVHEMGMEELESFQKALEVLWRNVASRREEIEIMMEVEYWRPAA
APVAYGGGGYAGSCNGLYLTPPSLVPGGGGLADGFCSCGIERAYFWG

>Ash007845

MRASFRPLGFLCSMAASSPAAKWTAREDKEFERALATFGEQTPNRWERVAAAVGGGKTAAD
VGRHYQLLLDDIDSIESGRVPFPNYRTPQAPATTAGRGGGAAAAILSHDCHRSPVPAMARKKIS
LAYITNEATRRATLKKRRRGLMKKVRELSVLCGVSACAVVYAPPDPKPEVWPSTEEAAEILRKF
CCMPDVDKARKMMNQESFLLQRQNKLQEQLRRLTHENDELAAANLLRQCLAGRPASTLCREE
IFVLLCLIERRSKAVQVRLDQLRAQAERFGSSPEIYLPGGQSPPPLPLPAPLMLPAPLPLQVKDF
KDPIGFDSAAIEAMRRCDWLSGGVSGDHAPEVTGGELQIMPYAQGPVAPGCASASGGFDDML
AMFIDPAIYI

>Ash009016

MMTVRKKRQSMGRQKIPMKRIESEEARQVCFSKRRSGLFKKATELSILCGAEIGIVVFSPAGKP
FSFGHPSLDYIIKRFAGGDGTRGMGPMGAAQGIDYGALQELNINHARLTEELEEQRRRKEVLE
AAIEALRCGDHRRALPWEADVHEMGIEELESFQRALEVLWRSVASRREEIEIMMEVETKAAVAA
AAAAAATPMAYGGGGYVGGCNGLYLTPPSLLPGGGVAGGFYSRGLERPYFWG

>Ash011669

MKRIESEEARQVCFSKRRSGLFKKATELSILCGAEIGIVVFSPAGKPFSFGHPSLDYIIKRFAGGD
RIRGVGPMSTAHGIDCGSLQDLNINHARLTEELEEQRRRKEALEVAMEALRSSDHRRALPWKA
DVHEMEMEELESFQKVLEVLWGNVASRREEIEIMMMEVETRAAAAAAAAAAAAPVAYGYVGS
CNGLYLTPPSLVPDLTPPSLVPAGCISRLHP

>Ash010387

MQFTNGNNYISTQKSPKIKPTNRMHLIPLDFPLYRSSPSIKEEPPSLLLIFLLLLLLLLLLMSAINVIA
RRKRKASSGRRKIEIKKIENEDARHVSFSKRRCGLFTKACDLATLCGVELAIVVYSAAGNPYSFG
SPTVDSVVDRFLAGRPDRVEPGRMNQAGLVQVLNQQCMDLAAQIRAAEERRSSLELRIRAAG
ESNLLSQLTNDEEDASPMQLEKLKKLLLDLRRKVDYRFADLLSGKEAIEFNSNLTSSQLVELDVV
GGWPGYEL

>Ash000059

MGRQKIEIKKIENEDARLVSFSKRRSGLFNKASCLTTLCGVDAAVVVFSPAGTPYSFGSPRVEM
VVDRFLSSGREMEEPNFFRQHHATSVQELNRRCMELENQVEAAQARRRALEERLSAVAAVRD
EPEEMEVEELEALKEALNQLKKMTDEHMKERAAGSSSSAAQNEAGPSTPLDLG

>Ash003817

MARKKVTLAWICNDATRRATLKKRRRGLMKKVKELSILCDVRACAIVYSPHETQPEVWPSVAE
AARILARFRSMPEIEQSKKMLDQESFLRQRVARLHDQLNRLQRENHELEAAAVLRECLAGSSLL
DLRIEELASLAWMLEHKAKVVQERIDQHLILAAAAPELEMPPMEGAGAEVDAGPLQPPGPPPP
PPPPPPLLSLIGDGKFIPCHEGEFRWIPGGSGGHEAYSGAAAGPAPVGERDYFGGGGGGGEV
AELGLLDHHDKQQSGWENCFFHLY

>Ash007838

MARKKISLAYITNEATRRATLKKRRRGLMKKVRELSVLCGVSACAVVYAPPDPKPEVWPSTEE
AAEILRKFCCMPDVDKARKMMNQESFLLQRQNKLQEQLRRLTHENDELAAANLLRQCLAGRP
ASTLCRKEIFVLLCLIERRSKAVQVRLDQLRAQAERFGSSPEIYLPGGQSPPSLPLPAPLMLPAP
SPLQVKDFTDPIGFDSAAIEAMLLCDWLS

>Ash012123

MARKKVKLEWIANDSARRATLKKRRKGLLKKASELSTLCDVKACLILFAAGEPQPEVWPSHHE
AARILAKLKRLPEMEQGKKMMNQEGFMRQRITKLQEQLQKQERENRELETTLLMYQGLMGKT
MSDVSIKDLTSLAWLIELKVNQVRKRLDALRKNATPPPQQQQPAAAAAAVAVKKENASPQLPP
AAAEKTALEVAMEELQKQDWFSEIMMNPLAAHEEMMGHHLSFLELAGSGFLGGAWPDAYFPV
N

>Ash007825

MGRVKLKIKRLENTSGRQVTYSKRRAGILKKAKELSILCDIDILLLMFSPTEKPTLCLGERSNIDDII
TKFAQLTPQERAKRELESLEALKKTFKKLDHDVNIQDFLGSSQTVEDLTNHSRALQAQISEVER
KLSCWTDPEKVNSIDQVRQMEETLRESLNRIQMQKEYLGKQLVSLDCSGQFPNGLNLPMALN
NDIQSSHMQWFYDNNGQNLMLLQDSSFLHQRNRECSTDTMLQGYPGFFSTEKQAGYCKNGT
DESLNELSQNSCLQLQLGAQYPYQPYGLNMLGEKKFGPDGEVSLQEDHIHLQVNGFEQPKPG
YDAGTQNWASISCSCGVGMFDDQLCPQQQNQT

>Ash011255

MGRGRIEIKKIENPTNRQVTYSKRRLGIMKKAKELSVLCDAHVSLLMFSSTGKLADYCSPSTNIK
GIIERYQHATGVDLWNAQYERMQETMKQLKEVNQSLRKEIRQRTGEDVEGLEMMELRGLEQSI
EESLRIVRQRKYHVITTQTDTYKKKLKSTKEANWALMHELHMKGEIPQYDLVAEDPTSVYDSTM

SMVNVVPRMFAFNVIHPNQQSLQGIDYDSHDLSLR

>Ash003440

MGRGKIEIKRIENSTNRQVTFSKRRNGIMKKAREISVLCDAQVSLVIFSSLGKMSEYCSPSTTLS
KMLERYQQNSGKKLWDAKHENLSAEIDRIKKENDNMQIELRHLKGEDVNSLNPKELIPIEEALQ
NGLASVREKQMDFLKMLKKKEMMLEEENKRLTYLLHHQQLAMEGSMRDLDISYHQKDREFAS
QMPMTFRLQPIQPNLQGNK

>Ash005080

MGRGKIEIKRIENPTNRQVTFSKRRGGLLKKAHELAVLTDAQVGLVVFSTSGKMYDYCSPASS
MREIIERYLKATNAHFDQDHSSQQQLYCEITRIRHETDRLQATIKQFTGEELSGLTINDLNQLED
HLEFSVNKVRFRKHQLLHQQLENLRRKVVNIFVVRHGAQEHILEDQNNYLCRALAEHHVAMEQ
QQAADHYEENKAREMGFLHEPSSLIGNFLSSVADDSAGNLLQLGPQVHGFQLQPTQPNLQDP
ALHGYALQLW

>Ash004262

MVRVREREKEREREREREREEKEKLEKKGKMGRGRVELKRIENKINRQVTFAKRRNGLLKKAY
ELSVLCDAEVALIIFSNRGRLFEFCSSSSMTQTLERYQTCSYNAGESMVPSKETQNTYQEYLKL
KARVEYLQRSQRNLLGEDLGQLSTKELEQLENQVEMSLKQIRSTKTQLMLDQLCDLKRKEQML
QEANKALKKKLQEDGPEIPLQLSWPSGSGSRGSGEHEPQSDAFFQPLTCDPSLQIGYTPLCTD
QRLNAGLSTHNINGFIPGWM

>Ash006974

MGRGRVELKRIENKINRQVTFSKRRNGIMKKAYELSVLCDAEVALIIFSCRGKLFEFGSPDITKTL
ERYKRCTFTPQAIDPNDHETLNWYQELSKLKAKYESLQRSQRHLLGEDLDELSLKELQQLERQ
LELSLTQARQKRTLLMLDQMEEIQIKERHLGDINKQLKHKLEIEGGSMRAIQGSSWLQLDNEAL
SSCSRARDPGPTLEIGRYHHYAPTGREAAAGPRNGASGNFMPGWAP

>Ash015784

MGRGRVELKRIENKINRQVTFSKRRNGLLKKAYELSVLCDAEVALIVFSSRGKLYEFGSAGTSK
TLERYQSCCYNSQATNNVVARDTQNWYQEVTKLKAKFESLQRSHRHLLGEDLGPLSVKELQQ
LERQLESALSQARQRKTQIMLDQMEELRKKERQLGEINKQLKMKLEAGGGSLRLMQGPWEPE
AVPEDHHAFQLHLSHSTAMECDPSLHIGYHQFVPPEAAMPRSNDGDQNNSFMLGWML

>Ash018282

MGRGRVELKRIENKINRQVTFAKRRNGLLKKAYELSVLCDAEVALIIFSNRGKLYEFCSSSSMLK
TLERYQKCNYGAPEANIISRETQSSQQEYLKLKARVEALQRSQRNLLGEDLGPLSSKELEQLER
QLDASLKQIRSTRTQNMLDQLADLQRREQMLCEANKALKRRLEESSQTNPQQVWDPSTAHAV
GYGRQAAQAHGDAFYHPLECEPTLQIG

>Ash006741

MGRGRVELKRIENKINRQVTFAKRRNGLLKKAYELSVLCDAEVALIIFSNRGKLYEFCSSSSMLR
TLERYQKCNFGAPETNIISRETQSSQQEYLKLKTRVEALQRSQRNLLGEDLGPLSSIDLEQLER
QLDASLKQIRSTRTQHMLDQLADLQRKEQMLCEANRSLRRRLEEASCLENPPESWNNPNSAQ
AVVYGRQTIHTQADDFYHPLVCEPTLQMGYHSDITMASATVPSVGNFVLPGWLA

>Ash002380

MGRGRVQLKRIENKINRQVTFSKRRSGLLKKAHEISVLCDAEVALIVFSNKGKLYEYSTDSSME
RILERYERYSYTERALFSNEADPQANWCLEYNKLKARVESLQKSQRHLMGEQLDSLSVKEIQH
LEQQLESSLKHIRSKKTQLLIDSISELQRKENILQEQNKNMEKEIIAKEKAKSLAQNASFEQQNQS
QYSSSSPHVLVSVSAPTCISRYGGQISEASQTSKQQ

>Ash015738

MGRGRVEIKRIENTTNRQVTFCKRRNGLLKKAYELSVLCDAEIALIIFSSRGRLYEYANHSVKGTI
ERYKKASSDNSNAGSISEANSQYYQQEASKLRQQITNLQNSNRNLMGEALSNMNAKDLKQLE
TRLEKGISKIRAKKNELLYAEIEYMQKREIELQNDNAYLRNKIADNESIQQHQHHMNLLPSTSNE
YDILPPFDISRNFLQVNLMDPSHQYSHQQQTALQLG

>Ash013457

MGRGRVQLKRIENKINRQVTFSKRRAGLLKKAFEISVLCDIEVALIVFSTKGKLYEYSTDSSMERI
LERYERHCYAGKGLMSSQPELQEDMCQEYGKLKSKVEALQRSQSHMMGEKLDSLSTKELQQI
EQKLEIALELIRLRKNQSLLDTITELQCKERSLIEQNSVLEKKILQIDKARRMTNQIQWEQQTQAQ
PCSSPSTFLLNDTLPILNIGQYPARIGAAQEDNSAQPLGRRNGNGLPQWMLRSPT

>Ash011284

MGRGKIEIKRIENTTNRQVTFCKRRNGLLKKAYELSVLCDAEVALIIFSSRGRLYEYANNSVKGTI
ERYKKASTDISNTGSFSEANSLYYKQEASKLHQQITNLQNSNRNLMGDAVSNMNIRELRQLEK
RLEASINKIRSKKNELLYAEIEYLQKRDVELNNDYMYLRNKIADNERAQQHQHMNILPSSSTEYE
VMPTFDSRNSLQVHVMDPNSHHSRQQLTALQLGYII

>Ash002061

MHRLYSSMSSRTLGAYARPTVEVQFSVHRQRPSAVAGKLQIKRIENSVNRQVTFSKRRNGLIK
KAYELCVLCDIDIALIMFSPSHRLSLFSGRRRIEDVLAKYINLPEHDRGGSIVQNKEYLLRTLEKLK
CESDMAAQINKFFEPDPMTMTSLSALEPCEKFLMETLSRVNDRKKYLLSSHLAGVSACDPSASI
QMYLQPHQEGLPSVFNNELVQWVPDGDSNSNQPLFVNSDHLLSLSSNAPCVHAGRVKVATR
GLLACRSGHGGCTVGLCSHAGRSSMVTIRKSLTLVCAMRSTAKGRRKKGDNGMYNVISSAASI
PVDPLVASDTWHQAYASTELLSALIPSPPFPLIQNSLAAVELPPAEGAASCPHVPGEDDGASRA
EAYESNSGPANVG

>Ash015066

MGRGKIVIRRIDNSTSRQVTFSKRRNGLLKKARELAILCDAEVGLVIFSSTGRLYDFSSTSMKSVI
ERYTKAKEQQVTNTTNDVRFWKEEATNLRQQLQNLQENHRKLMGEELSGLSFKDLQTLENRL
EMSLRGVRMKKEQILSDEIQELNRKGYLIHQENMELYKKVNLLRQENMELYKKVFGSRDSNGE
VINSSIIPLAIGMNEESSAAFSLELCQPQGPQQPINATQPHDPELGLQLHQGKIQRESSFA

>Ash017092

MKGISDLLLALHCRSRGRKGRGMGRGKIAIRRIDNSATRQVTFSKRRNGLLKKARELAILCDAE
VGLIVFSSTGRLYDFTNTSMKSVIERYNKAKEDHGVSPNANSEIKVWQKEAVSLRQQLHNLQE
NHRQLMGENISQLSAKELQHLENQLETSLRAIRLKKEEVLSDEVQELIHKVNLVHQENTELHKTI
NISTKENMDLCKKLRGQAGVSNSTGLLRPCSEDLILFEICQPKKQTRVVETDKLNLELCLH

>Ash100002

MGRGKIEIKRIESTTNRQVTFCKRRNGLLKKAYELSVLCDAEVALIVFSSRGRLYEYSNNSIRSTI
ERYKKACADSASNSNSVVEVNSYEASKLRHQIQILQNANRHLMGDSLSSLSIKELKQLESRLER
GITRIRSKKHEMLFAEIEYMQKRETELQTENMYLRTKMAENERAQEANIVANAAALGTLPTFDS
RNYYHVNMLEAEASYPHSQDQTALHLGYDGKVDQTHNVI

>Ash002488

MEIGPGRCASSCARSTRPIGTCERDLSPVAVASCVRVYLQNKIVIANEPFGPQKNQKNPPVLFC
GSSPKLKERSMGRGKIEIKRIENTTNRQVTFCKRRNGLLKKAYELSVLCDAEIALIVFSSRGRLY
EYSNNSIKATIERYKKACADSSNSGSLVEVNSHQYYLQESAKLRHQIQLLQNANRHLMGDGLS
SLTVKELKQLENRLERGITRIRSKKEVELQNDNMYLRAKIAETEREQQANILQAGADFALPTFDS
RSYYHVNMLEAASYTHHQEQTALHLGYESKTDPGS

>Ash003251

MGRGKIEIKKIENPTNRQVTYSKRRAGIMKKARELTVLCDAEVSLIMFSSTGKFSEYCSPSADTK
SMFDRYQQVSAINLWSAQYEKMQNTLKHLKDINDELRKEIRHRMGEDLDGLDIKELRGLEQNM
DEALKIVRNRKYHVITTQTDTFKKKLKNSEETHKGLLRELEMNEEQAMFGYVEDDPSSSYEGAL

doi:10.1038/nature23897

ALANGGPHIYAFRVQPSQPNLHGMGCRSHDLRLA

>Ash007674

MVRGKTEMRRIENSTSRQVTFSKRRNGLLKKAFELSVLCDAEVGLIVFSPRGKLYEFASSSMQ
KTIERYKQRAKDMCGNGRATDPLNLEQWKLDAEALAKKTEVLEISKRKLMGEDLESCSVEELR
KLEDQLEQSLSKIRRRKNSLLSEQVEELKEKEKILLEENSLLQKQYSRLLSSASCKEAVIPQEEN
EHSKEVETELYIGWPERGRNQQLMNSKGEESVIDSDVQTRGKL

>Ash016527

MAREKIKIRKIDNATARQVTFSKRRRGLFKKAQELSILCDAEVGLIIFSSTGKLFQYSSSSMKDVI
ERRGLHSKNIQQNPDYPSIDLQLDGNADTARLSKQAAELSVQLRNMRGEDIDKLTVEELQKIES
SLELGLNRVMEKKNQQIIEQINDLQQKGVKLMEENARLRHRVGMAWAGRSATNDSENAENTV
FEDGQSSECISDISHLGGPQELNENDNISLKLGLPCSHWK

>Ash010253

MGRGKIVIRRIDNSTSRQVTFSKRRNGLLKKAKELAILCDAEVGLVIFSGTGRLYDYSSTSMKSV
FDRYKKAKGESHMTTASEARIWQEEAANLRQQLQNLQENQRKLTGEELSGLSVKDLQNLENR
LEMSLRGVRMKKEQILKDEIQELNRKGYLVHQENLELYKKLNLIQENLELYKKVLNKS

>Ash017552

MAREKIQIRKIHNATARQVTFSKRRRGLFKKAEELAILCDAEVGLIVFSATGKLFEFSSSSLSEIIE
KHKMHSKDPPKPNQPSLDLQLENGNHAMLSKQVQEATRQLRNMRGEDLQGLNIEELQKLEKT
LETGLSHVLSKKGQQIMDQINHLRKKGMQLLAENKRLRQQMAEISKMGKQVVADSDRVNYEE
GQSSESVTNLTHAGPPHESHDNDYSSDTSLRLGLSCPGWK

>Ash100001

MGRGKIAIRRIDNSASRQVTFSKRRTGLLKKAKELSILCDAEVGLIVFSGAGRLYEFASSRFVILP
RSHSPTSPSLSLSLSLSLSLSIYIYIYIYTYNIYFYSIYTYYIYIYN

>Ash000481

MARGKVQMRRIENPVHRQVTFCKRRAGLLKKAKELSVLCDADIGIIIFSTHGKLYELATNGTMR
DLIERYRQSCGETQNVGDSTQPKEITQEITSLKNEIDLLQKGLRHMLGKADVGYVTLEELHALEK
HLEIWMNYVRSAKMQLMFQEIQTLKNKEGVLKATNELLQEKIIEQTGHFDIAHIIEDNGFFTMTP
NIADIPYPLTIQNTLYQI

>Ash002504

MWVIMGRGRMEIKKIENPTQRQSTFYKRRDGLFKKARELAVLCDVDLLLLLFSSSGKLYHYHSP
TVPNVQELIERYEKAVEKKVLEDGSGDERREMEEVGRLCEALEREMRFMQVDEKEDYSLADL
QMVERNLEAAINKVRGEMETKIHKEMEKLHDMVKQRQQEGYSLCEELSHGSQHEEQESEELD
LKLGFD

>Ash015782

MVRGRTVLRRIENSASRQVAFSKRRNGLLKKAFELSVLCDAEVGVIVFSPRGKLYEFASSSMQ
ETLKRYKIHARDLCSNSRASEEGTQQRMHEVTVVAENIDLLEDSKRKLMGENLESCSWNELHE
LEDQMERGLRNIRGRKNQLLEEQVEQLKDWERQLQEENALLQKQRKVPLLHLKAQELTAAQE
DNLHVDVETDLYIGCPGRGRTEQLQEE

Supplementary References

1. De Bie, T., Cristianini, N., Demuth, J. P. & Hahn, M. W. CAFE: a computational tool for the study of gene family evolution. *Bioinformatics* **22**, 1269–1271 (2006).

2. Ibrahim, R. K. *et al.* Enzymology and compartmentation of polymethylated flavonol glucosides in *Chrysosplenium americanum*. *Phytochemistry* **26**, 1237–1245 (1987).

3. Mouradov, A. & Spangenberg, G. Flavonoids: a metabolic network mediating plants adaptation to their real estate. *Front. Plant Sci*. **5**, 620, doi: 10.3389/fpls.2014.00620 (2014).

4. Duan, D. *et al*. Genetic diversity of stilbene metabolism in *Vitis sylvestris*. *J. Exp. Bot.* **66**, 3243–3257 (2015).

5. Schnee, S., Viret, O. & Gindro, K. Role of stilbenes in the resistance of grapevine to powdery mildew. *Physiol. Mol. Plant Pathol.* **72**,128–133 (2008).

6. Lu, H. *et al*. Subfamily-specific fluorescent probes for cysteine proteases display dynamic protease activities during seed germination. *Plant Physiol.* **168**, 1462–1475 (2015).

7. Zhang, D. *et al*. The cysteine protease CEP1, a key executor involved in tapetal programmed cell death, regulates pollen development in Arabidopsis. *Plant Cell* **26**, 2939–2961 (2014).

8. Cai, J. *et al.* The genome sequence of the orchid *Phalaenopsis equestris*. *Nat. Genet.* **47**, 65–72 (2015).

9. Zhang, G.-Q. *et al*. The *Dendrobium catenatum* Lindl. genome sequence provides insights into polysaccharide synthase, floral development and adaptive evolution. *Sci. Rep.* **6**, 19029; doi: 10.1038/srep19029 (2016).

10. Vanneste, K., Baele, G., Maere, S. & Van de Peer, Y. Analysis of 41 plant genomes supports a wave of successful genome duplications in association with the Cretaceous−Paleogene

boundary. *Genome Res*. **24**, 1334–1347 (2014).

11. Ramírez, S. R. *et al*. Dating the origin of the Orchidaceae from a fossil orchid with its pollinator. *Nature* **448**, 1042–1045 (2007).

12. Gustafsson, A. L. S., Verola, C. F. & Antonelli, A. Reassessing the temporal evolution of orchids with new fossils and a Bayesian relaxed clock, with implications for the diversification of the rare South American genus *Hoffmannseggella* (Orchidaceae: Epidendroideae). *BMC Evol. Biol.* **10**, 177, doi: 10.1186/1471-2148-10-177 (2010).

13. Chen, S. C. *et al*. Networks in a large-scale phylogenetic analysis: reconstructing evolutionary history of Asparagales (Lilianae) based on four plastid genes. *PLoS ONE* **8**(3), e59472 (2013).

14. Chomicki, G. *et al*. The velamen protects photosynthetic orchid roots against UV-B damage, and a large dated phylogeny implies multiple gains and losses of this function during the Cenozoic. *New Phytol.* **205**, 1330–1341 (2014).

15. Givnish, T. J. *et al*. Orchid phylogenomics and multiple drivers of their extraordinary diversification. *Proc. R. Soc. B* **282**, 1553; doi.org/10.1098/rspb.2015.1553 (2015).

16. Givnish, T. J. *et al*. Orchid historical biogeography, diversification, Antarctica and the paradox of orchid dispersal. *J. Biogeogr*. **43**, 1905–1916 (2016).

17. Jiao, Y. N., Li, J. P., Tang, H. B. & Paterson, A. H. Integrated syntenic and phylogenomic analyses reveal an ancient genome duplication in monocots. *Plant Cell* **26**, 2792–2802 (2014).

18. Ming, R. *et al*. The pineapple genome and the evolution of CAM photosynthesis. *Nat. Genet.* **47**, 1435–1442 (2015).

19. Liu, Y. *et al*. Functional conservation of MIKC*-type MADS box genes in *Arabidopsis* and

rice pollen maturation. *Plant Cell* **25**, 1288–1303 (2013).

20. Kwantes, M., Liebsch, D. & Verelst W. How MIKC* MADS-box genes originated and evidence for their conserved function throughout the evolution of vascular plant gametophytes. *Mol. Biol. Evol.* **29**, 293–302 (2012).

21. Honys, D. & Twell, D. Transcriptome analysis of haploid male gametophyte development in *Arabidopsis*. *Genome Biol.* **5**, R85, doi: 10.1186/gb-2004-5-11-r85 (2004).

22. Verelst, W., Saedler, H. & Münster, T. MIKC* MADS-protein complexes bind motifs enriched in the proximal region of late pollen specific Arabidopsis promoters. *Plant Physiol.* **143**, 447–460 (2007).

23. Adamczyk, B. J. & Fernandez, D. E. MIKC* MADS domain heterodimers are required for pollen maturation and tube growth in *Arabidopsis*. *Plant Physiol*. **149**, 1713–1723 (2009).

24. Wolter, M. & Schill, R. Ontogenie von pollen, massulae und pollinien bei den orchideen. *Tropische und Subtropische Pflanzenwelt* **56**, 1–93 (1986).

25. Dressler, R. L. *Phylogeny and Classification of the Orchid Family* (Portland, OR: Discorides Press, 1993).

26. Tapia-López, R. *et al*. An *AGAMOUS*-related MADS-box gene, *XAL1* (*AGL12*), regulates root meristem cell proliferation and flowering transition in Arabidopsis. *Plant Physiol.* **146**, 1182–1192 (2008).
