# Supplementary figures and images for: The Apostasia genome and the evolution of orchids
Source: Nature. 2017 Sep 13;549(7672):379–83. doi: 10.1038/nature23897 (PMC7416622; doi:10.1038/nature23897)

## Slide 1
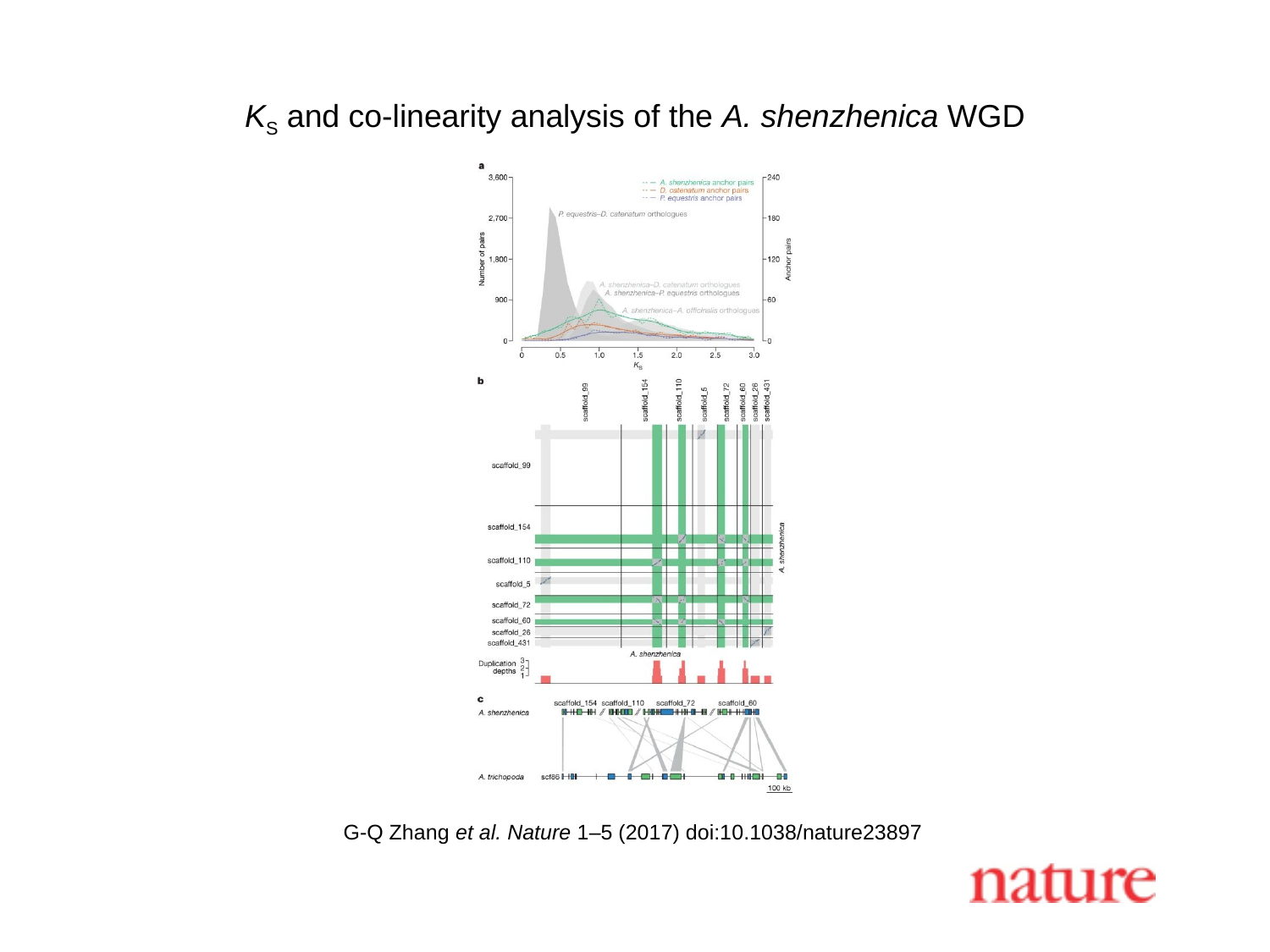

# KS and co-linearity analysis of the A. shenzhenica WGD
G-Q Zhang et al. Nature 1–5 (2017) doi:10.1038/nature23897

Supplement: Supplementary file 3 — PowerPoint slide for Fig. 2 [file 41586_2017_BFnature23897_MOESM3_ESM.ppt]

## Slide 1
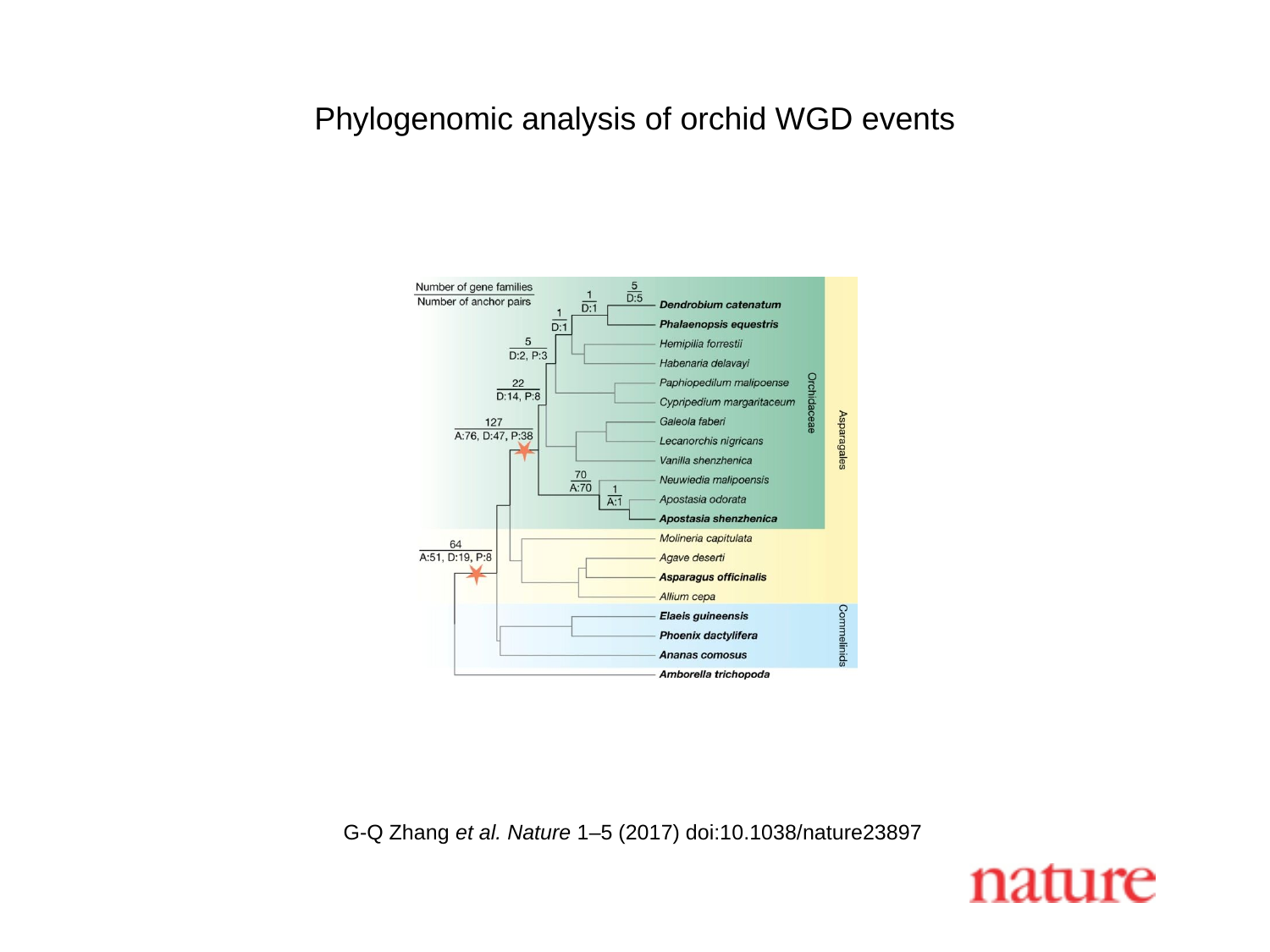

# Phylogenomic analysis of orchid WGD events
G-Q Zhang et al. Nature 1–5 (2017) doi:10.1038/nature23897

Supplement: Supplementary file 4 — PowerPoint slide for Fig. 3 [file 41586_2017_BFnature23897_MOESM4_ESM.ppt]
