# Supplementary material for: The Apostasia genome and the evolution of orchids
Source: Nature. 2017 Sep 13;549(7672):379–83. doi: 10.1038/nature23897 (PMC7416622; doi:10.1038/nature23897)
Supplement: Supplementary file 5 — PowerPoint slide for Fig. 4 [file 41586_2017_BFnature23897_MOESM5_ESM.ppt]

## Slide 1
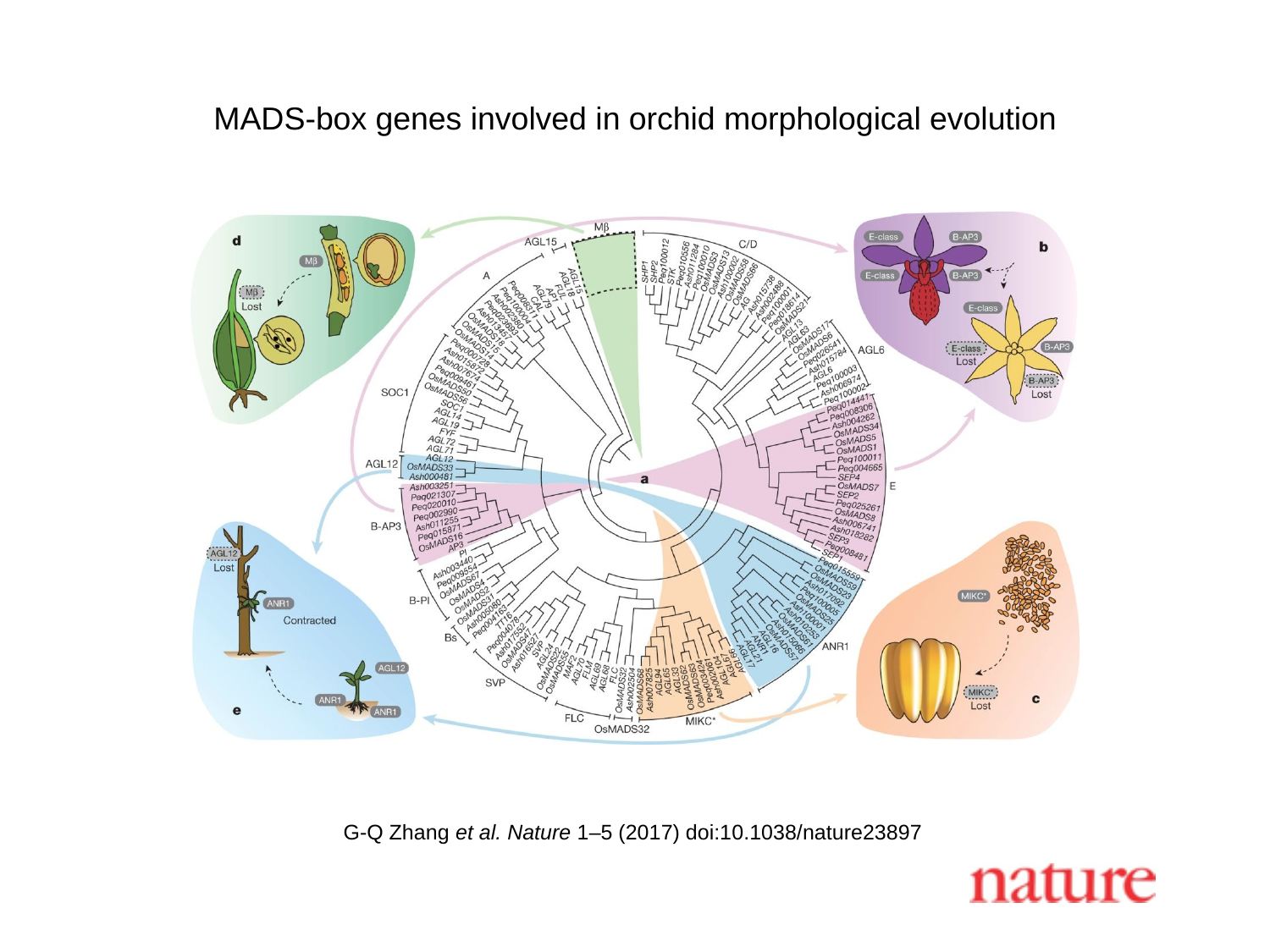

# MADS-box genes involved in orchid morphological evolution
G-Q Zhang et al. Nature 1–5 (2017) doi:10.1038/nature23897
